# Differential captivity and experiential conditions and its impact on the behaviour and cognition of Picasso triggerfish (*Rhinecanthus aculeatus*)

**DOI:** 10.1007/s10071-026-02057-1

**Published:** 2026-03-14

**Authors:** James Cordery, Nick A. R. Jones, Cait Newport

**Affiliations:** 1 Department of Biology, University of Oxford, Oxford, UK; 2 Department of Animal Physiology, University of Bayreuth, Bayreuth, Germany

**Keywords:** Cylinder test, Detour test, Emergence test, Novel object test

## Abstract

In this study, we compared performance across four behavioural tasks in the same fish species (*Rhinecanthus aculeatus*), with one group held in long-term laboratory captivity and the other recently caught and temporarily housed at a field station laboratory. The aims of this study were twofold: first, to test whether captivity conditions influence performance in commonly used behavioural and cognitive assays; and second, to evaluate whether any of these tasks could serve as a practical tool for screening behavioural changes over time. The four tests used were a Novel Object Test, Puzzle Preference Test, Emergence Test, and Cylinder Test. We found that recently caught fish were generally more exploratory and more responsive to novel stimuli; however, their responses were object-specific, with increased neophobia towards some objects. Long-term captive fish were more variable in their responses across all tests. In the Emergence Test, long-term captive fish emerged faster but showed greater individual variability. In the Cylinder Test, all recently caught fish failed to swim around a transparent cylinder, whereas several long-term captive individuals showed possible evidence of inhibitory control. Our results demonstrate that captivity conditions can influence performance in behavioural tests at both group and individual levels. These findings have important implications for comparative cognition studies, particularly when interpreting results collected across different laboratory settings or captivity durations, even when working with the same species. Of the tasks used, the Emergence Test was identified as the most practical assay for tracking the effects of captivity on behaviour, as it was highly sensitive to individual differences and straightforward to run and analyse.

**Supplementary Information:**

The online version contains supplementary material available at 10.1007/s10071-026-02057-1.

## Introduction

It is well known that environmental and experiential conditions can influence the behaviour of fish. A growing field of research has begun to systematically test a range of specific conditions to understand how they alter fish health, welfare, and behaviour (Arechavala-Lopez et al. 2022; Spence-Jones et al. 2025; Stevens et al. 2017). For instance, group size or stocking density can produce species specific differences in feeding latency, aggression, swimming speed, and exploratory activity (Anderson et al. 2022; Saxby et al. 2010; Shishis et al. 2022). Enclosure size can influence movement behaviours such as swimming speed, immobility, and turning angles (Shishis et al. 2022). The effects of environmental enrichment in the form of additional plants, structures, or shelters, can vary across species (Jones et al. 2021; Näslund and Johnsson 2016). For example, in experiments with Rainbow Trout (*Oncorhynchus mykiss*), exploratory behaviour did not differ between groups kept in sparse versus enriched tanks (Anderson et al. 2022). In contrast, juvenile salmon (*Salmo salar*) reared with a pebble substrate showed increased feeding activity compared to those kept in an empty control tank (Dunaevskaya et al. 2025). For some species, however, it is not simply the presence or absence of enrichment that matters, but the degree of variability in the environment. For juvenile Cod (*Gadus morhua*), altering the availability of food and the position of enrichment items (rocks, pebbles, plants) led to differences in exploration latency, food consumption, reaction time, and stress-recovery rates compared to a group kept under constant conditions (Braithwaite and Salvanes 2005). Together, these examples illustrate just a fraction of the many environmental and experiential influences known to shape fish behaviour, highlighting how extensive and complex the range of relevant factors can be.

The aforementioned effects extend beyond behaviour, as many of the same conditions also influence cognitive performance, intended as an individual’s ability to acquire, process, retain, and act on information, usually assessed through assays that measure learning, memory, and problem-solving (Shettleworth 2010). A growing number of studies are focusing on understanding how captivity conditions influence cognition. It has been found, for example, that barren tanks can lead to reduced spatial cognition; in separate experiments, both black rockfish (*Sebastes schlegelii*) and juvenile salmon provided with visual enrichment performed better in a maze task (Salvanes et al. 2013; Zhang et al. 2021). Factors such as social conditions (Brandão et al. 2015), food availability (Kotrschal and Taborsky 2010), access to enrichment (Strand et al. 2010; Varracchio et al. 2024), and domestication (Swaney et al. 2015) have all be shown to alter performance in associative learning and other measures of cognition. Some of these same factors, such as environmental complexity, can also alter brain and sensory system development, leading to fish with smaller brains and modified sensory neural architecture (Dunlap et al. 2011; Kihslinger et al. 2006; Kihslinger and Nevitt 2006; Marchetti and Nevitt 2003; Mayer et al. 2011; von Krogh et al. 2010).

Although it is clear that a range of conditions can influence behaviour and cognition in fish, our knowledge remains largely restricted to a small number of factors and their effects on a few species commonly used in aquaculture (e.g. salmon Brown et al. 2003; Millidine et al. 2006), cod (Strand et al. 2010)) or as scientific models (e.g. zebrafish DePasquale et al. 2016; Lee et al. 2022, cichlids (Kotrschal and Taborsky 2010). Fully exploring all factors would be extremely time-consuming, and in many cases impractical or impossible. However, even investigators not primarily focused on these factors still need to understand how much a change in behaviour might be due to the specifics of their setup, without necessarily determining the precise cause. There are many factors that may influence behaviour in experimental fish but are impossible to control, including developmental history, housing conditions, and prior experience. Understanding the magnitude of their effects is therefore essential for interpreting behavioural outcomes, even when the specific causal factors cannot be fully disentangled. This made us wonder, to what extent does the experimental context in which a species is studied influence its behaviour? Throughout this study, *experimental context* refers to the full combination of factors that are not controlled for between groups, including but not limited to testing site, provenance, past experience, age, sex, transport conditions, lighting, temperature, and tank size. Rather than isolating specific factors, we aimed to quantify the overall magnitude of behavioural differences, if they exist, between two groups of Picasso triggerfish (*Rhinecanthus aculeatus*) tested under very different conditions: one group kept in a laboratory at the University of Oxford and the other recently caught and tested at the Lizard Island Field Station in Australia. Picasso triggerfish were chosen for this study because they are a useful species for exploring behavioural and cognitive questions (Cheney et al. 2013; Newport et al. 2024; Simpson et al. 2016).

The intent of our approach is to provide a practical demonstration of the potential scale of experimental context-driven variation, even when the underlying causes are not specifically identified. The environmental and experiential factors that influence behaviour have not yet been tested in this species, therefore we had no a priori expectation about which behavioural assays might produce detectable differences in behaviour. We compared performance across four different assays that are fast and easy to implement, with the broader aim of evaluating their potential use as routine indicators of behavioural change in laboratory fish. This exploratory approach is also valuable because it establishes a baseline for our study species. If no substantial differences emerge between groups, this suggests that variation in experimental context has limited practical consequences for behavioural studies. If differences emerge only in certain assays, those assays can then serve as starting points for future studies examining which environmental or experiential factors alter behaviour. However, if large and consistent differences appear across multiple assays, this would imply that behavioural—and possibly cognitive—results may not be comparable across settings, and would indicate that there is a significant context factor that must be accounted for when designing future experiments.

The motivation for this series of experiments came from observations by CN that fish trained in the lab appeared to behave differently to those trained at a field site. Specifically, lab fish seemed slower to learn, less motivated by food rewards, and less inclined to interact with experimental stimuli. These observations formed the basis of our predicted outcomes for each experiment. To investigate this anecdotal observation, we adapted four experimental protocols commonly used across animal behaviour research: 1) Novel Object Test, 2) Puzzle Preference Test, 3) Emergence Test, and 4) Cylinder Test.

Novel object assays typically involve introducing an unfamiliar object into the animal’s environment and observing its response, such as whether it approaches or avoids the object. In fish, these assays have been used to measure curiosity and neophobia (Brown et al. 2022; Franks et al. 2023; Sneddon et al. 2003). In our study, we focused on the number of bites directed at the object as this species frequently bites any objects within its aquarium. We expected the lab fish to be slower to respond to the novel objects, suggesting increased neophobia, or perhaps reduced curiosity.

Food preference assays typically present animals with a choice between two or more food types or locations and measure their selections (Kasumyan and Sidorov 2010). In our version of the assay, fish had to push aside a movable window to access food options, introducing a puzzle-solving component. The general feeding apparatus was inspired by Lucon-Xiccato and Bisazza (2014) and Vila-Pouca et al. (2025) and is similar to other puzzle feeder designs (Gatto et al. 2024). Five different food options were presented simultaneously. Fish were allowed to consume all options within a fixed time limit, but the order in which they accessed the options was their choice. We used the first item consumed as an indicator of food preference. Fish were not given prior training on how to operate the moveable doors. Although the food was visible and likely discernible by olfactory cues through the transparent doors, individuals had to learn to move the door in order to access it. We expected the lab fish to be pickier with their food choices and to avoid the pellets, while we expected the field fish to be less discriminating and therefore choose food at random.

Emergence assays measure the time taken for an individual to leave a shelter or refuge and enter a novel or open area. In fish, emergence assays have been applied across many species to explore individual behavioural variation, often in the context of personality (Näslund et al. 2015). Emergence times also have ramifications for many cognitive tests. For example, emergence time in a maze was shown to influence engagement and learning in two model species, three-spined sticklebacks and minnows (Jones et al. 2023). In our study, we adapted this assay by conducting the test within each fish’s home tank, a modification that we believe will make the assay more efficient to run and eliminate additional handling stress to the fish. Our expectation regarding group differences in emergence was not straightforward. Laboratory fish may emerge more quickly due to greater familiarity with their environment, whereas field-caught fish could emerge sooner as a result of higher exploratory tendencies.

Finally, the Cylinder Test is a type of detour assay in which animals must move around a transparent or semi-transparent barrier to reach a visible goal, typically a food reward. Success in this assay is often interpreted as evidence of inhibitory control, or the ability to suppress a direct approach in favour of a more strategic route (Lucon-Xiccato 2024). Although widely used in mammals, the cylinder test has only recently been applied to fish (Keagy et al. 2019; Lucon-Xiccato et al. 2017; Minter et al. 2017). In our study, we incorporated the cylinder test as part of the broader *ManyFishes* initiative (Prétôt et al. 2026), which aims to explore cross-species variation in cognitive performance. We used a simple pass/fail criterion based on whether the fish made contact with the cylinder (fail) or successfully navigated around it without touching the barrier (pass). We expected laboratory-held fish to exhibit greater inhibitory control, as prolonged access to a stable food source may reduce impulsive responding and promote more deliberate movement.

## Materials and methods

### General procedure

Experiments were conducted at the University of Oxford, UK, and the Lizard Island Research Station (LIRS), Queensland, Australia. For simplicity, we refer to these sites as OXFORD and LIRS, respectively. The study species, Picasso triggerfish, is a shallow-reef fish that is typically solitary or loosely haremic in the wild and territorial in captivity. For this reason, individuals were housed separately during experiments. See **Online Resource 1** for details on individual fish, including captivity duration and experimental experience.

The fourteen individuals used in OXFORD experiments were all wild-caught but sourced from commercial suppliers. Four fish were purchased from The Goldfish Bowl (118–122 Magdalen Road, Cowley, Oxford, UK) on 13/08/20 (F46), 07/07/21 (F49), and 21/07/21 (F53, F56). Seven fish (F58, F60, F62, F63, F64, F65, F66) were obtained from De Jong Marinelife (Spijksesteeg 2A, 4212 KG Spijk Gem Lingewaal, Netherlands) and arrived at the laboratory on 21/09/2023.

Ten individuals were collected by snorkellers around Lizard Island between 09/03/24 and 11/03/24, under a Queensland Government General Fisheries Permit (256,324) and a Great Barrier Reef Marine Park Authority permit (G49627.1). To minimise selection bias, we targeted the first fish encountered and ensured that individual was caught where possible. Selected fish were captured using hand-nets and a diluted anaesthetic of clove oil solution over a three-day period. Each fish was placed in an individual plastic bag and transported to the research station by boat, which took less than 20 min. Upon arrival, fish were housed in aquaria and given 4–5 days to acclimate before experiments commenced. This duration was selected based on our previous experience with the species, as it is typically the time required for individuals to display normal feeding behaviour and cease hiding in the presence of experimenters.

At both OXFORD and LIRS, experiments were conducted in each fish’s home tank to minimise handling stress, and fish were housed individually. Fish were fed twice daily, with approximately three hours between feedings. In all experiments, fish were not fed immediately before testing, but were fed either during or after the experiment. When food rewards were part of the task, any fish that did not receive a reward during testing were fed afterwards to ensure they were not left hungry. Outside of experimental trials, they received a varied diet including Hikari Marine-A pellets (Kyorin Co., Ltd.), fish, crab, mussels, krill, and cockles.

Aquarium dimensions were comparable across sites. In OXFORD, fish were kept in 104 × 36 × 31 cm (L × W × H; ~ 100 L, excluding a small inaccessible drainage area) tanks within a flow-through marine aquarium system. Each tank included a gravel substrate, live rock, and a commercially available refuge. Water quality was monitored regularly: temperature was checked daily, while ammonia, nitrate, nitrite, salinity, and pH were measured at least weekly. Conditions were maintained within healthy ranges (ammonia = 0, nitrate < 20, nitrite = 0, specific gravity = 1.024, temperature = 25 ± 1.5 °C). Lighting followed a 12:12 h light–dark cycle with gradual ramp-up and ramp-down.

At LIRS, eight fish (fish 1–6, 8, 10) were kept in tanks measuring 79 × 36 × 38 cm (l⋅w⋅h; ~ 82L filled), and the remaining two tanks were 90 × 37 × 37 (~ 96L filled). Each tank had sand collected from the ocean, and a half-cylinder PVC refuge (l = 25 cm, r = 15 cm). Tanks were fed with water pumped directly from the ocean ensuring water chemistry levels were kept stable. The source of the pumped water was within meters of a population of *R. aculeatus*, ensuring that the water was right for this species. Temperature in the tanks was measured twice per day and ranged from 28.2—31.4°C. Temperatures were frequently compared to water temperature in the ocean to ensure they matched. Tanks were outdoors under a roof allowing fish to have natural daylight while protecting them from direct sun and rain.

At LIRS, the order of the experiments were as follows: 1) Novel Object Test, 2) Puzzle Preference Test, 3) Cylinder Test, 4) Emergence Test. At OXFORD, the order was changed so that the order of the Emergence Test and Cylinder Test were switched. To make the most efficient use of field time, training or acclimation for some experiments overlapped with testing. For example, the LIRS group completed their Cylinder Test training sessions on the same days as some of the Preference Test trials, with one conducted in the morning and the other in the afternoon. **Online Resource 1** shows a summary of individual fish dates of captivity, experimental start dates, and pre-training outcomes.

### Novel object test

**Fig. 1:**
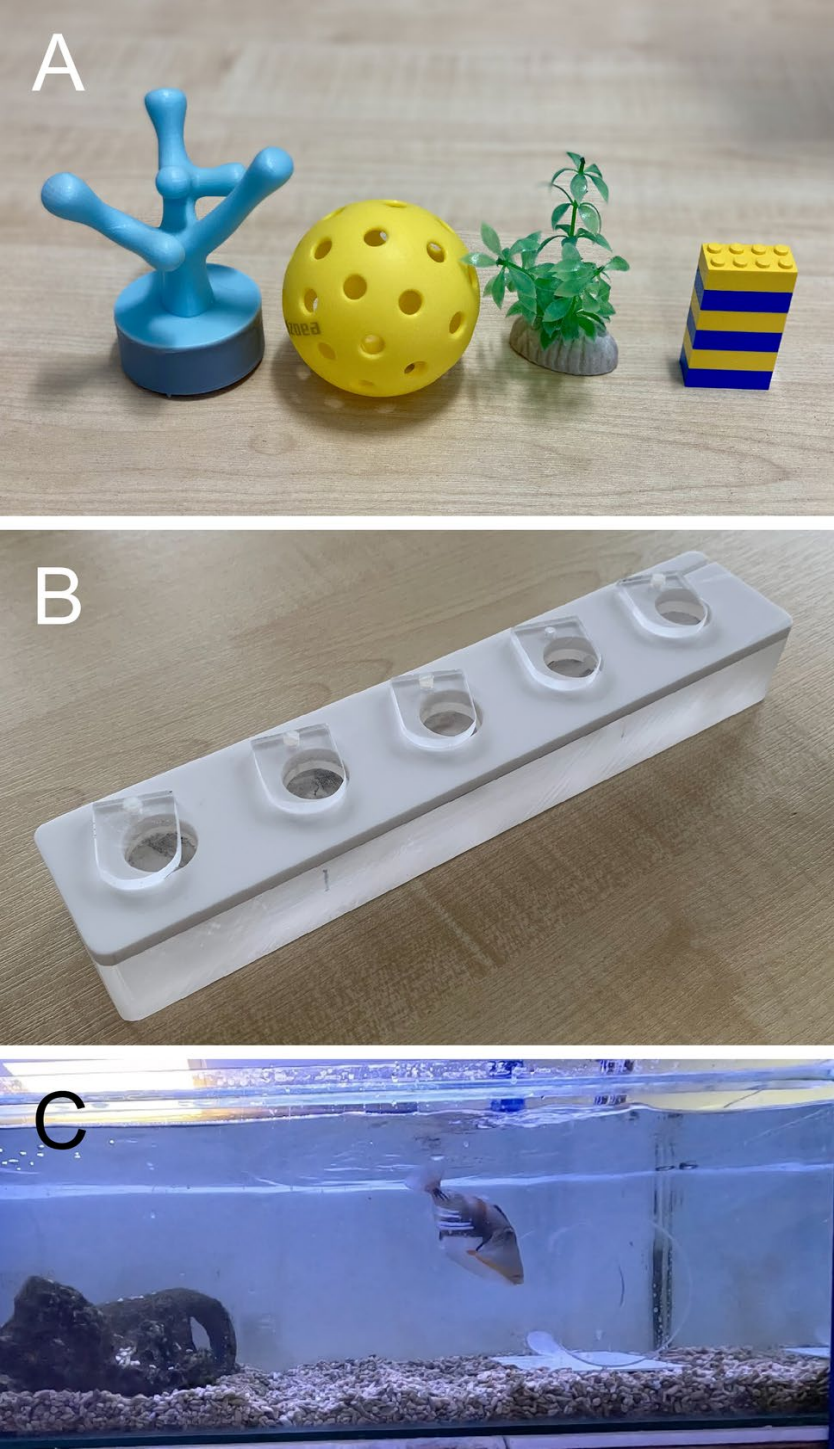
Experimental illustrations. (**A**) Images of the four objects used in the Novel Object Test. (**B**) Image of the puzzle feeder used in the Puzzle Preference test. (**C**) Video frame showing a fish (F66 trial 2) bumping into the side of the transparent cylinder. The frame has been cropped width-wise to better show the fish and does not show the full length of the tank

Ten fish were used from each site in this experiment. Each fish was presented with four trials across consecutive days (one trial/day), with a different novel object introduced in each trial (Fig. 1A). Objects included two ecologically relevant items—a 3D-printed stylized coral (day 1) and a plastic aquarium seaweed (day 3)—and two artificial items—a yellow pickleball (day 2) and a blue-and-yellow stack of six Lego bricks (day 4). While the fish was in the refuge, objects were placed at the end of the home tank opposite the occupied refuge. The object was then left for 10 min, during which fish could freely interact. Trials began once the experimenter’s hand exited the water. All trials were recorded with a GoPro HERO12 (GoPro, Inc.) and behaviours were quantified using BORIS (Friard and Gamba 2016). For each trial, the following metrics were recorded: (1) total number of bites, (2) latency to first bite, and (3) total time spent hiding in the refuge.

### Puzzle test

Twenty-four fish were tested: 10 at LIRS and 14 at OXFORD. To assess problem-solving and dietary preference, we used a two-part cognitive task comprising a box-opening phase followed by a food choice phase. Fish were first presented with a puzzle feeder made of a clear plastic base with a white top (l: 250 × w: 34 × h: 50 mm). Five equally spaced wells (20 mm diameter, 20 mm apart) were covered by transparent swivel doors that had to be pushed aside to open (Fig. 1B). Each well contained a single food item (~ 10 mm pieces). The moveable doors were incorporated to slightly increase the effort required to access the food, encouraging fish to work harder for items they valued more and potentially making their preferences more pronounced. The doors were intentionally easy to move, minimising the likelihood that smaller individuals had to exert more effort than larger ones.

**Phase 1: Solving the puzzle feeder.** This phase was used to habituate the fish to the apparatus and procedure. Only shrimp was used as a food reward during this phase, and it was placed in every well to keep the value of each position consistent. Each fish received one trial per day for five days. They were given up to 10 min to retrieve food from the device; if all food was consumed earlier, the trial ended. Fish were considered successful if they opened at least one door on at least four out of five days. If fish did not interact (i.e. approach or bite) with the apparatus throughout the 10-min trials for two consecutive trials, they were removed from the experiment. For each trial we recorded if the fish passed, failed, or had no interaction.

**Phase 2: Food Choice.** Following successful completion of Phase 1, fish progressed to the food choice task. Here, all five food types—mussel, squid, shrimp, fish (mackerel), and Hikari Marine-A pellets—were presented simultaneously in the wells of the puzzle feeder. Although newly caught fish may have been less familiar with some food types, all fish had tasted each type of food as part of regular feeding prior to the experiment. This species is omnivorous and generally willing to eat a wide range of foods. With the exception of the pellets, we selected food types that these fish could feasibly encounter in their natural environment. Because the doors were clear and not sealed in any way, the fish had access to odour and visual cues. The order of the food types in each well was randomized for each trial using a random number generator. Trials began once the experimenter’s hand left the tank and lasted up to 10 min or until all food items were consumed. Each fish was tested once daily for 15 days, except for one individual at LIRS (F8) which only completed 12 replicates before being released early for welfare reasons. At LIRS, fish were tested on consecutive days but at OXFORD, trials occurred only on weekdays. The order of consumption was recorded live and later verified with video analysis.

### Emergence test

Twenty fish were included in this experiment, with 10 from each location. All individuals completed three replicate trials, but fish F56 only had two recorded. A medium grey acrylic box (390 × 360 × 310 mm, open-topped) was placed at the end of the home tank opposite the shelter. Each fish was gently guided into the box and confined for a 3-min period. The front door was then raised, and emergence latency was recorded. All fish were tested once daily for three consecutive days.

Emergence latency was defined as the time between the door being halfway open, when the fish could begin to exit, and the point at which the base of the caudal fin (black spines) was fully visible. These spines were chosen as the emergence marker because they are more visible and rigid than the translucent, flexible fin. Lateral-view video recordings were used to determine timing.

### Cylinder test

Twenty-three fish participated in this experiment. The test was adapted from the *ManyFishes* project (Prétôt et al. 2026), and the procedures were replicated as closely as possible. The experiment consisted of four sequential phases: 1) Plate training, 2) Cylinder familiarisation, 3) ‘Forced’ cylinder trial, and 4) Testing. Phases 1–3 served to familiarise the fish with the components of the task, while Phase 4 was the final test. Trials from Phases 1, 3, and 4 were recorded. While we aimed to match the protocol as closely as possible, one deviation from the *ManyFishes* protocol was the distance between the cylinder and the starting compartment, which was shorter in our experiment. Additionally, there was minor variation in tank sizes across individual fish as previously described.

**Phase 1. Plate Training**: Two plexiglass dividers, a grey opaque one and transparent one, were placed side-by-side in the tank, dividing it into two compartments. The two dividers were arranged so that the opaque divider could be lifted while the clear divider remained briefly in place. This setup allowed the fish a moment to view the task before having direct access to it, preventing them from rushing forward immediately when the opaque barrier was removed. The fish began each trial on the side with a shelter, confined to roughly one-third of the tank’s total volume. A white plastic food plate (16.5 × 13.3 cm) was positioned in the larger section of the tank. At LIRS, the plate was either 10 cm (small tank) or 30 cm (larger) from the end of the tank. The distance between the dividers and the food plate was approximate and varied slightly between trials; overall, this distance appeared to be shorter in the OXFORD experiments compared to those conducted at LIRS. A green dot marked the food location on the plate to increase its saliency.

Each trial began with the removal of the opaque divider, followed by the transparent one, allowing the fish to view the scene for approximately 3 s before it could approach the food. The fish could then swim freely to the plate and feed on a small piece of shrimp placed on the green dot. Shrimp was chosen because it has previously been used as a reward for this species. Each fish underwent six trials in total—three per session, with one session in the morning and one in the afternoon. Fish were given a maximum of 5 min per trial to consume the food. If the food was not eaten within this time, the plate was removed, and a new trial began. Subjects progressed to Phase 2 only if they successfully ate the food in five out of six trials. At OXFORD, 13 fish participated in Phase 1. Of these, four individuals (F46, F56, F59, F61) did not meet the criterion for progression (i.e. consuming the food from the plate within 5 min) and were excluded from subsequent phases. In contrast, all 10 fish tested at LIRS successfully completed Phase 1. Most fish completed six replicates; however, one trial for F6 was excluded due to a technical recording error, leaving five trials for that individual.

All trials were recorded at 60 frames per second (fps) at LIRS and 30 fps in OXFORD. For each trial, we recorded: (A) whether the fish consumed the food (i.e., trial success), and (B) the latency to eat, calculated as the difference between the trial start and end time. The trial start time was defined as the frame in which the transparent divider was removed sufficiently for the fish to move forward. This was necessary because fish often attempted to push past the divider before it was fully removed. Trial end time was recorded when the fish consumed the food; even when the plate was not entirely visible in the video, this moment could be reliably identified from fish movement.

**Phase 2. Cylinder Familiarisation:** A transparent cylinder (diameter: 15cm, length: 15 cm) was placed against the wall farthest from the shelter, centred to allow entry from either side. There was no food reward within the cylinder during this phase. Fish were allowed to freely explore the cylinder for 1 h per session, on two consecutive days. This is shorter than the 48-h familiarisation recommended by the ManyFishes protocol. However, our preliminary observations indicated that the species used in this study explored novel objects quickly but habituated rapidly, so longer exposure does not generally increase interaction rates.

**Phase 3. Forced Cylinder Trial:** This phase combined elements from Phases 1 and 2. As in Phase 1, the tank was divided using an opaque and a transparent board. However, the transparent board now had a hole cut to fit the clear cylinder, which was inserted such that it acted as a passage between compartments. The food plate was placed on the far side of the cylinder. Upon removing the opaque divider, fish could pass through the cylinder to access the food. The transparent divider ensured the fish could see the food reward, and that it was forced to go through the cylinder in order to reach the reward. To proceed to Phase 4, each subject had to successfully enter and exit the cylinder at least once, regardless of whether they consumed the food. Fish were given a maximum of 5 min.

All nine fish from OXFORD that successfully reached this stage, passed the test and went through the cylinder. At LIRS, eight fish passed, but two of them (F8 and F10) required repeated trials after managing to squeeze through a gap between the tank and board. In both cases, the trials were reset and repeated successfully. Fish F9 did not pass this stage, as it failed to go through the cylinder in both of its allotted trials (maximum 5 min each). Fish F1 was initially recorded as having passed the test by the experimenter (JC). However, upon reviewing the video, it became clear the fish had not actually gone through the cylinder. Due to this experimenter error, F1 was not given another replicate and instead continued to Phase 4.

**Phase 4. Testing: **In the final testing phase, the clear cylinder was placed directly over the food plate, such that the white square and green dot remained visible. The food was placed within the cylinder but over the green dot as in previous phases. The food remained visible but could only be accessed by detouring around the transparent barrier of the cylinder. The tank configuration and use of opaque and transparent dividers remained the same as in Phase 1. Each fish completed 10 test trials across two consecutive days (5 trials per day). For each trial, we recorded whether the fish made direct contact with the cylinder (classified as a test failure, Fig. 1C) or successfully navigated around it without touching the barrier (classified as a test success). Eighteen fish reached the final phase of the experiment, with nine individuals from each location. All fish completed all ten replicates.

After completion of the experiment, a methodological artefact was noticed in all OXFORD trials, that represents a major limitation to this experiment. The removal of the board often shifted the fish toward the sides of the tank, causing them to approach the cylinder from a non-central angle. This sideward deflection was caused by the physical structure of the OXFORD tanks, which all have a horizontal lip at the top for supporting lids. To accommodate this edge, the dividers had a notch cut at the top, allowing them to fit around the lip. Removing these dividers required a slight twisting motion. Because the OXFORD fish tended to wait pressed directly against the divider, this twisting motion frequently moved the fish toward the tank sides—a pattern not observed at LIRS, where tanks lacked such lid supports.

### Statistical analysis

All statistical analyses were conducted in R (R Core Team 2024). Data were analysed using Generalised Linear Mixed Models (GLMMs) implemented via the glmmTMB package (Brooks et al. 2017; McGillycuddy et al. 2025). The response variable differed by experiment and is specified in the respective results sections. Fixed effects always included Location (either LIRS or OXFORD), while random effects typically accounted for individual-level variability by including individual identity (Fish ID) as a random intercept.

Model selection followed a parsimonious, diagnostics-driven approach. Initial models started with a minimal fixed-effects structure and were assessed using the DHARMa package (Hartig 2022) to evaluate model assumptions and fit (e.g., residual distribution, overdispersion, zero inflation). Where assumptions weren’t met, alternative models were explored by adjusting the distribution (e.g. negative binomial when Poisson is overdispersed) or transforming the response variable. Interaction terms were tested for statistical significance using a likelihood-ratio test (LRT), and those found to be non-significant and not essential to the primary effects of interest, were removed from the final model. Where multiple models met diagnostic criteria, the model with the lowest Akaike Information Criterion (AIC) was selected. Table 1 summarises the final models used for each of the four assays. Individual behavioural repeatability was estimated using the rptR package (Nakagawa and Schielzeth 2010).

**Table 1:** Summary of the mixed-effects models used for all experiments

Model	Response	Type	Predictors	Model structure	N
*Novel Object Test*					
M1	Number of bites	Negative binomial GLMM	Location * Object type	Random = Fish ID; ZI = ~ 1	80
M2	Latency to bite	Gamma GLMM	Location * Object type	Random = Fish ID	54
M3	Time spent in refuge	Negative binomial GLMM	Location * Object type	Random = Fish ID	70
*Puzzle Test*					
M4	Food selection count	Negative binomial GLMM	Location * Food item		73
*Emergence Test*					
M5	Emergence time (log-transformed)	Gaussian LMM	Location	Random = Fish ID; Dispersion = ~ Fish ID	59
*Cylinder Test*					
M6	Trial time (log-transformed)	Gaussian GLMM	Location * Replicate	Random = Replicate, Fish ID; Dispersion = ~ Replicate	113

Model results were interpreted directly, or where group-level comparisons were of interest, we used Estimated Marginal Means (EMM) and pairwise comparisons via the emmeans package (Lenth 2024). Results from EMMs are reported as ratios of expected values between LIRS/OXFORD. A ratio of 1 indicates no difference, values less than 1 indicate lower values in the numerator group, and values greater than 1 indicate higher values. To correct for multiple testing across object-level contrasts, we applied a simultaneous inference correction using the general linear hypothesis testing framework (glht) from the multcomp package (Hothorn et al. 2008), which controls the family-wise error rate.

All R code used for data exploration, model fitting, and figure generation is provided in the supplementary materials: (Novel Object Test: **Online Resource 2**; Puzzle Test: **Online Resource 3**; Emergence Test: **Online Resource 4**; Cylinder Test: **Online Resource 5**).

## Results

### Novel object test

#### Total number of bites

**Fig. 2:**
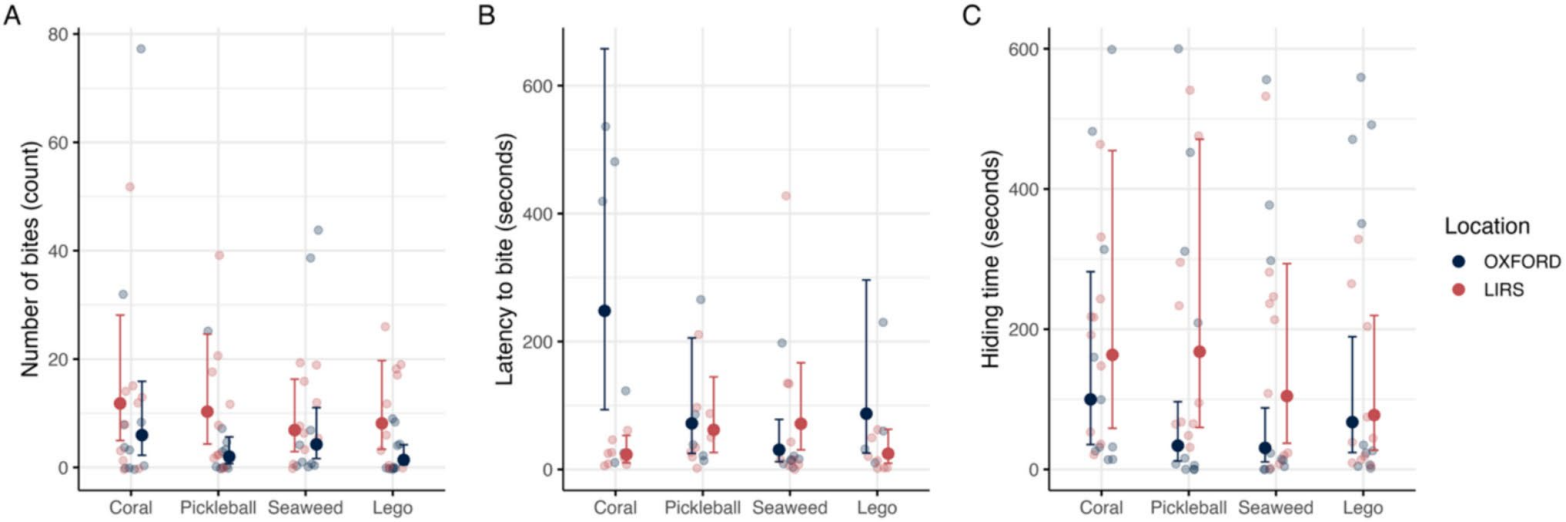
Novel Object Task. Model-estimated responses are shown for (**A**) total number of bites, (**B**) latency to first bite, and (**C**) total time spent hiding, comparing fish from LIRS and OXFORD across four object types. Estimated marginal means are plotted with 95% confidence intervals derived from the fitted models. Faint, jittered points represent individual raw data values to illustrate sample size and variability within groups

We tested how the total number of bites differed across object type and between locations (M1). We found a significant interaction between location and object type on the number of bites (LRT: χ^2^(3) = 8.25; *p* = 0.04). Including fish identity as a random intercept significantly improved model fit (LRT: χ^2^(1) = 28.12; *p* < 0.001), indicating significant among-individual variation. We found that fish at LIRS showed a significantly higher number of total bites compared to OXFORD for the Lego Stack (*ratio* = 5.81, 95%CI = [1.45 23.21], *z* = 2.49, *p* = 0.01) and Pickleball objects (*ratio* = 5.13, 95%CI = [1.33 19.73], *z* = 2.38, *p* = 0.02) (Fig. 2A). However, there was no statistically significant difference in location for the Coral (*ratio* = 1.98, 95%CI = [0.54 7.28], *p* = 0.31) or Seaweed (*ratio* = 1.62, 95%CI = [0.45 5.76], *p* = 0.46).

#### Latency to first bite

Trials in which fish did not bite were removed from this analysis as it was impossible to measure latency in these trials. We first tested whether the proportion of trials with zero bites differed by location using a chi-squared test for equality of proportions. The test showed a significant difference (χ2 (1) = 4.62, *p* = 0.032), with 20% of LIRS trials and 45% of OXFORD trials removed due to zero biting.

We examined how latency to bite varied across object types and between locations (M2). We found a significant interaction between location and object type on the latency (LRT: χ2(3) = 13.38; *p* = 0.004). Including fish identity as a random intercept did not significantly improved model fit (LRT: χ2(1) = 3.36; *p* = 0.24), indicating no significant among-individual variation. We found the latency to bite was significantly shorter at LIRS compared to OXFORD for the coral (ratio = 0.094, 95% CI [0.026, 0.336], p < 0.001) (Fig. 2B). No significant differences were observed for the Lego Stack (ratio = 0.283, p = 0.105), Pickleball (ratio = 0.860, p = 0.825), or Seaweed (ratio = 2.33, p = 0.176) objects. Notably, coral was also the first object presented to all subjects, suggesting display order may matter.

#### Total time spent hiding in the refuge

We examined how the duration of time spent hiding in the refuge differed across object types and between locations (M3). Although the interaction between location and object was not significant (LRT: χ2(3) = 6.64; *p* = 0.08), it was retained in the model to facilitate interpretation of the main effect. Including fish identity as a random intercept significantly improved model fit (LRT: χ2(1) = 38.18; *p* < 0.001), indicating significant among-individual variation. After adjusting for multiple comparisons, we found that the time spent hiding was significantly longer at LIRS compared to OXFORD for the Seaweed (*ratio* = 4.96, 95% CI [1.14, 21.56], *p* = 0.033) (Fig. 2C). However, no significant location differences were observed for Coral (*ratio* = 1.64, *p* = 0.508), Pickleball (*ratio* = 1.15, *p* = 0.853), or Lego Stack (*ratio* = 3.42, *p* = 0.102).

### Puzzle test

**Phase 1: Solving the puzzle feeder.** At LIRS, six fish successfully opened the puzzle feeder doors during the first 10-min trial, while the remaining four learned to do so in the second trial. Similarly, ten fish at Oxford passed within the first trial, while one fish required two trials, and three fish never passed at all. We tested whether the pass rates in the first trial were statistically different between the two groups using a Fisher’s Exact Test, but found no statistically significant difference (p = 0.67).

All LIRS fish advanced to Phase 2, however, at OXFORD, three fish did not interact with the apparatus during either of the two 10-min trials and were excluded from further testing. Therefore 11 Oxford fish advanced to Phase 2.

**Phase 2: Food choices.** To assess food preferences, we analysed each fish’s first choice in the food preference task. Most individuals completed 15 trials, resulting in 15 first-choice data points per fish, with the exception of one individual (F8), which completed 12 trials. First-choice frequencies were tallied across experimental locations, revealing Fish and Mussel as the most frequently selected items (Fish: LIRS = 40%, OXFORD = 23%; Mussel: LIRS = 32%, OXFORD = 38%). Pellet was never chosen first by any fish at LIRS, resulting in structural zeros that precluded model convergence; consequently, Pellet was excluded from further statistical analysis.

**Fig. 3:**
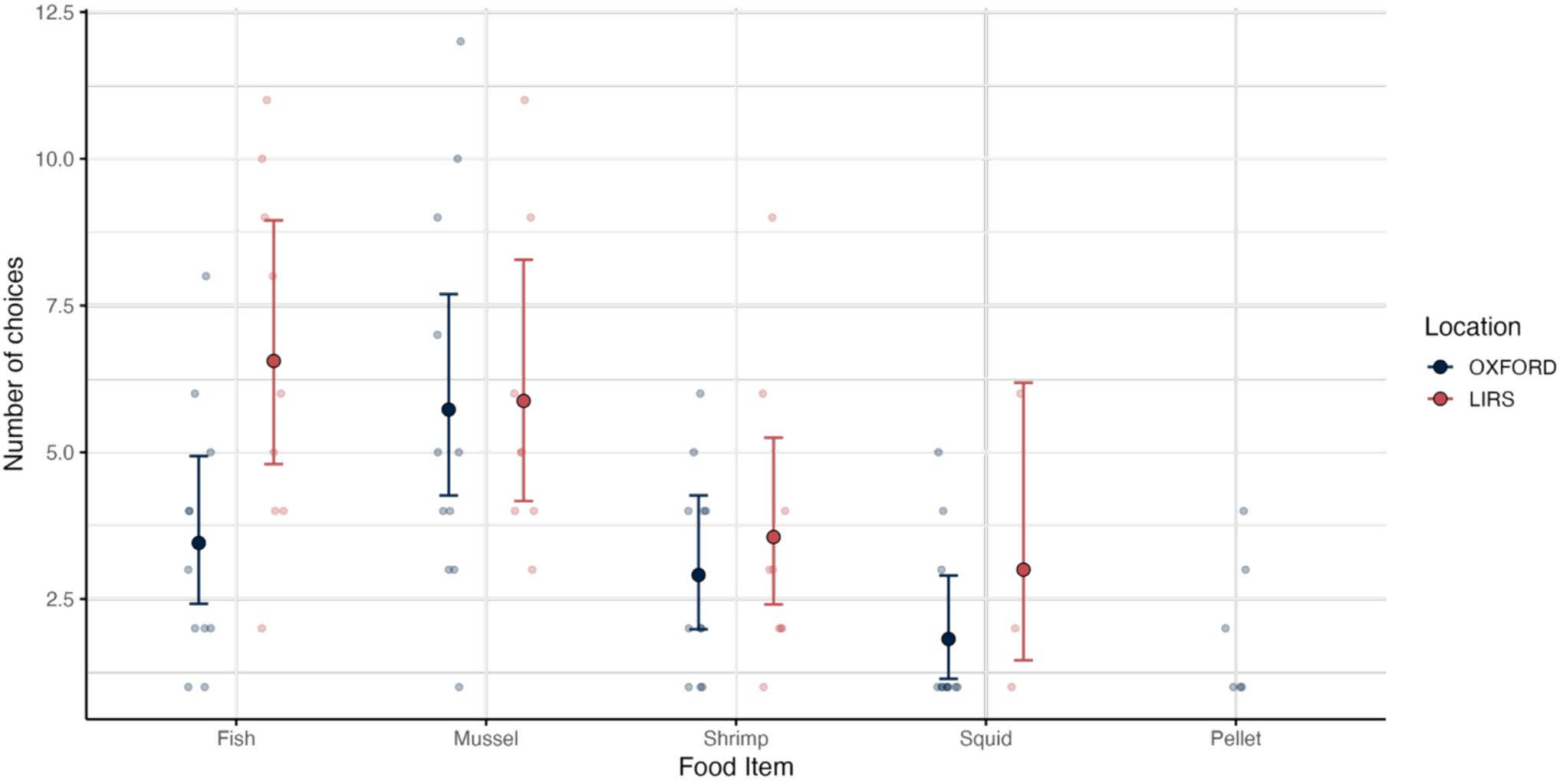
Food Preferences. Number of first-choice selections for each food type across 15 replicates, comparing fish from LIRS and OXFORD. Estimated marginal means from the fitted model are shown with 95% confidence intervals. Faint, jittered points represent individual data to illustrate sample size and within-group variability

We tested for differences in food preference across locations and food types (M4). Although the interaction between location and food type was not significant (LRT: χ2(3) = 3.64; *p* = 0.30), it was retained in the model to facilitate interpretation of the main effect. Including fish identity as a random intercept did not significantly improve model fit (LRT: χ2(1) = 1; *p* = 1), and was dropped from the model. We found a significant main effect of location, with fish from OXFORD showing a different overall pattern of food preferences compared to those from LIRS (estimate = -0.64, SE = 0.24, *z* = -2.65, *p* = 0.008; Fig. 3). Among LIRS fish, there was no significant difference in the number of first-choice selections between fish and mussels (*p* = 0.64). Shrimp, however, was chosen significantly less often than fish (estimate = -0.61, SE = 0.25, *z* = -2.40, *p* = 0.016), and squid showed a trend toward being chosen less frequently (estimate = -0.78, SE = 0.40, *z* = -1.95, *p* = 0.052). We found that OXFORD fish chose mussels first significantly more often than fish (estimate = 0.51, SE = 0.24, *z* = 2.14, *p* = 0.030), while shrimp were chosen at the same frequency as fish (estimate = -0.17, SE = 0.27, *z* = -0.64, *p* = 0.52). Squid was chosen significantly less often than fish (estimate = -0.64, SE = 0.30, *z* = -2.14, *p* = 0.030).

### Emergence test

**Fig. 4:**
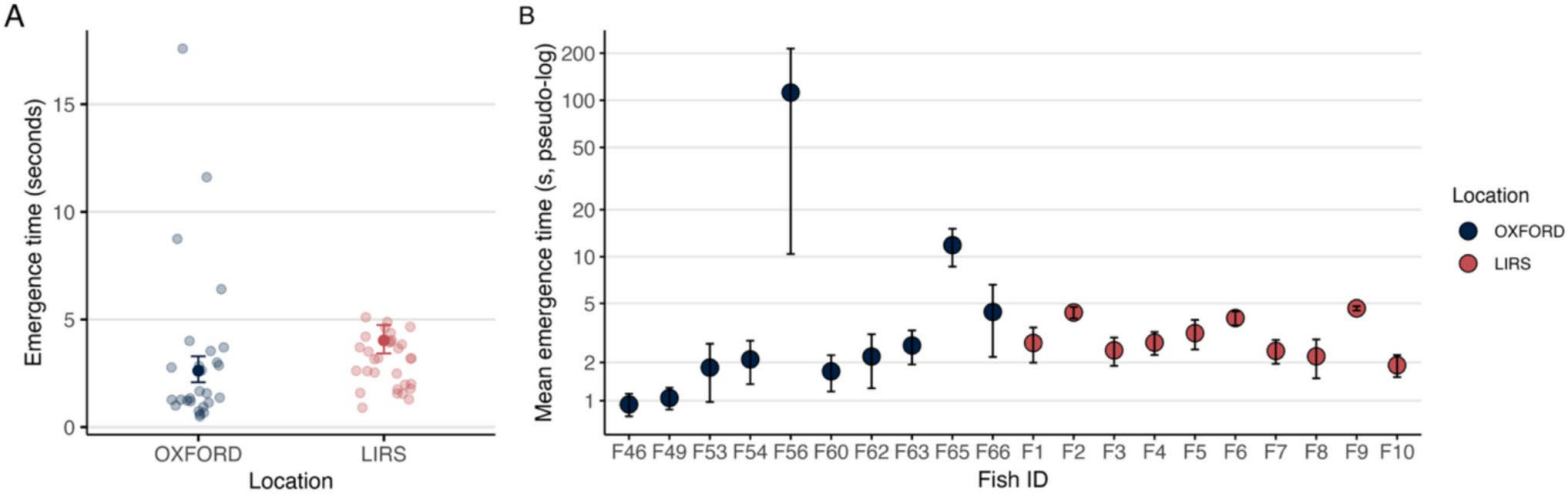
Emergence Test. (**A**) Model-estimated mean emergence times by location from Model 5. Pale dots represent raw trial-level data, and error bars show 95% confidence intervals. The maximum value for fish F56 (emergence time = 214 s) is excluded to improve visual clarity. (**B**) Raw mean emergence times per individual, grouped by location. Error bars indicate within-individual standard error. A pseudo-log scale was used to display low and high values on the same axis

We tested for differences in emergence time and behavioural consistency between locations (M5). The main effect we found is that OXFORD fish emerged significantly faster than LIRS fish (estimate = –0.43 ± 0.14, *p* = 0.002), corresponding to an emergence time approximately 35% shorter after back-transformation (Fig. 4A).

This model also revealed substantial individual variation. Including fish identity as a random intercept significantly improved model fit (LRT: χ2(1) = 7.85; *p* < 0.01), indicating significant among-individual variation. Allowing the dispersion (residual variance) to vary also improved model fit (LRT: χ2(20) = 81.05; *p* < 0.001), indicating that the variability in emergence time was not constant across observations. Together, these results indicate that emergence time varies both among individuals and in the degree of within-individual variability. We found fish F56 and F65 were significantly more variable (*p* < 0.01 and *p* = 0.019), while F9 was more consistent than average (*p* = 0.002). The results of the repeatability analysis further support this finding as it showed that emergence time had moderate individual consistency (R = 0.513, 95% CI: 0.19–0.74, *p* < 0.003). Figure 4B shows the raw mean emergence time for each individual, highlighting differences in behavioural variability between individuals and locations.

### Cylinder test

**Phase 1: Plate Training.** At OXFORD, 13 fish participated in Phase 1. Of these, four individuals (F46, F56, F59, F61) did not meet the criterion for progression (i.e. consuming the food from the plate within 5 min) and were excluded from subsequent phases. In contrast, all 10 fish tested at LIRS successfully completed Phase 1. To assess whether success rates differed between locations, we compared the proportion of fish that passed the task. At LIRS, 10 out of 10 fish (100%) passed, whereas at OXFORD, 9 out of 13 fish (69%) passed. Given the small sample size and the presence of a zero count in one cell (no failures at LIRS), we used a Fisher’s Exact Test. The test revealed that the difference in success rates between sites was not statistically significant (p = 0.10), indicating no strong evidence for a location-based effect on Phase 1 performance.

We then tested whether fish from different locations differed in how long they took to approach and eat food from a plate (M6). We found a significant interaction between location and replicate number (χ2(1) = 8.15; *p* = 0.004). Allowing both random intercepts and random slopes of replicate number for each fish significantly improved model fit (likelihood ratio test: χ2(3) = 103.16, *p* < 0.001), indicating substantial among‐individual differences in both baseline trial duration and the effect of replicate number. Despite trying many model options, our final model showed some residual misfit: the KS test remained significant (p = 0.00042), and DHARMa flagged quantile deviations in the mid and upper ranges of the residual distribution. These diagnostics suggest that some aspect of the data structure remained unaccounted for.

**Fig. 5:**
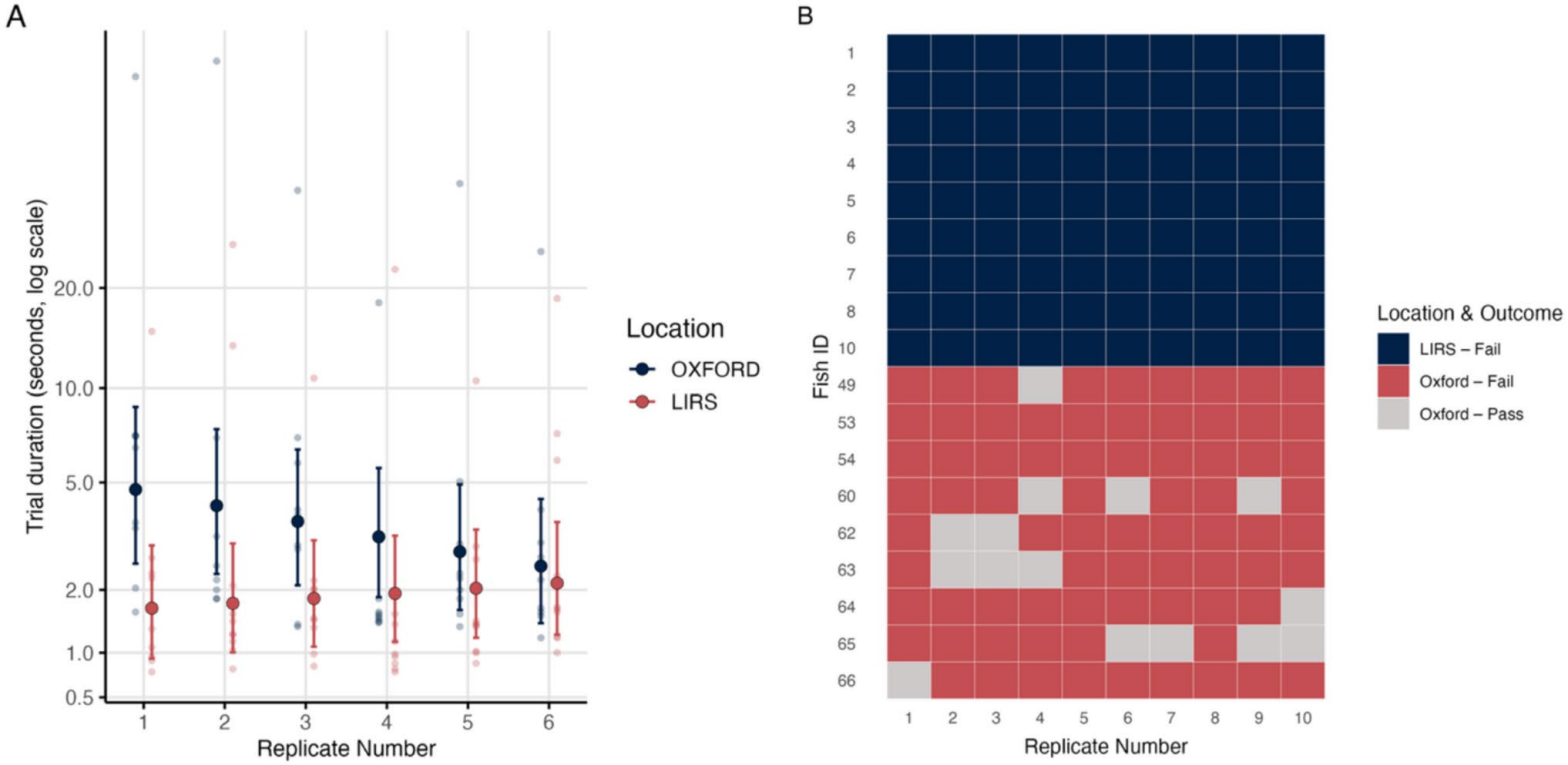
Cylinder Task. (**A**) Trial duration (in seconds) for Phase 1: Plate Acclimation. Bars show back-transformed model-based means with 95% confidence intervals. Light coloured dots represent raw data points for individual trials. A log scale was used to allow higher values to fit within the plot while preserving detail at the lower end of the range. (**B**) Success and failure outcomes during Phase 4: Testing, showing by individual and replicate number

The main effect we found was that there was a statistically significant difference in trial duration between fish from OXFORD and LIRS (Fig. 5A). At the start of the experiment, OXFORD fish took longer to reach and consume the food compared to LIRS fish (Estimate = 1.22, *p* = 0.007). However, OXFORD fish showed a steeper reduction in trial time across replicates (interaction: Estimate = –0.18, *p* = 0.001), indicating a stronger improvement with repeated exposure. Residual variance also decreased significantly across replicates (β = –0.11, *p* = 0.045), consistent with reduced behavioural variability over time.

**Phase 4: Testing.** None of the LIRS fish passed the detour test, as each individual made contact with the side of the cylinder during their approach (Fig. 5B). At OXFORD, seven of nine fish achieved at least one successful pass, resulting in 15 successful trials. Using a Fisher’s Exact Test, we compared the number of fish that passed at least one trial per group and found a highly statistically significant difference (p < 0.001).

However, as described in the Methods, there was a potential methodological artefact that may have influenced fish trials at Oxford. Of the 15 successful trials at Oxford, we suspect thirteen trials may have been influenced by the way the divider board was removed. Of the two trials where the dividers did not appear to influence the trial, in one case (F64, Trial 10), the fish appeared to pass the test by swimming along the tank wall, seemingly guided by a piece of food that had drifted to the edge of the cylinder. Only one trial at OXFORD (F63, Trial 4) showed clear evidence of successful detour behaviour: the fish did not wait near the divider and swam across the front of the cylinder before entering from the side, all without making contact with the cylinder. Under a more conservative interpretation, assuming only one Oxford fish genuinely passed the test, the difference between groups is not statistically significant (Fisher’s Exact Test: p = 0.47) (Fig. 5).

## Discussion

Across all four assays, we found consistent differences between fish tested at LIRS and OXFORD, demonstrating that experimental context has a significant influence on behaviour and cognition. These differences were evident in responses to novel stimuli, food preferences, emergence behaviour, and possibly detour or inhibition behaviour, indicating that context effects are not restricted to a particular type of assay. We interpret the location-based differences as the cumulative result of multiple, co-varying aspects of experimental context rather than the influence of any single factor. Differences in provenance, prior experience, housing conditions, transport, and testing environments are all likely contributors, but disentangling their individual effects was not the aim of this study. Instead, our results provide a practical estimate of the magnitude of behavioural variation that can arise when such factors differ simultaneously. Although the direction and magnitude of effects varied across tests, their presence across all assays underscores the importance of considering experimental context when interpreting behavioural data, even when experimental assay protocols are standardised.

The direction of effects differed by assay. In the novel object test, we predicted that OXFORD fish would be slower to interact with unfamiliar objects; this was broadly supported, as LIRS fish showed higher bite rates and shorter latencies for some stimuli. However, LIRS fish also displayed stronger object-specific neophobic responses, indicating that reactions depended on stimulus type. In the puzzle test, we expected OXFORD fish to be more selective than LIRS fish. We found that both groups had clear preferences, but these preferences differed by location: LIRS fish preferred mackerel and mussels, whereas OXFORD fish favoured mussels over mackerel, with pellets least preferred by both groups and never chosen first by LIRS fish. For the emergence test, predictions were less directional, as laboratory familiarity and field-related exploratory tendencies could plausibly act in opposite directions. We found that OXFORD fish emerged approximately faster than LIRS fish, suggesting higher exploratory behaviour or reduced hesitation. Finally, in the cylinder test we expected OXFORD fish to show greater inhibitory control, but we found that all LIRS fish failed the detour criterion, while most OXFORD fish passed at least one trial, albeit inconsistently (but see below for experimental limitations).

Another key result was the substantial individual variation in behaviour observed across assays, which in some cases, differed between locations. Individual identity explained a significant proportion of the variation in both the novel object and emergence tests, and emergence time showed moderate repeatability, indicating consistent individual differences over time. This variation was particularly evident in the emergence assay: for example, individual F56 emerged significantly more slowly than other fish, with a maximum emergence time of 214 s—more than 80 times the group mean for OXFORD fish (2.62 s). In contrast, F65 at OXFORD showed significantly greater within-individual variability in emergence time than the population mean, whereas individuals at LIRS (e.g. F9) were significantly more consistent. However, individual differences were not detected across all assays or behaviours, suggesting that behavioural variation may be task-specific. For instance, although we observed location-based differences in food preference, we did not find significant individual-level variation in this trait. It seems likely that assays in which behaviour is underpinned by personality traits or behavioural syndromes—such as boldness, exploration-avoidance, activity, aggressiveness, and sociability (Conrad et al. 2011)—are more likely to show individual variation.

Several factors may underpin the differences observed between groups across our experiments. Although we cannot be certain given the number of variables that differed between locations, we outline here some plausible explanations and highlight directions for future study. In the novel object assay, LIRS fish both bit and hid more frequently, suggesting that they were generally more active and responsive to the stimuli. One possible explanation is the warmer water temperature experienced by LIRS fish (3-6ºC higher than OXFORD), which may have increased boldness, as has previously been shown in zebrafish (Angiulli et al. 2020). However, temperature alone does not readily explain the object-specific increase in neophobia observed in LIRS fish. Instead, this pattern suggests differences in how the objects themselves were perceived. We selected blue and yellow objects because *R. aculeatus* show sensory biases towards this colour combination (Cheney et al. 2013); these colours also transmit well in coral reef environments and are associated with a range of biologically relevant signals, from aposematism (Marshall et al. 2019) to cleaning services (Cheney et al. 2009). It is therefore possible that differences in past experience, or the recency of those experiences, shaped whether individuals perceived these objects as attractants or deterrents.

In the puzzle preference test, differences in food familiarity may have contributed to the observed group variation in food preference. OXFORD fish are routinely fed a variety of seafood including fish pieces, whereas LIRS fish – despite exposure during acclimation – are unlikely to consume mackerel in the wild and therefore needed more time to acclimate. Mussels, by contrast, likely represent a more ecologically relevant food source for both groups. These differences highlight the importance of considering the perceived value of a reward and tailoring food type used as a reward to individuals or populations being tested. While pellets offer consistency in size and nutritional content, they may represent a low-value reward, potentially reducing motivation during experimental tasks. More broadly, variation in reward preferences and approach behaviour underscores motivation as a key, but often hidden, variable in behavioural experiments. Tasks that are intended to measure cognitive ability may instead reflect differences in hunger, reward valuation, or prior experience with particular food types. Therefore, empirically assessing reward value or tailoring rewards to specific populations should be considered an integral component of experimental design.

The Emergence Test measured the time it took individuals to leave a shelter; differences in this behaviour may reflect an individual’s boldness and exploration tendencies. OXFORD fish emerged from the box 35% faster on average than LIRS fish. However, they also showed significantly more behavioural variability across trials. Emergence tests are known to be highly variable and sensitive to minor methodological differences or environmental factors that are challenging to isolate, particularly when sample sizes and the number of replicate trials are small (Beckmann and Biro 2013). For example, a study using three- and nine-spine sticklebacks from the same location, tested using the same emergence protocol in consecutive years, produced qualitatively and quantitatively different results (Näslund 2021). Acclimation time can strongly influence repeatability; in guppies, repeatability of emergence scores was highest after a maximum acclimation duration of 2 h (O’Neill et al. 2018). Fish size and motivation (level of satiation) can also play a role – tests with guppies showed that larger individuals can be slower to emerge (Brown et al. 2007). Collectively, our results as well as evidence from previous studies, show that emergence time is strongly influenced by experimental context. Because emergence time reflects differences in boldness, motivation, and exploration, these differences may cascade into large differences in apparent cognitive performance between groups (Jones et al. 2024; Kurvers et al. 2018).

Finally, we used the Cylinder Test to assess if experimental context could influence inhibitory control; however, both limitations of the test itself and an experimental error constrains the conclusions that can be made. A key limitation is the binary pass-fail outcome, which limits the nuance of the data, making it difficult to detect differences between groups or to track individual performance over time. For example, although all LIRS fish failed the test, we cannot draw strong conclusions about individual abilities or the reasons for failure. One possibility is that this species lacks inhibitory control entirely. Inhibitory control refers to the ability to supress a behaviour in order to obtain a preferred reward (Loyant et al. 2025). In our experiment, demonstrating inhibitory control would require suppressing the impulse to swim directly toward the visible food and instead detour around the transparent barrier. In natural contexts, inhibitory control may play a role in social interactions and has been associated with complex group dynamics (Amici et al. 2008; Ashton et al. 2018; Johnson-Ulrich and Holekamp 2020). One way inhibitory control may support group dynamics is by enabling lower-ranked individuals to supress feeding or mating behaviours in the presence of more dominant individuals (Amici et al. 2018). In this context, the social dynamics of Picasso triggerfish suggest a similar potential requirement for inhibitory control. While this species is territorial, it is also loosely-haremic and defended territories often overlap with those of conspecifics. From personal observations, individuals sometimes traverse the territories of dominant conspecifics, yet they seem to avoid the areas when the dominant individual is nearby and presumably within range of attack. In addition, dominant individuals will attack some intruders while tolerating others within their harem, even when harem members and intruders are of similar size. This suggests that access to space is mediated by social relationships rather than simple size asymmetries. For subordinate individuals, movement within this social system may require supressing the impulse to enter a profitable area when the risk of aggression is high, and this restraint may be underpinned by inhibitory control.

Evidence from other fish species also suggest that at least some teleost’s have inhibitory control (Brandão et al. 2019; Santacà et al. 2019; Triki and Bshary 2021). Therefore, while our experiments indicate it is possible this species lacks inhibitory control, other explanations for failure may be more plausible. One alternative is that the fish did not understand the task. In many cylinder test paradigms, animals are first habituated to reach food from the sides of an opaque cylinder before being tested with a transparent one (Kabadayi et al. 2018). While fish in our experiments were allowed to explore the transparent cylinder, this exposure does not replicate habituation to the full task. With more training or experience, the fish may have learned to detour successfully. A further limitation stems from the OXFORD tank design, which influenced how the divider was removed, and may have influenced the path of the fish. Nonetheless, we decided to include the results here because despite all OXFORD trials being subject to the same potential bias, not all individuals passed the test. Had the divider fully dictated behaviour, we would have expected all fish to ‘pass’ in every trial, but that wasn’t the case. Without repeating the experiment, a process which would preclude direct comparison with the LIRS group, it remains unclear whether some *R. aculeatus* individuals are capable of inhibitory control, and whether this differs among the groups, or if the observed pattern are artifacts of our experimental procedure.

Together these results highlight several important considerations for experimental design and interpretation in comparative cognition research. For starters, they reinforce the concept that experimental context has a large influence on behaviour and thus on the assessment of cognitive abilities. While enrichment or captivity experiments, for example, can identify some contextual effects, it is impossible to fully quantify the cumulative impact of all potential differences between groups. The factors we measure, and control for, are ultimately constrained by the experimenters imagination. Because cognition is underpinned by behaviour, comparisons between groups that differ not only at the species level but also in experimental context may inadvertently capture contextual effects rather than cognitive differences, even when protocols are standardised. Across our experiments, groups generally exhibited similar behaviours, but differed in the magnitude of their responses and the degree of individual variation. We therefore propose that measuring variation prior to comparing cognitive performance is essential. Such data can then inform power analyses and experimental design for subsequent cognitive tests. Three of the assays in this study – Novel Object, Puzzle Preference, and Emergence tests—are useful for this purpose. Each requires minimal equipment and fish training, while capturing group-level differences and individual variation. However, each test has its own strengths and weaknesses. The novel object test was effective at detecting group-level differences, but may be less informative when repeated over time as fish become more habituated. In addition, as there is no clear expectation for how individuals should respond to different objects, it is difficult to make any predictions about how fish will respond to each object. In contrast, the Emergence test is particularly effective for detecting individual variation and repeatability and was the easiest to run and analyse. Finally, as previously discussed, the Puzzle Preference test is useful for assessing the perceived value of rewards. Looking forward, we believe integrating these behavioural assays into the early stages of cognitive studies can improve the interpretability of findings, ensuring that contextual factors are appropriately accounted for.

In summary, our results demonstrate that even within the same wild-caught species, detectable behavioural differences can emerge depending on the experimental and experiential conditions of the group. These differences are likely shaped by a range of factors including housing conditions, diet, enrichment, transportation methods, and motivational state. Longer periods in captivity may further increase exposure to such influences and compound over time. These findings have important implications for comparative cognition studies, particularly when interpreting results collected across different laboratory settings. Experimental context should thus be carefully considered in the design and interpretation of cognitive experiments.

## Supplementary Information

Below is the link to the electronic supplementary material.

Supplementary file1 (XLSX 11 KB)

Supplementary file2 (XLSX 26 KB)

Supplementary file3 (XLSX 11 KB)

Supplementary file4 (XLSX 14 KB)

Supplementary file5 (HTML 4249 KB)

Supplementary file6 (HTML 2106 KB)

Supplementary file7 (HTML 2929 KB)

Supplementary file8 (HTML 3188 KB)

Supplementary file9 (XLSX 23 KB)

## Data Availability

All data and R code are included as Online Resources.
