# Supplementary material for: Differential captivity and experiential conditions and its impact on the behaviour and cognition of Picasso triggerfish (Rhinecanthus aculeatus)
Source: Anim Cogn. 2026 Mar 14;29(1):33. doi: 10.1007/s10071-026-02057-1 (PMC13002752; doi:10.1007/s10071-026-02057-1)
Supplement: Supplementary file 5 — Supplementary file5 (HTML 4249 KB) [file 10071_2026_2057_MOESM5_ESM.html]

Novel Object Test Analysis


# Novel Object Test Analysis

#### Cait Newport

#### 2025-06-05

# 1 Project Description

This analysis is part of the data processing pipeline for the
**Novel Object Test**, a behavioral assessment used in the
associated manuscript: *Behavioural Differences Across Captivity
Conditions in Triggerfish (Rhinecanthus aculeatus): Implications for
Cognitive Testing*.

The current notebook focuses on the following components of the
behavioral dataset:

- **Total Bites**: Number of times each fish bit the
  object.
- **Latency to Bite**: Time elapsed from start of the
  trial to first bite.
- **Total Time Spent Hiding**: Total duration during
  which the fish remained in a hiding state.

# 2 Environment Initialisation

## 2.1 Load required libraries

```
library(readxl)
library(dplyr)
library(ggplot2)
library(lme4)
library(emmeans)
library(DHARMa)
library(multcomp)
library(glmmTMB)
```

```
## Warning in checkDepPackageVersion(dep_pkg = "TMB"): Package version inconsistency detected.
## glmmTMB was built with TMB version 1.9.11
## Current TMB version is 1.9.14
## Please re-install glmmTMB from source or restore original 'TMB' package (see '?reinstalling' for more information)
```

```
library(patchwork)
library(tidyr)
library(purrr)
```

## 2.2 Load Complete Dataset Files

This chunk loads all CSV files from the data folder into the
environment for analysis.

```
# Define file path
file_path <- "/Users/user/projects/CaptiveCognition/NovelObjectTest/novel_object_data2.xlsx"

# Read the Excel file
total_bites_data <- read_excel(file_path)

# Print the first few rows (header) of the data
head(total_bites_data)
```

```
## # A tibble: 6 × 6
##    Fish Object     TotalBites Group  Latency_to_Bite HidingTime
##   <dbl> <chr>           <dbl> <chr>            <dbl>      <dbl>
## 1    49 Coral               8 OXFORD          536.         32.0
## 2    49 Pickleball          7 OXFORD           21.5        15.9
## 3    49 Seaweed             7 OXFORD            3.00        0  
## 4    49 Lego Stack          0 OXFORD           NA          23.2
## 5    53 Coral               0 OXFORD           NA          13.7
## 6    53 Pickleball          0 OXFORD           NA           0
```

```
# Show fish used in the experiment
fish_by_group <- total_bites_data %>%
  dplyr::select(Fish, Group) %>%
  distinct() %>%
  arrange(Group, Fish) %>%
  group_by(Group) %>%
  summarise(Fish_IDs = paste(sort(unique(Fish)), collapse = ", ")) %>%
  pivot_wider(names_from = Group, values_from = Fish_IDs)

# View the table
print(fish_by_group)
```

```
## # A tibble: 1 × 2
##   LIRS                          OXFORD                                
##   <chr>                         <chr>                                 
## 1 1, 2, 3, 4, 5, 6, 7, 8, 9, 10 49, 53, 54, 56, 58, 59, 61, 62, 63, 66
```

```
# Count how many rows had TotalBites == 0, grouped by Group
removed_by_group <- total_bites_data %>%
  filter(TotalBites == 0) %>%
  group_by(Group) %>%
  summarise(Rows_Removed = n(), .groups = "drop")

print(removed_by_group)
```

```
## # A tibble: 2 × 2
##   Group  Rows_Removed
##   <chr>         <int>
## 1 LIRS              8
## 2 OXFORD           18
```

# 3 Total Bites Analysis

## 3.1 Visualize Bite Count Distribution with Histogram

```
ggplot(total_bites_data, aes(x = TotalBites, fill = Group)) +
  geom_histogram(position = "stack", bins = 15, color = "black", alpha = 0.8) +
  scale_fill_manual(values = c("OXFORD" = "#002147", "LIRS" = "#C44E52")) +
  labs(
    title = "Stacked Histogram of Total Bites by Fish Group",
    x = "Total Bites",
    y = "Frequency",
    fill = "Fish Group"
  ) +
  theme_minimal(base_size = 14) +
  theme(
    plot.title = element_text(hjust = 0.5, face = "bold"),
    legend.position = "top",
    axis.text = element_text(size = 12)
  )
```

## Assessing Zero Inflation in Total Biting Behavior

We evaluated whether the distribution of total bites exhibited zero
inflation — a common issue in behavioral count data when non-responses
(i.e., no bites) are frequent.

In the full dataset, **26 out of 80 observations (32.5%) were
zero**, indicating a moderate level of zero inflation:

```
table(total_bites_data$TotalBites == 0)
```

```
## 
## FALSE  TRUE 
##    54    26
```

```
mean(total_bites_data$TotalBites == 0)
```

```
## [1] 0.325
```

Although this proportion is not excessive, it is substantial enough
to warrant further investigation.

## 3.2 Testing Distributional Assumptions for GLMMs of Biting Counts

To identify the most appropriate model for analyzing variation in
total biting behavior (`TotalBites`), we compared several
generalized linear mixed models (GLMMs). These models were chosen to
account for both the count-based nature of the response variable and the
hierarchical structure of the data (i.e., repeated measures across
individual fish).

Because biting counts are discrete, potentially zero-inflated, and
overdispersed, we compared four models: - A standard Poisson GLMM
(glmm\_pois) - A Negative Binomial GLMM (glmm\_nb) - A Zero-Inflated
Poisson (ZIP) GLMM (glmm\_zip) - A Zero-Inflated Negative Binomial (ZINB)
GLMM (glmm\_zinb)

Models were compared using the Akaike Information Criterion (AIC) to
assess relative fit.

```
# Standard Poisson GLMM (assumes mean = variance)
glmm_pois <- glmmTMB(TotalBites ~ Group + Object + (1 | Fish), family = poisson, data = total_bites_data)

# Negative Binomial GLMM (accounts for overdispersion)
glmm_nb <- glmmTMB(TotalBites ~ Group * Object + (1 | Fish), data = total_bites_data, family = nbinom2)

# Zero-Inflated Poisson GLMM (accounts for excess zeros)
# model zero-inflation probability with intercept-only
glmm_zip <- glmmTMB(TotalBites ~ Group + Object + (1 | Fish), ziformula = ~1, family = poisson, data = total_bites_data)

# Zero-Inflated Negative Binomial GLMM (accounts for both overdispersion and zero inflation)
glmm_zinb <- glmmTMB(TotalBites ~ Group * Object + (1 | Fish), ziformula = ~1, family = nbinom2, data = total_bites_data)

# Compare models using AIC (lower is better)
AIC(glmm_pois, glmm_zip, glmm_nb, glmm_zinb)
```

```
##           df      AIC
## glmm_pois  6 678.7755
## glmm_zip   7 564.2779
## glmm_nb   10 482.4693
## glmm_zinb 11 469.9157
```

The Zero-Inflated Negative Binomial (ZINB) model produced the lowest
AIC, indicating the best fit to the data of the four models. This
supports the conclusion that both overdispersion and excess zeros are
meaningful features of the dataset.

All subsequent models therefore use the ZINB distribution.

## 3.3 Assessing the Effect of Object Identity on Biting Behavior

**Objective:** This section tests whether including
`Object` as a predictor improves model fit — either as: a) An
interaction with `Group` (i.e., does object preference depend
on whether a fish is wild or captive?), or  
b) A main effect, independent of group (i.e., are some objects more
likely to be bitten regardless of group?).

```
# Model 1: Baseline model with only Group (Captive vs Wild)
# This serves as the simplest model to compare against.
model_no_object <- glmmTMB(TotalBites ~ Group + (1 | Fish), ziformula = ~1, family = nbinom2, data = total_bites_data)

# Model 2: Full model with Group, Object, and their interaction
# Tests whether object identity affects biting, and whether that effect differs by group.
model_with_object <- glmmTMB(TotalBites ~ Group * Object + (1 | Fish), ziformula = ~1, family = nbinom2, data = total_bites_data)

# Model 3: Intermediate model with only Group and Object (no interaction)
# Tests whether objects differ in biting rate, independent of group.
model_object_only <- glmmTMB(TotalBites ~ Group + Object + (1 | Fish), ziformula = ~1, family = nbinom2, data = total_bites_data)

AIC(model_no_object,model_with_object,model_object_only)
```

```
##                   df      AIC
## model_no_object    5 471.5743
## model_with_object 11 469.9157
## model_object_only  8 472.1614
```

```
# Likelihood Ratio Test: Full model vs Group-only model
# This tests whether adding both Object and the Group:Object interaction improves fit.
anova(model_no_object, model_with_object, REML = FALSE)
```

```
## Data: total_bites_data
## Models:
## model_no_object: TotalBites ~ Group + (1 | Fish), zi=~1, disp=~1
## model_with_object: TotalBites ~ Group * Object + (1 | Fish), zi=~1, disp=~1
##                   Df    AIC    BIC  logLik deviance  Chisq Chi Df Pr(>Chisq)  
## model_no_object    5 471.57 483.48 -230.79   461.57                           
## model_with_object 11 469.92 496.12 -223.96   447.92 13.659      6    0.03369 *
## ---
## Signif. codes:  0 '***' 0.001 '**' 0.01 '*' 0.05 '.' 0.1 ' ' 1
```

```
# Likelihood Ratio Test: Main-effects model vs Group-only model
# This tests whether Object has a significant main effect, regardless of interaction.
anova(model_no_object, model_object_only, REML = FALSE)
```

```
## Data: total_bites_data
## Models:
## model_no_object: TotalBites ~ Group + (1 | Fish), zi=~1, disp=~1
## model_object_only: TotalBites ~ Group + Object + (1 | Fish), zi=~1, disp=~1
##                   Df    AIC    BIC  logLik deviance Chisq Chi Df Pr(>Chisq)
## model_no_object    5 471.57 483.48 -230.79   461.57                        
## model_object_only  8 472.16 491.22 -228.08   456.16 5.413      3     0.1439
```

```
# Likelihood Ratio Test: Full model vs Main-effects model
# This directly tests whether the Group × Object interaction significantly improves model fit.
anova(model_object_only, model_with_object, REML = FALSE)  # <-- SIGNIFICANT interaction if p < 0.05
```

```
## Data: total_bites_data
## Models:
## model_object_only: TotalBites ~ Group + Object + (1 | Fish), zi=~1, disp=~1
## model_with_object: TotalBites ~ Group * Object + (1 | Fish), zi=~1, disp=~1
##                   Df    AIC    BIC  logLik deviance  Chisq Chi Df Pr(>Chisq)  
## model_object_only  8 472.16 491.22 -228.08   456.16                           
## model_with_object 11 469.92 496.12 -223.96   447.92 8.2457      3     0.0412 *
## ---
## Signif. codes:  0 '***' 0.001 '**' 0.01 '*' 0.05 '.' 0.1 ' ' 1
```

Model comparison using AIC and likelihood ratio tests indicates that
model\_with\_object has the lowest AIC.

## 3.4 Assessing Model Fit Using Simulated Residuals

We evaluated the adequacy of model\_with\_object using the DHARMa
package. Simulated residuals are used to test for key assumption
violations—including overdispersion, zero-inflation, and non-random
residual structure—through diagnostic plots and statistical tests.

```
# Simulate residuals for diagnostic tests
simulation_output <- simulateResiduals(fittedModel = model_with_object, n = 1000)

# Plot residuals: expected vs observed, QQ plot, and residual vs predictor diagnostics
# Look for patterns, skew, or deviations from uniformity
plot(simulation_output)
```

Model diagnostics revealed no evidence of overdispersion or residual
structure violations, and the observed number of zero values was
consistent with model expectations, indicating that the selected model
adequately captured the underlying distributional properties of the
data.

```
# Test for interaction of FishID

# Use model_with_object for comparsion
#model_with_object <- glmmTMB(TotalBites ~ Group * Object + (1 | Fish), ziformula = ~1, family = nbinom2, data = total_bites_data)
model_with_object_no_fish<-glmmTMB(TotalBites ~ Group * Object, ziformula = ~1, family = nbinom2, data = total_bites_data)

anova(model_with_object,model_with_object_no_fish)
```

```
## Data: total_bites_data
## Models:
## model_with_object_no_fish: TotalBites ~ Group * Object, zi=~1, disp=~1
## model_with_object: TotalBites ~ Group * Object + (1 | Fish), zi=~1, disp=~1
##                           Df    AIC    BIC  logLik deviance  Chisq Chi Df
## model_with_object_no_fish 10 496.03 519.85 -238.02   476.03              
## model_with_object         11 469.92 496.12 -223.96   447.92 28.118      1
##                           Pr(>Chisq)    
## model_with_object_no_fish               
## model_with_object          1.141e-07 ***
## ---
## Signif. codes:  0 '***' 0.001 '**' 0.01 '*' 0.05 '.' 0.1 ' ' 1
```

## 3.5 Evaluating Group Differences in Total Biting Behavior Across Objects

To determine whether total biting behavior differs between Location
and Object, we use estimated marginal means (EMMs), with model-adjusted
means that account for other predictors in the model.

The model used: model\_with\_object TotalBites ~ Group \* Object + (1 |
Fish)

**NOTE: The negative binomial (nbinom2) distribution in glmmTMB uses
a log link function by default. This means the model estimates effects
on the log scale. Using type=“response” in the emmeans function,
back-transforms the results.**

```
# Compute estimated marginal means (EMMs) for each Group, within each Object level
emm_group_by_object <- emmeans(model_with_object, ~ Group | Object, type = "response", level = 0.95)

# Convert EMM results to a data frame for inspection or plotting
emm_df <- as.data.frame(emm_group_by_object)

# Perform pairwise comparisons of Group within each Object
pairwise_contrast <- contrast(emm_group_by_object, method = "pairwise")

# Summarize contrasts and include confidence intervals (set infer = c(TRUE, TRUE))
contrast_df <- summary(pairwise_contrast, infer = c(TRUE, TRUE)) |> as.data.frame()

# View the resulting contrast estimates with associated uncertainty
contrast_df
```

```
## Object = Coral:
##  contrast         ratio       SE  df asymp.LCL asymp.UCL null z.ratio p.value
##  LIRS / OXFORD 1.976236 1.314792 Inf 0.5364550  7.280213    1   1.024  0.3059
## 
## Object = Lego Stack:
##  contrast         ratio       SE  df asymp.LCL asymp.UCL null z.ratio p.value
##  LIRS / OXFORD 5.808008 4.105078 Inf 1.4534539 23.208826    1   2.489  0.0128
## 
## Object = Pickleball:
##  contrast         ratio       SE  df asymp.LCL asymp.UCL null z.ratio p.value
##  LIRS / OXFORD 5.127412 3.525795 Inf 1.3322356 19.734010    1   2.377  0.0174
## 
## Object = Seaweed:
##  contrast         ratio       SE  df asymp.LCL asymp.UCL null z.ratio p.value
##  LIRS / OXFORD 1.616896 1.048052 Inf 0.4538832  5.759970    1   0.741  0.4585
## 
## Confidence level used: 0.95 
## Intervals are back-transformed from the log scale 
## Tests are performed on the log scale
```

### 3.5.1 Testing for an interaction between Object and Location

```
drop1(model_with_object, test="Chisq")
```

```
## Single term deletions
## 
## Model:
## TotalBites ~ Group * Object + (1 | Fish)
##              Df    AIC    LRT Pr(>Chi)  
## <none>          469.92                  
## Group:Object  3 472.16 8.2457   0.0412 *
## ---
## Signif. codes:  0 '***' 0.001 '**' 0.01 '*' 0.05 '.' 0.1 ' ' 1
```

## 3.6 Estimated Group Differences in Biting Behavior by Object (Ratios & Significance)

This plot visualizes the estimated group differences in total biting
behavior between Captive and Wild fish for each object type, based on
pairwise comparisons from the emmeans analysis.

Each point represents the estimated bite ratio (Captive/Wild) for a
given object, and vertical bars show the 95% confidence interval for
that estimate. These ratios are back-transformed from the log scale to
the response scale for interpretability. The underlying model used a log
link function, so group comparisons were conducted on the log scale and
exponentiated to produce interpretable ratios.

The dashed horizontal line at y = 1 represents the null hypothesis of
no difference between groups. Ratios below 1 indicate that Captive fish
bit less than Wild fish for a given object, while ratios above 1
indicate that Captive fish bit more. A group difference is considered
statistically significant at the 0.05 level if the confidence interval
does not cross 1.

```
ggplot(contrast_df, aes(x = Object, y = ratio)) +
  geom_hline(yintercept = 1, linetype = "dashed", color = "gray50") +
  
  geom_point(size = 3, color = "#4C72B0") +
  geom_errorbar(aes(ymin = asymp.LCL, ymax = asymp.UCL), 
                width = 0.2, color = "#4C72B0") +

  labs(
    title = "Ratio of Estimated Bites (Captive / Wild) by Object",
    x = "Object",
    y = "Estimated Bite Ratio ± 95% CI"
  ) +
  theme_minimal(base_size = 14) +
  theme(
    plot.title = element_text(hjust = 0.5, face = "bold"),
    axis.text.x = element_text(angle = 0, hjust = 0.5)
  )
```

## 3.7 Plot A: Estimated Total Bites by Group and Object (with Raw Data Overlay)

This plot displays both raw data and model-derived estimates of total
biting behavior across object types, separated by fish group (Captive
vs. Wild).

- The **faint jittered points** represent individual
  fish-level data for total bites, allowing visualization of the
  underlying distribution and variability within each object.
- The **larger colored points** show the estimated
  marginal means (EMMs) for each group within each object, derived from
  the fitted model.
- The **vertical bars** represent the 95% confidence
  intervals for these estimated means.
- Colors distinguish between Captive and Wild fish, and horizontal
  x-axis labels identify the object types.

```
# Label levels for plotting
object_levels <- c("Coral", "Pickleball", "Seaweed", "Lego Stack")
total_bites_data$Object     <- factor(total_bites_data$Object,     levels = object_levels)
emm_df$Object               <- factor(emm_df$Object,               levels = object_levels)

p_bites <- ggplot() +
  # Raw data layer
  geom_jitter(data = total_bites_data,
              aes(x = Object, y = TotalBites, color = Group),
              alpha = 0.3, size = 2, width = 0.15, show.legend = FALSE) +

  # EMM points (mean estimates)
  geom_point(data = emm_df,
             aes(x = Object, y = response, color = Group),
             position = position_dodge(width = 0.5), size = 3,show.legend = TRUE) +

  # CI error bars (use asymp.LCL and asymp.UCL from emmeans output)
  geom_errorbar(data = emm_df,
                aes(x = Object, ymin = asymp.LCL, ymax = asymp.UCL, color = Group),
                position = position_dodge(width = 0.5), width = 0.2, show.legend = FALSE) +
  
  # 🔧 Dummy invisible points to ensure both group levels are in the legend
  geom_point(data = data.frame(
  Object = factor(c("Coral", "Coral"),
                  levels = c("Coral", "Pickleball", "Seaweed", "Lego Stack")),
  response = 0,
  Group = factor(c("OXFORD", "LIRS"),
                 levels = c("OXFORD", "LIRS"))),
                  aes(x = Object, y = response, color = Group),
                  alpha = 0, size = 0, inherit.aes = FALSE) +

 # Color mapping
# scale_color_manual(
#    values = c("OXFORD" = "#002147", "LIRS" = "#C44E52"),
#    drop = FALSE) +
  
  # Labels
  labs(
    title = "Estimated Total Bites by Group and Object",
    x = NULL,
    y = "Number of bites (count)",
    color = "Fish Group"
  ) +

  # Theme adjustments
  theme_minimal(base_size = 14) +
  theme(
    plot.title = element_text(hjust = 0.5, face = "bold"),
    axis.text.x = element_text(angle = 0, hjust = 0.5),  # Horizontal labels
    #    legend.position = "top",
    axis.line = element_line(color = "black", linewidth = 0.5),
    axis.ticks = element_line(color = "black")
  )

p_bites
```

## 3.8 Multiple Comparisons Adjustment Using `multcomp`

To evaluate the significance of group differences within each object,
we first calculated pairwise contrasts using `emmeans`.
However, because we are conducting multiple comparisons (one for each
object), the risk of Type I error increases.

To correct for this, we use the `multcomp` package’s
general linear hypothesis testing (`glht`) framework to
adjust p-values across all group comparisons. This ensures that any
reported significance reflects a more conservative, statistically
rigorous interpretation.

```
# Generate pairwise contrasts: LIRS vs OXFORD within each Object

emm_group_object <- emmeans(model_with_object, pairwise ~ Group | Object)

# Convert emmeans contrast results to a glht object for simultaneous inference
glht_obj <- as.glht(emm_group_object$contrasts)

# Summarize the results: estimates, SEs, z-values, and adjusted p-values
# Adjustments control the family-wise error rate (single-step method by default)
#summary(glht_obj) ## These results are not used because they are in log scale!
```

Back-Transform results. The back-transformed results are in the
column Ratio and CI\_Lower, CI\_Upper

```
# Z critical value for 95% CI
z_crit <- qnorm(0.975)

# Extract summary stats and compute back-transformed values
glht_summary_table <- map_dfr(names(glht_obj), function(obj_name) {
  s <- summary(glht_obj[[obj_name]])  # run summary on each glht object
  
  est <- s$test$coefficients
  se <- s$test$sigma
  z <- s$test$tstat
  p <- s$test$pvalues
  
  # Back-transform to response scale (exp for log link)
  ratio <- exp(est)
  ci_lower <- exp(est - z_crit * se)
  ci_upper <- exp(est + z_crit * se)
  
  tibble(
    Object = gsub("Object = ", "", obj_name),
    Log_Estimate = est,
    Std_Error = se, # should not be back-transformed
    z_value = z, # should not be back-transformed
    p_value = p,
    Ratio = ratio,
    CI_Lower = ci_lower,
    CI_Upper = ci_upper
  )
})

# View the result
glht_summary_table
```

```
## # A tibble: 4 × 8
##   Object     Log_Estimate Std_Error z_value p_value Ratio CI_Lower CI_Upper
##   <chr>             <dbl>     <dbl>   <dbl>   <dbl> <dbl>    <dbl>    <dbl>
## 1 Coral             0.681     0.665   1.02   0.306   1.98    0.536     7.28
## 2 Pickleball        1.76      0.707   2.49   0.0128  5.81    1.45     23.2 
## 3 Seaweed           1.63      0.688   2.38   0.0174  5.13    1.33     19.7 
## 4 Lego Stack        0.481     0.648   0.741  0.459   1.62    0.454     5.76
```

# 4 Latency to Bite

## 4.1 Remove trials with No Bites

Rows where the fish did not bite have been removed as they will not
have a time measured. A total of **26 rows were removed**.

```
total_bites_no_zeros <- total_bites_data %>%
  filter(TotalBites > 0)

# Count total rows removed
num_removed <- total_bites_data %>%
  filter(TotalBites == 0) %>%
  nrow()
cat("Total rows removed:", num_removed, "\n")
```

```
## Total rows removed: 26
```

```
# Count how many rows had TotalBites == 0, grouped by Group
removed_by_group <- total_bites_data %>%
  mutate(Removed = TotalBites == 0) %>%
  group_by(Group) %>%
  summarise(
    Rows_Removed = sum(Removed),
    Total_Rows = n(),
    .groups = "drop"
  )

# Count number of observations per Object type
observations_by_object <- total_bites_data %>%
  group_by(Object) %>%
  summarise(
    Total_Observations = n(),
    Zero_Bites = sum(TotalBites == 0),
    Some_Bites = sum(TotalBites != 0),
    .groups = "drop"
  )
# View the summary table
print(removed_by_group)
```

```
## # A tibble: 2 × 3
##   Group  Rows_Removed Total_Rows
##   <chr>         <int>      <int>
## 1 LIRS              8         40
## 2 OXFORD           18         40
```

```
print(observations_by_object)
```

```
## # A tibble: 4 × 4
##   Object     Total_Observations Zero_Bites Some_Bites
##   <fct>                   <int>      <int>      <int>
## 1 Coral                      20          6         14
## 2 Pickleball                 20          7         13
## 3 Seaweed                    20          4         16
## 4 Lego Stack                 20          9         11
```

Test if the difference in the number of trials between groups is
statistically significant using a chi-square test.

```
removed <- removed_by_group$Rows_Removed
total <- removed_by_group$Total_Rows
names(removed) <- removed_by_group$Group  # label groups in output

# Run the test
prop.test(removed, total)
```

```
## 
##  2-sample test for equality of proportions with continuity correction
## 
## data:  removed out of total
## X-squared = 4.6154, df = 1, p-value = 0.03169
## alternative hypothesis: two.sided
## 95 percent confidence interval:
##  -0.47282533 -0.02717467
## sample estimates:
## prop 1 prop 2 
##   0.20   0.45
```

## 4.2 Visualize Latency to Bite Distribution with Histogram

```
ggplot(total_bites_no_zeros, aes(x = Latency_to_Bite, fill = Group)) +
  geom_histogram(position = "stack", bins = 15, color = "black", alpha = 0.8) +
  scale_fill_manual(values = c("OXFORD" = "#002147", "LIRS" = "#C44E52")) +
  labs(
    title = "Stacked Histogram of Latency to Bite by Fish Group",
    x = "Latency to First Bite (seconds)",
    y = "Frequency",
    fill = "Fish Group"
  ) +
  theme_minimal(base_size = 14) +
  theme(
    plot.title = element_text(hjust = 0.5, face = "bold"),
    legend.position = "top",
    axis.text = element_text(size = 12)
  )
```

## 4.3 Testing Distributional Assumptions for GLMMs of Latency to Bite

This section compares generalized linear mixed models (GLMMs) to
identify the most appropriate distribution for modeling latency to bite,
a continuous, positive, and right-skewed time variable. While zero
inflation was not expected, overdispersion due to individual and
object-level variability motivated the evaluation of several model
families.

The Negative Binomial GLMM, although traditionally used for count
data, was included because it can flexibly accommodate overdispersed,
right-skewed responses and sometimes performs well with continuous
outcomes. However, since latency is inherently continuous, models from
the Gaussian and Gamma families were also tested. Model fit is compared
using the Akaike Information Criterion (AIC).

```
# Standard Poisson GLMM (assumes mean = variance)
glmm_pois_L<- glmmTMB(Latency_to_Bite ~ Group + Object + (1 | Fish), family = poisson, data = total_bites_no_zeros)
```

```
## Warning in glmmTMB(Latency_to_Bite ~ Group + Object + (1 | Fish), family =
## poisson, : non-integer counts in a poisson model
```

```
# Negative Binomial GLMM (accounts for overdispersion)
glmm_nb_L <- glmmTMB(Latency_to_Bite ~ Group * Object + (1 | Fish), data = total_bites_no_zeros, family = nbinom2)
```

```
## Warning in glmmTMB(Latency_to_Bite ~ Group * Object + (1 | Fish), data =
## total_bites_no_zeros, : non-integer counts in a nbinom2 model
```

```
# Gaussian GLMM (assumes normally distributed residuals; may struggle with right-skewed data)
glmm_gaussian_L <- glmmTMB(Latency_to_Bite ~ Group * Object + (1 | Fish), data = total_bites_no_zeros, family = gaussian)

# Gamma GLMM with log link (good for continuous, positive, and skewed data)
glmm_gamma_L <- glmmTMB(Latency_to_Bite ~ Group * Object + (1 | Fish), data = total_bites_no_zeros, family = Gamma(link = "log"))

# Compare models using AIC (lower is better)
AIC(glmm_pois_L, glmm_nb_L, glmm_gaussian_L, glmm_gamma_L)
```

```
##                 df       AIC
## glmm_pois_L      6 3583.8736
## glmm_nb_L       10  565.9599
## glmm_gaussian_L 10  674.4941
## glmm_gamma_L    10  564.0011
```

## 4.4 Assessing Model Fit Using Simulated Residuals

We evaluated the adequacy of the selected model using the DHARMa
package. Simulated residuals are used to test for key assumption
violations—including overdispersion, zero-inflation, and non-random
residual structure—through diagnostic plots and statistical tests.

```
# Simulate residuals for diagnostic tests
simulation_output_L1 <- simulateResiduals(fittedModel = glmm_gamma_L, n = 1000)

# Plot residuals: expected vs observed, QQ plot, and residual vs predictor diagnostics
# Look for patterns, skew, or deviations from uniformity
plot(simulation_output_L1)
```

## 4.5 Assessing the Effect of Object Identity on Latency to Bite

Objective: This section tests whether including `Object`
as a predictor improves model fit — either as: a) An interaction with
`Group` (i.e., does object preference depend on whether a
fish is wild or captive?), or  
b) A main effect independent of group (i.e., are some objects more
likely to be bitten quickly regardless of group?).

```
# Model 1: Baseline model with only Group (LIRS vs OXFORD)
# This serves as the simplest model to compare against.
model_no_object_L <- glmmTMB(Latency_to_Bite ~ Group + (1 | Fish), data = total_bites_no_zeros,family = Gamma(link = "log"))

# Model 2: Full model with Group, Object, and their interaction
# Tests whether object identity affects biting, and whether that effect differs by group.
model_with_object_L <- glmmTMB(Latency_to_Bite ~ Group * Object + (1 | Fish), data = total_bites_no_zeros, family = Gamma(link = "log"))

# Model 3: Intermediate model with only Group and Object (no interaction)
# Tests whether objects differ in biting rate, independent of group.
model_object_only_L <- glmmTMB(Latency_to_Bite ~ Group + Object + (1 | Fish), data = total_bites_no_zeros,family = Gamma(link = "log"))

AIC(model_no_object_L,model_with_object_L,model_object_only_L)
```

```
##                     df      AIC
## model_no_object_L    4 566.4702
## model_with_object_L 10 564.0011
## model_object_only_L  7 571.3779
```

```
# Likelihood Ratio Test: Full model vs Group-only model
# This tests whether adding both Object and the Group:Object interaction improves fit.
anova(model_no_object_L, model_with_object_L, REML = FALSE)
```

```
## Data: total_bites_no_zeros
## Models:
## model_no_object_L: Latency_to_Bite ~ Group + (1 | Fish), zi=~0, disp=~1
## model_with_object_L: Latency_to_Bite ~ Group * Object + (1 | Fish), zi=~0, disp=~1
##                     Df    AIC    BIC  logLik deviance  Chisq Chi Df Pr(>Chisq)
## model_no_object_L    4 566.47 574.43 -279.24   558.47                         
## model_with_object_L 10 564.00 583.89 -272.00   544.00 14.469      6    0.02481
##                      
## model_no_object_L    
## model_with_object_L *
## ---
## Signif. codes:  0 '***' 0.001 '**' 0.01 '*' 0.05 '.' 0.1 ' ' 1
```

```
# Likelihood Ratio Test: Main-effects model vs Group-only model
# This tests whether Object has a significant main effect, regardless of interaction.
# anova(model_no_object_L, model_object_only_L, REML = FALSE)

# Likelihood Ratio Test: Full model vs Main-effects model
# This directly tests whether the Group × Object interaction significantly improves model fit.
anova(model_object_only_L, model_with_object_L, REML = FALSE)  # <-- SIGNIFICANT interaction if p < 0.05
```

```
## Data: total_bites_no_zeros
## Models:
## model_object_only_L: Latency_to_Bite ~ Group + Object + (1 | Fish), zi=~0, disp=~1
## model_with_object_L: Latency_to_Bite ~ Group * Object + (1 | Fish), zi=~0, disp=~1
##                     Df    AIC    BIC  logLik deviance  Chisq Chi Df Pr(>Chisq)
## model_object_only_L  7 571.38 585.30 -278.69   557.38                         
## model_with_object_L 10 564.00 583.89 -272.00   544.00 13.377      3   0.003889
##                       
## model_object_only_L   
## model_with_object_L **
## ---
## Signif. codes:  0 '***' 0.001 '**' 0.01 '*' 0.05 '.' 0.1 ' ' 1
```

Model comparison using AIC and likelihood ratio tests indicates that
including the Group × Object interaction significantly improves model
fit.

## 4.6 Assessing Model Fit Using Simulated Residuals

We evaluated the adequacy of the selected model using the DHARMa
package. Simulated residuals are used to test for key assumption
violations—including overdispersion, zero-inflation, and non-random
residual structure—through diagnostic plots and statistical tests.

```
# Simulate residuals for diagnostic tests
simulation_output_L <- simulateResiduals(fittedModel = model_with_object_L, n = 1000)

# Plot residuals: expected vs observed, QQ plot, and residual vs predictor diagnostics
# Look for patterns, skew, or deviations from uniformity
plot(simulation_output_L)
```

### Testing for an interaction between Location and Object

```
# Testing for interaction with location and object
drop1(model_with_object_L, test="Chisq" )
```

```
## Single term deletions
## 
## Model:
## Latency_to_Bite ~ Group * Object + (1 | Fish)
##              Df    AIC    LRT Pr(>Chi)   
## <none>          564.00                   
## Group:Object  3 571.38 13.377 0.003889 **
## ---
## Signif. codes:  0 '***' 0.001 '**' 0.01 '*' 0.05 '.' 0.1 ' ' 1
```

```
#Testing for significance of fish id in model
model_with_object_L_no_fish <- glmmTMB(Latency_to_Bite ~ Group * Object, data = total_bites_no_zeros, family = Gamma(link = "log"))

anova(model_with_object_L, model_with_object_L_no_fish)
```

```
## Data: total_bites_no_zeros
## Models:
## model_with_object_L_no_fish: Latency_to_Bite ~ Group * Object, zi=~0, disp=~1
## model_with_object_L: Latency_to_Bite ~ Group * Object + (1 | Fish), zi=~0, disp=~1
##                             Df    AIC    BIC  logLik deviance  Chisq Chi Df
## model_with_object_L_no_fish  9 563.36 581.27 -272.68   545.36              
## model_with_object_L         10 564.00 583.89 -272.00   544.00 1.3634      1
##                             Pr(>Chisq)
## model_with_object_L_no_fish           
## model_with_object_L             0.2429
```

## 4.7 Evaluating Group Differences in Latency to Bite Across Objects

To determine whether Latency to Bite differs between LIRS and OXFORD
fish, we use estimated marginal means (EMMs)—model-adjusted means that
account for other predictors in the model.

Given that the Group × Object interaction was significant, we
estimate group differences within each object type to assess whether
group effects vary across object identities.

The model used: Latency\_to\_Bite ~ Group \* Object + (1 | Fish)

```
# Compute estimated marginal means (EMMs) for each Group, within each Object level
emm_group_by_object_L <- emmeans(model_with_object_L, ~ Group | Object, type = "response", level = 0.95)

# Convert EMM results to a data frame for inspection or plotting
emm_df_L <- as.data.frame(emm_group_by_object_L)

# Perform pairwise comparisons of Group within each Object
pairwise_contrast_L <- contrast(emm_group_by_object_L, method = "pairwise")

# Summarize contrasts and include confidence intervals (set infer = c(TRUE, TRUE))
contrast_df_L <- summary(pairwise_contrast_L, infer = c(TRUE, TRUE)) |> as.data.frame()

# View the resulting contrast estimates with associated uncertainty
contrast_df_L
```

```
## Object = Coral:
##  contrast          ratio        SE  df asymp.LCL asymp.UCL null z.ratio p.value
##  LIRS / OXFORD 0.0942606 0.0611445 Inf 0.0264349  0.336112    1  -3.641  0.0003
## 
## Object = Pickleball:
##  contrast          ratio        SE  df asymp.LCL asymp.UCL null z.ratio p.value
##  LIRS / OXFORD 0.8595827 0.5877990 Inf 0.2250193  3.283640    1  -0.221  0.8249
## 
## Object = Seaweed:
##  contrast          ratio        SE  df asymp.LCL asymp.UCL null z.ratio p.value
##  LIRS / OXFORD 2.3252045 1.4483917 Inf 0.6858667  7.882837    1   1.355  0.1755
## 
## Object = Lego Stack:
##  contrast          ratio        SE  df asymp.LCL asymp.UCL null z.ratio p.value
##  LIRS / OXFORD 0.2832523 0.2205039 Inf 0.0615938  1.302596    1  -1.620  0.1052
## 
## Confidence level used: 0.95 
## Intervals are back-transformed from the log scale 
## Tests are performed on the log scale
```

## 4.8 Estimated Group Differences in Latency to Bite by Object (Ratios & Significance)

This plot visualizes the estimated group differences in Latency to
Bite behavior between Captive and Wild fish for each object type, based
on pairwise comparisons from the emmeans analysis.

Each point represents the estimated Latency ratio (Captive/Wild) for
a given object, and vertical bars show the 95% confidence interval for
that estimate. These ratios are back-transformed from the log scale to
the response scale for interpretability. The underlying model used a log
link function, so group comparisons were conducted on the log scale and
exponentiated to produce interpretable ratios.

The dashed horizontal line at y = 1 represents the null hypothesis of
no difference in latency to bite between groups. Ratios are calculated
as Captive / Wild, where values below 1 indicate that Captive fish
approached and bit the object faster than Wild fish, while values above
1 indicate that Captive fish were slower to bite than Wild fish. A group
difference is considered statistically significant at the 0.05 level if
the confidence interval does not cross 1.

```
ggplot(contrast_df_L, aes(x = Object, y = ratio)) +
  geom_hline(yintercept = 1, linetype = "dashed", color = "gray50") +
  
  geom_point(size = 3, color = "#002147") +
  geom_errorbar(aes(ymin = asymp.LCL, ymax = asymp.UCL), width = 0.2, color = "#002147") +
  
  labs(
    title = "Ratio of Estimated Latency (Captive / Wild) by Object",
    x = "Object",
    y = "Estimated Ratio ± 95% CI (on response scale)"
  ) +
  theme_minimal(base_size = 14) +
  theme(
    plot.title = element_text(hjust = 0.5, face = "bold"),
    axis.text.x = element_text(angle = 0, hjust = 0.5)
  )
```

## 4.9 Plot B: Estimated Latency to Bite by Group and Object (with Raw Data Overlay)

This plot displays both raw data and model-derived estimates of
Latency to Bite across object types, separated by fish group (Captive
vs. Wild).

- The **faint jittered points** represent individual
  fish-level data for latency, allowing visualization of the underlying
  distribution and variability within each object.
- The **larger colored points** show the estimated
  marginal means (EMMs) for each group within each object, derived from
  the fitted model.
- The **vertical bars** represent the 95% confidence
  intervals for these estimated means.
- Colors distinguish between Captive and Wild fish, and horizontal
  x-axis labels identify the object types.

```
# Label levels for plotting
object_levels <- c("Coral", "Pickleball", "Seaweed", "Lego Stack")
group_levels <- c("OXFORD", "LIRS")

# Filter out NA latency and ensure factor levels are properly set
latency_data_clean <- total_bites_data %>%
  filter(!is.na(Latency_to_Bite)) %>%
  mutate(
    Object = factor(Object, levels = object_levels),
    Group = factor(Group, levels = group_levels))

# Clean model estimates (just in case they were altered)
emm_df_L_clean <- emm_df_L %>%
  mutate(
    Object = factor(as.character(Object), levels = object_levels),
    Group = factor(Group, levels = group_levels)
  ) %>%
  filter(
    !is.na(response),
    !is.na(asymp.LCL),
    !is.na(asymp.UCL),
    is.finite(response),
    is.finite(asymp.LCL),
    is.finite(asymp.UCL))

# Build the full plot
p_latency <- ggplot() +
  # Raw data jitter layer
  geom_jitter(data = latency_data_clean,
              aes(x = Object, y = Latency_to_Bite, color = Group),
              width = 0.15, alpha = 0.3, size = 2, show.legend = FALSE) +

  # Model estimate points
  geom_point(data = emm_df_L_clean,
             aes(x = Object, y = response, color = Group),
             position = position_dodge(width = 0.5),
             size = 3, show.legend = FALSE) +

  # Confidence interval error bars
  geom_errorbar(data = emm_df_L_clean,
                aes(x = Object, ymin = asymp.LCL, ymax = asymp.UCL, color = Group),
                position = position_dodge(width = 0.5),
                width = 0.2, show.legend = FALSE) +

#  scale_color_manual(values = c("OXFORD" = "#002147", "LIRS" = "#C44E52")) +

  # Labels and formatting
  labs(title = "Estimated Latency to First Bite by Group and Object", x = NULL, y = "Latency to bite (seconds)",
       color = "Fish Group") +
  theme_minimal(base_size = 14) +
  theme(plot.title = element_text(face = "bold", hjust = 0.5),
    axis.text.x = element_text(angle = 0, hjust = 0.5),
    axis.line = element_line(color = "black", linewidth = 0.5),
    axis.ticks = element_line(color = "black"))

# Display the plot
p_latency
```

## 4.10 Multiple Comparisons Adjustment Using `multcomp`

To evaluate the significance of group differences within each object,
we first calculated pairwise contrasts using `emmeans`.
However, because we are conducting multiple comparisons (one for each
object), the risk of Type I error increases.

To correct for this, we use the `multcomp` package’s
general linear hypothesis testing (`glht`) framework to
adjust p-values across all group comparisons. This ensures that any
reported significance reflects a more conservative, statistically
rigorous interpretation.

```
# Generate pairwise contrasts: Captive vs Wild within each Object
emm_group_object_L2 <- emmeans(model_with_object_L, pairwise ~ Group | Object)

# Convert emmeans contrast results to a glht object for simultaneous inference
glht_obj_L <- as.glht(emm_group_object_L2$contrasts)

# Summarize the results: estimates, SEs, z-values, and adjusted p-values
# Adjustments control the family-wise error rate (single-step method by default)
# summary(glht_obj_L)
```

Back-Transform results. The back-transformed results are in the
column Ratio and CI\_Lower, CI\_Upper

```
# Z critical value for 95% CI
z_crit <- qnorm(0.975)

# Extract summary stats and compute back-transformed values
glht_summary_table_lat <- map_dfr(names(glht_obj_L), function(obj_name) {
  s <- summary(glht_obj_L[[obj_name]])  # run summary on each glht object
  
  est <- s$test$coefficients
  se <- s$test$sigma
  z <- s$test$tstat
  p <- s$test$pvalues
  
  # Back-transform to response scale (exp for log link)
  ratio <- exp(est)
  ci_lower <- exp(est - z_crit * se)
  ci_upper <- exp(est + z_crit * se)
  
  tibble(
    Object = gsub("Object = ", "", obj_name),
    Log_Estimate = est,
    Std_Error = se, # should not be back-transformed
    z_value = z, # should not be back-transformed
    p_value = p,
    Ratio = ratio,
    CI_Lower = ci_lower,
    CI_Upper = ci_upper
  )
})

# View the result
glht_summary_table_lat
```

```
## # A tibble: 4 × 8
##   Object     Log_Estimate Std_Error z_value  p_value  Ratio CI_Lower CI_Upper
##   <chr>             <dbl>     <dbl>   <dbl>    <dbl>  <dbl>    <dbl>    <dbl>
## 1 Coral            -2.36      0.649  -3.64  0.000272 0.0943   0.0264    0.336
## 2 Pickleball       -0.151     0.684  -0.221 0.825    0.860    0.225     3.28 
## 3 Seaweed           0.844     0.623   1.35  0.176    2.33     0.686     7.88 
## 4 Lego Stack       -1.26      0.778  -1.62  0.105    0.283    0.0616    1.30
```

# 5 Total Time Spent Hiding

Note that we go back to using the full data set, including the trials
when the fish did not bite the objects.

## 5.1 Visualize Hiding Time Distribution with Histogram

```
ggplot(total_bites_data, aes(x = HidingTime, fill = Group)) +
  geom_histogram(position = "stack", bins = 15, color = "black", alpha = 0.8) +
  scale_fill_manual(values = c("OXFORD" = "#002147", "LIRS" = "#C44E52")) +
  labs(
    title = "Stacked Histogram of Hiding Time by Fish Group",
    x = "Total Hiding Time",
    y = "Frequency",
    fill = "Fish Group"
  ) +
  theme_minimal(base_size = 14) +
  theme(
    plot.title = element_text(hjust = 0.5, face = "bold"),
    legend.position = "top",
    axis.text = element_text(size = 12)
  )
```

## 5.2 Testing Distributional Assumptions for GLMMs of Hiding Time

Hiding Time is a continuous, positive, and right-skewed time
variable. While zero inflation was not expected, overdispersion due to
individual and object-level variability motivated the evaluation of
several model families.

The Negative Binomial distribution, although traditionally used for
count data, was included because it can flexibly accommodate
overdispersed, right-skewed responses and sometimes performs well with
continuous outcomes. Since time is inherently continuous, models from
the Gaussian and Gamma families were also tested. Model fit is compared
using the Akaike Information Criterion (AIC).

Gamma distributions cannot accommodate zero or negative values, but 6
out of 80 values (7.5%) in the HidingTime variable equal zero. To
address this, a small constant (+0.01) is added to all values, ensuring
they fall within the valid range for the Gamma family while preserving
their relative differences.

```
# Check proportion of exact zeros in the original data
sum(total_bites_data$HidingTime == 0) / nrow(total_bites_data) * 100
```

```
## [1] 7.5
```

```
# Create a new column with a small offset to make all values strictly positive
hiding_time_data <- total_bites_data %>%
  mutate(HidingTime_adj = HidingTime + 0.01)  # small epsilon

# Confirm that the adjusted values are now all > 0
sum(hiding_time_data$HidingTime_adj == 0) / nrow(hiding_time_data) * 100
```

```
## [1] 0
```

```
# Standard Poisson GLMM (assumes mean = variance)
glmm_pois_H<- glmmTMB(HidingTime_adj ~ Group + Object + (1 | Fish), family = poisson, data = hiding_time_data)
```

```
## Warning in glmmTMB(HidingTime_adj ~ Group + Object + (1 | Fish), family =
## poisson, : non-integer counts in a poisson model
```

```
# Negative Binomial GLMM (accounts for overdispersion)
glmm_nb_H <- glmmTMB(HidingTime_adj ~ Group * Object + (1 | Fish), data = hiding_time_data, family = nbinom2)
```

```
## Warning in glmmTMB(HidingTime_adj ~ Group * Object + (1 | Fish), data =
## hiding_time_data, : non-integer counts in a nbinom2 model
```

```
# Gaussian GLMM (assumes normally distributed residuals; may struggle with right-skewed data)
glmm_gaussian_H <- glmmTMB(HidingTime_adj ~ Group * Object + (1 | Fish), data = hiding_time_data, family = gaussian)

# Gamma GLMM with log link (good for continuous, positive, and skewed data)
glmm_gamma_H <- glmmTMB(HidingTime_adj ~ Group * Object + (1 | Fish), data = hiding_time_data, family = Gamma(link = "log"))

# Compare models using AIC (lower is better)
AIC(glmm_pois_H, glmm_nb_H, glmm_gaussian_H, glmm_gamma_H)
```

```
##                 df       AIC
## glmm_pois_H      6 4207.6066
## glmm_nb_H       10  925.9229
## glmm_gaussian_H 10 1031.0884
## glmm_gamma_H    10  919.2227
```

Model ‘glmm\_gamma\_H’ has the lowest AIC score.

## 5.3 Assessing Model Fit Using Simulated Residuals

We evaluated the adequacy of the selected model using the DHARMa
package.

The ‘glmm\_nb\_H’ and ‘glmm\_gamma\_H’ had similar AIC values. Here we
compare the model fit for both.

```
# Simulate residuals for diagnostic tests
simulation_output_H_nb <- simulateResiduals(fittedModel = glmm_nb_H, n = 1000)
simulation_output_H_gamma <- simulateResiduals(fittedModel = glmm_gamma_H, n = 1000)

# Plot residuals: expected vs observed, QQ plot, and residual vs predictor diagnostics
# Look for patterns, skew, or deviations from uniformity
plot(simulation_output_H_nb)
```

```
plot(simulation_output_H_gamma)
```

Although the Negative Binomial distribution is traditionally used for
count data, we selected a Negative Binomial GLMM (nbinom2 family) to
model hiding time due to its strong empirical fit. Among the candidate
models—including Poisson, Gaussian, and Gamma families, the Negative
Binomial model had the best results in both Akaike Information Criterion
(AIC) and residual diagnostics using DHARMa, with no indication of
overdispersion, zero-inflation, or distributional misfit.

Importantly, while the Gamma model yielded a slightly lower AIC, it
exhibited quantile deviations in residuals, suggesting some
distributional mismatch. In contrast, the Negative Binomial model
provided a robust fit without such violations.

Despite being discrete in its theoretical formulation, the glmmTMB
implementation of the Negative Binomial allows for non-integer values,
and the distribution’s flexibility in modelling right-skewed,
overdispersed data makes it a pragmatic and statistically defensible
choice for behavioural latency measures, where timing often varies
widely across individuals and contexts.

## 5.4 Assessing the Effect of Object Identity on Hiding Time

Objective: This section tests whether including `Object`
as a predictor improves model fit — either as: a) An interaction with
`Group` (i.e., does object preference depend on whether a
fish is wild or captive?), or  
b) A main effect independent of group (i.e., are some objects more
likely to cause longer hiding times regardless of group?).

```
# Model 1: Baseline model with only Group (Captive vs Wild)
# This serves as the simplest model to compare against.
model_no_object_H <- glmmTMB(HidingTime_adj ~ Group + (1 | Fish), family = nbinom2, data = hiding_time_data)
```

```
## Warning in glmmTMB(HidingTime_adj ~ Group + (1 | Fish), family = nbinom2, :
## non-integer counts in a nbinom2 model
```

```
# Model 2: Full model with Group, Object, and their interaction
# Tests whether object identity affects biting, and whether that effect differs by group.
model_with_object_H <- glmmTMB(HidingTime_adj ~ Group * Object + (1 | Fish), family = nbinom2, data = hiding_time_data)
```

```
## Warning in glmmTMB(HidingTime_adj ~ Group * Object + (1 | Fish), family =
## nbinom2, : non-integer counts in a nbinom2 model
```

```
# Model 3: Intermediate model with only Group and Object (no interaction)
# Tests whether objects differ in biting rate, independent of group.
model_object_only_H <- glmmTMB(HidingTime_adj ~ Group + Object + (1 | Fish), family = nbinom2, data = hiding_time_data)
```

```
## Warning in glmmTMB(HidingTime_adj ~ Group + Object + (1 | Fish), family =
## nbinom2, : non-integer counts in a nbinom2 model
```

```
AIC(model_no_object_H,model_with_object_H,model_object_only_H)
```

```
##                     df      AIC
## model_no_object_H    4 925.9399
## model_with_object_H 10 925.9229
## model_object_only_H  7 926.5646
```

```
# Likelihood Ratio Test: Full model vs Group-only model
# This tests whether adding both Object and the Group:Object interaction improves fit.
anova(model_no_object_H, model_with_object_H, REML = FALSE)
```

```
## Data: hiding_time_data
## Models:
## model_no_object_H: HidingTime_adj ~ Group + (1 | Fish), zi=~0, disp=~1
## model_with_object_H: HidingTime_adj ~ Group * Object + (1 | Fish), zi=~0, disp=~1
##                     Df    AIC    BIC  logLik deviance  Chisq Chi Df Pr(>Chisq)
## model_no_object_H    4 925.94 935.47 -458.97   917.94                         
## model_with_object_H 10 925.92 949.74 -452.96   905.92 12.017      6    0.06159
##                      
## model_no_object_H    
## model_with_object_H .
## ---
## Signif. codes:  0 '***' 0.001 '**' 0.01 '*' 0.05 '.' 0.1 ' ' 1
```

```
# Likelihood Ratio Test: Main-effects model vs Group-only model
# This tests whether Object has a significant main effect, regardless of interaction.
#anova(model_no_object_H, model_object_only_H, REML = FALSE)

# Likelihood Ratio Test: Full model vs Main-effects model
# This directly tests whether the Group × Object interaction significantly improves model fit.
anova(model_object_only_H, model_with_object_H, REML = FALSE)  # <-- SIGNIFICANT interaction if p < 0.05
```

```
## Data: hiding_time_data
## Models:
## model_object_only_H: HidingTime_adj ~ Group + Object + (1 | Fish), zi=~0, disp=~1
## model_with_object_H: HidingTime_adj ~ Group * Object + (1 | Fish), zi=~0, disp=~1
##                     Df    AIC    BIC  logLik deviance  Chisq Chi Df Pr(>Chisq)
## model_object_only_H  7 926.56 943.24 -456.28   912.56                         
## model_with_object_H 10 925.92 949.74 -452.96   905.92 6.6418      3    0.08424
##                      
## model_object_only_H  
## model_with_object_H .
## ---
## Signif. codes:  0 '***' 0.001 '**' 0.01 '*' 0.05 '.' 0.1 ' ' 1
```

Model comparison using AIC show that the three models are very
similar. The likelihood ratio tests indicates that the models are not
significantly different.

## 5.5 Assessing Model Fit Using Simulated Residuals

All three models are very similar in terms of AIC values and there is
no statistically significance in the models. Here we compare the model
fit to check if any models fit better than others.

```
# Simulate residuals for diagnostic tests
simulation_output_H_no_object <- simulateResiduals(fittedModel = model_no_object_H, n = 1000)
simulation_output_H_object_only <- simulateResiduals(fittedModel = model_object_only_H, n = 1000)
simulation_output_H_with_object <- simulateResiduals(fittedModel = model_with_object_H, n = 1000)

# Plot residuals: expected vs observed, QQ plot, and residual vs predictor diagnostics
# Look for patterns, skew, or deviations from uniformity
plot(simulation_output_H_no_object)
```

```
plot(simulation_output_H_object_only)
```

```
plot(simulation_output_H_with_object)
```

The model excluding Object violated the DHARMa test for homogeneity of
variance, indicating poor model fit. In contrast, both models that
included Object—with and without the interaction term—passed all DHARMa
diagnostic tests. To maintain consistency with previous analyses and to
account for potential object-specific variation in hiding behavior, we
elected to proceed with the model including Object as a fixed
effect.

## 5.6 Evaluating Group Differences in Hiding Time Across Objects

To determine whether Hiding Time differs between wild and captive
fish, we use estimated marginal means (EMMs)—model-adjusted means that
account for other predictors in the model. This allows for a fair
comparison of Group effects.

The model used: HidingTime\_adj ~ Group \* Object + (1 | Fish)

```
# Compute estimated marginal means (EMMs) for each Group, within each Object level
emm_group_by_object_H <- emmeans(model_with_object_H, ~ Group | Object, type = "response", level = 0.95)

# Convert EMM results to a data frame for inspection or plotting
emm_df_H <- as.data.frame(emm_group_by_object_H)

# Perform pairwise comparisons of Group within each Object
pairwise_contrast_H <- contrast(emm_group_by_object_H, method = "pairwise")

# Summarize contrasts and include confidence intervals (set infer = c(TRUE, TRUE))
contrast_df_H <- summary(pairwise_contrast_H, infer = c(TRUE, TRUE)) |> as.data.frame()

# View the resulting contrast estimates with associated uncertainty
contrast_df_H
```

```
## Object = Coral:
##  contrast         ratio       SE  df asymp.LCL asymp.UCL null z.ratio p.value
##  LIRS / OXFORD 1.636466 1.217347 Inf 0.3808102   7.03243    1   0.662  0.5079
## 
## Object = Pickleball:
##  contrast         ratio       SE  df asymp.LCL asymp.UCL null z.ratio p.value
##  LIRS / OXFORD 4.958751 3.718235 Inf 1.1405475  21.55913    1   2.135  0.0327
## 
## Object = Seaweed:
##  contrast         ratio       SE  df asymp.LCL asymp.UCL null z.ratio p.value
##  LIRS / OXFORD 3.420913 2.570819 Inf 0.7842661  14.92178    1   1.637  0.1017
## 
## Object = Lego Stack:
##  contrast         ratio       SE  df asymp.LCL asymp.UCL null z.ratio p.value
##  LIRS / OXFORD 1.148626 0.858810 Inf 0.2653067   4.97289    1   0.185  0.8530
## 
## Confidence level used: 0.95 
## Intervals are back-transformed from the log scale 
## Tests are performed on the log scale
```

Note: The ratios represent LIRS / OXFORD, so values > 1 mean
higher time at LIRS.

### 5.6.1 Testing for an interaction between Object and Location

```
# Testing for interaction with location and object
drop1(model_with_object_H, test="Chisq" )
```

```
## Warning in glmmTMB(formula = HidingTime_adj ~ Group + Object + (1 | Fish), :
## non-integer counts in a nbinom2 model
```

```
## Single term deletions
## 
## Model:
## HidingTime_adj ~ Group * Object + (1 | Fish)
##              Df    AIC    LRT Pr(>Chi)  
## <none>          925.92                  
## Group:Object  3 926.56 6.6418  0.08424 .
## ---
## Signif. codes:  0 '***' 0.001 '**' 0.01 '*' 0.05 '.' 0.1 ' ' 1
```

```
#Testing for significance of fish id in model
model_with_object_H_no_fish <- glmmTMB(HidingTime_adj ~ Group * Object, family = nbinom2, data = hiding_time_data)
```

```
## Warning in glmmTMB(HidingTime_adj ~ Group * Object, family = nbinom2, data =
## hiding_time_data): non-integer counts in a nbinom2 model
```

```
anova(model_with_object_H, model_with_object_H_no_fish)
```

```
## Data: hiding_time_data
## Models:
## model_with_object_H_no_fish: HidingTime_adj ~ Group * Object, zi=~0, disp=~1
## model_with_object_H: HidingTime_adj ~ Group * Object + (1 | Fish), zi=~0, disp=~1
##                             Df    AIC    BIC  logLik deviance  Chisq Chi Df
## model_with_object_H_no_fish  9 962.10 983.54 -472.05   944.10              
## model_with_object_H         10 925.92 949.74 -452.96   905.92 38.181      1
##                             Pr(>Chisq)    
## model_with_object_H_no_fish               
## model_with_object_H          6.447e-10 ***
## ---
## Signif. codes:  0 '***' 0.001 '**' 0.01 '*' 0.05 '.' 0.1 ' ' 1
```

## 5.7 Estimated Group Differences in Hiding Time by Object (Ratios & Significance)

This plot visualizes the estimated group differences in Hiding Time
between Captive and Wild fish for each object type, based on pairwise
comparisons from the emmeans analysis.

Each point represents the estimated Hiding Time ratio (Captive/Wild)
for a given object, and vertical bars show the 95% confidence interval
for that estimate. These ratios are back-transformed from the log scale
to the response scale for interpretability. The underlying model used a
log link function, so group comparisons were conducted on the log scale
and exponentiated to produce interpretable ratios.

The dashed horizontal line at y = 1 represents the null hypothesis of
no difference in Hiding Time between groups. Ratios are calculated as
Captive / Wild, where values below 1 indicate that Captive fish hid from
the object longer than Wild fish, while values above 1 indicate that
Captive fish hid less than Wild fish. A group difference is considered
statistically significant at the 0.05 level if the confidence interval
does not cross 1.

```
ggplot(contrast_df_H, aes(x = Object, y = ratio)) +
  geom_hline(yintercept = 1, linetype = "dashed", color = "gray50") +
  
  geom_point(size = 3, color = "#002147") +
  geom_errorbar(aes(ymin = asymp.LCL, ymax = asymp.UCL), width = 0.2, color = "#002147") +
  
  labs(
    title = "Ratio of Estimated Hiding Time (Captive / Wild) by Object",
    x = "Object",
    y = "Estimated Ratio ± 95% CI (on response scale)"
  ) +
  theme_minimal(base_size = 14) +
  theme(
    plot.title = element_text(hjust = 0.5, face = "bold"),
    axis.text.x = element_text(angle = 0, hjust = 0.5)
  )
```

## 5.8 Plot C: Estimated Hiding Time by Group and Object (with Raw Data Overlay)

This plot displays both raw data and model-derived estimates of
Hiding Time across object types, separated by fish group (Captive
vs. Wild).

- The **faint jittered points** represent individual
  fish-level data, allowing visualization of the underlying distribution
  and variability within each object.
- The **larger colored points** show the estimated
  marginal means (EMMs) for each group within each object, derived from
  the fitted model.
- The **vertical bars** represent the 95% confidence
  intervals for these estimated means.
- Colors distinguish between Captive and Wild fish, and horizontal
  x-axis labels identify the object types.

```
# Label levels for plotting
object_levels <- c("Coral", "Pickleball", "Seaweed", "Lego Stack")
hiding_time_data$Object     <- factor(hiding_time_data$Object,     levels = object_levels)
emm_df_H$Object             <- factor(emm_df_H$Object,             levels = object_levels)
hiding_time_data$Group     <- factor(hiding_time_data$Group,     levels = group_levels)
emm_df_H$Group             <- factor(emm_df_H$Group,             levels = group_levels)

p_hiding <- ggplot() +
  # Raw data layer
  geom_jitter(data = hiding_time_data,
              aes(x = Object, y = HidingTime_adj, color = Group),
              alpha = 0.3, size = 2, width = 0.15, show.legend = FALSE) +

  # EMM points (mean estimates)
  geom_point(data = emm_df_H,
             aes(x = Object, y = response, color = Group),
             position = position_dodge(width = 0.5), size = 3, show.legend = FALSE) +

  # CI error bars (use asymp.LCL and asymp.UCL from emmeans output)
  geom_errorbar(data = emm_df_H,
                aes(x = Object, ymin = asymp.LCL, ymax = asymp.UCL, color = Group),
                position = position_dodge(width = 0.5), width = 0.2, show.legend = FALSE) +

#  scale_color_manual(values = c("OXFORD" = "#002147", "LIRS" = "#C44E52"), drop = FALSE) +

  # Labels
  labs(
    title = "Estimated Hiding Time by Group and Object",
    x = NULL,
    y = "Hiding time (seconds)",
    color = "Fish Group"
  ) +

  # Theme adjustments
  theme_minimal(base_size = 14) +
  theme(
    plot.title = element_text(hjust = 0.5, face = "bold"),
    axis.text.x = element_text(angle = 0, hjust = 0.5),  # Horizontal labels
#    legend.position = "top",
    axis.line = element_line(color = "black", linewidth = 0.5),
    axis.ticks = element_line(color = "black")
  )

p_hiding
```

## 5.9 Multiple Comparisons Adjustment Using `multcomp`

To evaluate the significance of group differences within each object,
we first calculated pairwise contrasts using `emmeans`.
However, because we are conducting multiple comparisons (one for each
object), the risk of Type I error increases.

To correct for this, we use the `multcomp` package’s
general linear hypothesis testing (`glht`) framework to
adjust p-values across all group comparisons. This ensures that any
reported significance reflects a more conservative, statistically
rigorous interpretation.

```
# Generate pairwise contrasts: OXFORD vs LIRS within each Object
emm_group_object_H2 <- emmeans(model_with_object_H, pairwise ~ Group | Object)

# Convert emmeans contrast results to a glht object for simultaneous inference
glht_obj_H <- as.glht(emm_group_object_H2$contrasts)

# Summarize the results: estimates, SEs, z-values, and adjusted p-values
# Adjustments control the family-wise error rate (single-step method by default)
summary(glht_obj_H)
```

```
## $`Object = Coral`
## 
##   Simultaneous Tests for General Linear Hypotheses
## 
## Linear Hypotheses:
##                    Estimate Std. Error z value Pr(>|z|)
## OXFORD - LIRS == 0   0.4925     0.7439   0.662    0.508
## (Adjusted p values reported -- single-step method)
## 
## 
## $`Object = Pickleball`
## 
##   Simultaneous Tests for General Linear Hypotheses
## 
## Linear Hypotheses:
##                    Estimate Std. Error z value Pr(>|z|)  
## OXFORD - LIRS == 0   1.6012     0.7498   2.135   0.0327 *
## ---
## Signif. codes:  0 '***' 0.001 '**' 0.01 '*' 0.05 '.' 0.1 ' ' 1
## (Adjusted p values reported -- single-step method)
## 
## 
## $`Object = Seaweed`
## 
##   Simultaneous Tests for General Linear Hypotheses
## 
## Linear Hypotheses:
##                    Estimate Std. Error z value Pr(>|z|)
## OXFORD - LIRS == 0   1.2299     0.7515   1.637    0.102
## (Adjusted p values reported -- single-step method)
## 
## 
## $`Object = Lego Stack`
## 
##   Simultaneous Tests for General Linear Hypotheses
## 
## Linear Hypotheses:
##                    Estimate Std. Error z value Pr(>|z|)
## OXFORD - LIRS == 0   0.1386     0.7477   0.185    0.853
## (Adjusted p values reported -- single-step method)
```

Back-Transform results. The back-transformed results are in the
column Ratio and CI\_Lower, CI\_Upper

```
# Z critical value for 95% CI
z_crit <- qnorm(0.975)

# Extract summary stats and compute back-transformed values
glht_summary_table_hid <- map_dfr(names(glht_obj_H), function(obj_name) {
  s <- summary(glht_obj_H[[obj_name]])  # run summary on each glht object
  
  est <- s$test$coefficients
  se <- s$test$sigma
  z <- s$test$tstat
  p <- s$test$pvalues
  
  # Back-transform to response scale (exp for log link)
  ratio <- exp(est)
  ci_lower <- exp(est - z_crit * se)
  ci_upper <- exp(est + z_crit * se)
  
  tibble(
    Object = gsub("Object = ", "", obj_name),
    Log_Estimate = est,
    Std_Error = se, # should not be back-transformed
    z_value = z, # should not be back-transformed
    p_value = p,
    Ratio = ratio,
    CI_Lower = ci_lower,
    CI_Upper = ci_upper
  )
})

# View the result
glht_summary_table_hid
```

```
## # A tibble: 4 × 8
##   Object     Log_Estimate Std_Error z_value p_value Ratio CI_Lower CI_Upper
##   <chr>             <dbl>     <dbl>   <dbl>   <dbl> <dbl>    <dbl>    <dbl>
## 1 Coral             0.493     0.744   0.662  0.508   1.64    0.381     7.03
## 2 Pickleball        1.60      0.750   2.14   0.0327  4.96    1.14     21.6 
## 3 Seaweed           1.23      0.752   1.64   0.102   3.42    0.784    14.9 
## 4 Lego Stack        0.139     0.748   0.185  0.853   1.15    0.265     4.97
```

# 6 Make a combined plot of all results

```
p_bites <- p_bites + scale_x_discrete(labels = c(
  "Coral" = "Coral",
  "Pickleball" = "Pickleball",
  "Seaweed" = "Seaweed",
  "Lego Stack" = "Lego"))

p_latency <- p_latency + scale_x_discrete(labels = c(
  "Coral" = "Coral",
  "Pickleball" = "Pickleball",
  "Seaweed" = "Seaweed",
  "Lego Stack" = "Lego"))

p_hiding <- p_hiding + scale_x_discrete(labels = c(
  "Coral" = "Coral",
  "Pickleball" = "Pickleball",
  "Seaweed" = "Seaweed",
  "Lego Stack" = "Lego"))

# Define a shared color scale for all plots
color_scale <- scale_color_manual(name="Location",
                                  values = c("OXFORD" = "#002147", "LIRS" = "#C44E52"), drop = FALSE)

# Remove legends from each individual plot
p_bites_clean <- p_bites + ggtitle(NULL) + color_scale
p_latency_clean <- p_latency + ggtitle(NULL) + color_scale
p_hiding_clean <- p_hiding + ggtitle(NULL) + color_scale

combined_plot <- (p_bites_clean | p_latency_clean | p_hiding_clean) +
  plot_layout(guides = "collect") & 
  theme(legend.position = "right",
        plot.margin = margin(40, 10, 10, 10)) # top, right, bottom, left)

combined_plot <- combined_plot + plot_annotation(tag_levels = 'A')

combined_plot
```

Save the plot

```
ggsave("Fig3_Behavioral_Comparisons2.png", combined_plot, 
       width = 14, height = 5.8, dpi = 600, units = "in")
```
