# Supplementary material for: Differential captivity and experiential conditions and its impact on the behaviour and cognition of Picasso triggerfish (Rhinecanthus aculeatus)
Source: Anim Cogn. 2026 Mar 14;29(1):33. doi: 10.1007/s10071-026-02057-1 (PMC13002752; doi:10.1007/s10071-026-02057-1)
Supplement: Supplementary file 6 — Supplementary file6 (HTML 2106 KB) [file 10071_2026_2057_MOESM6_ESM.html]

Puzzle Preference Test Analysis


# Puzzle Preference Test Analysis

#### Cait Newport

#### 2025-06-25

# 1 Project Description

This analysis is part of the data processing pipeline for the
**Puzzle Preference Test**, a behavioral assessment used in
the associated manuscript: *Behavioural Differences Across Captivity
Conditions in Triggerfish (Rhinecanthus aculeatus): Implications for
Cognitive Testing*.

The current notebook focuses on the following components of the
behavioral dataset:

- **Pass Rate**: Number of fish to solve the puzzle
  feeder.
- **Food Choice Order**: Food type selection order (up to
  5 choices per trial).

## 1.1 Set up the Environment

### 1.1.1 Load packages

```
## ── Attaching core tidyverse packages ──────────────────────── tidyverse 2.0.0 ──
## ✔ dplyr     1.1.4     ✔ readr     2.1.5
## ✔ forcats   1.0.0     ✔ stringr   1.5.1
## ✔ ggplot2   3.5.1     ✔ tibble    3.2.1
## ✔ lubridate 1.9.3     ✔ tidyr     1.3.1
## ✔ purrr     1.0.2     
## ── Conflicts ────────────────────────────────────────── tidyverse_conflicts() ──
## ✖ dplyr::filter() masks stats::filter()
## ✖ dplyr::lag()    masks stats::lag()
## ℹ Use the conflicted package (<http://conflicted.r-lib.org/>) to force all conflicts to become errors
```

```
## Warning in checkDepPackageVersion(dep_pkg = "TMB"): Package version inconsistency detected.
## glmmTMB was built with TMB version 1.9.11
## Current TMB version is 1.9.14
## Please re-install glmmTMB from source or restore original 'TMB' package (see '?reinstalling' for more information)
```

```
## Loading required package: Matrix
## 
## Attaching package: 'Matrix'
## 
## The following objects are masked from 'package:tidyr':
## 
##     expand, pack, unpack
## 
## 
## Attaching package: 'lmerTest'
## 
## The following object is masked from 'package:lme4':
## 
##     lmer
## 
## The following object is masked from 'package:stats':
## 
##     step
## 
## This is DHARMa 0.4.6. For overview type '?DHARMa'. For recent changes, type news(package = 'DHARMa')
## 
## Attaching package: 'nlme'
## 
## The following object is masked from 'package:lme4':
## 
##     lmList
## 
## The following object is masked from 'package:dplyr':
## 
##     collapse
## 
## Welcome to emmeans.
## Caution: You lose important information if you filter this package's results.
## See '? untidy'
```

### 1.1.2 Load data

```
# Load the Excel file
setwd("/Users/user/projects/CaptiveCognition")
data_Pretraining <- read_excel("FoodPreferenceTest/FoodPreferenceTest.xlsx", sheet = "training")
data_Test <- read_excel("FoodPreferenceTest/FoodPreferenceTest.xlsx", sheet = "testing")
```

## 1.2 Summarise the data

Fish used in the experiment

```
fish_by_group <- data_Test %>%
  dplyr::select(fish_id, location) %>%
  distinct() %>%
  arrange(location, fish_id) %>%
  group_by(location) %>%
  summarise(Fish_IDs = paste(sort(unique(fish_id)), collapse = ", ")) %>%
  pivot_wider(names_from = location, values_from = Fish_IDs)

print(fish_by_group)
```

```
## # A tibble: 1 × 2
##   LIRS                          OXFORD                                    
##   <chr>                         <chr>                                     
## 1 1, 2, 3, 4, 5, 6, 7, 8, 9, 10 49, 53, 54, 58, 59, 60, 62, 63, 64, 65, 66
```

# 2 PHASE 1: Proportion of fish to pass Phase 1. Puzzle Feeder

```
data_summary_counts <- data_Pretraining %>%
  filter(outcome != "no_interaction") %>% # Exclude 'no_interaction'
  mutate(result = ifelse(outcome == "pass", "Pass", "Fail")) %>%  # Label outcome
  group_by(location, replicate, result) %>%  # Count outcomes
  summarise(count = n(), .groups = "drop")

ggplot(data_summary_counts, aes(x = factor(replicate), y = count, fill = result)) +
  geom_bar(stat = "identity", position = "stack") +
  facet_wrap(~location) +
  labs(
    title = "Number of Fish that Passed or Failed per Replicate by Location",
    x = "Replicate",
    y = "Number of Fish",
    fill = "Outcome"
  ) +
  theme_minimal()
```

```
data_summary_counts %>%
  arrange(location, replicate, result)
```

```
## # A tibble: 12 × 4
##    location replicate result count
##    <chr>        <dbl> <chr>  <int>
##  1 LIRS             1 Fail       4
##  2 LIRS             1 Pass       6
##  3 LIRS             2 Pass      10
##  4 LIRS             3 Pass      10
##  5 LIRS             4 Pass      10
##  6 LIRS             5 Pass      10
##  7 OXFORD           1 Fail       1
##  8 OXFORD           1 Pass      10
##  9 OXFORD           2 Pass      11
## 10 OXFORD           3 Pass      11
## 11 OXFORD           4 Pass      11
## 12 OXFORD           5 Pass      11
```

### 2.0.1 Analysis of pass rate for Replicate 1

Here we test if To determine whether pass/fail rates differ between
locations (LIRS and OXFORD) for Replicate 1. Rep 1 was chosen because it
was the only replicate in the dataset where there was a difference in
pass/fail outcomes between the two locations (LIRS and OXFORD).

A Fisher’s Exact Test was then applied to assess whether the
proportion of pass/fail outcomes differed significantly between
locations. This test is appropriate for small sample sizes and provides
an exact p-value, making it suitable for contingency tables with low
cell counts.

```
# Filter for replicate 1
rep1_data <- data_summary_counts %>%
  filter(replicate == 1)

# Create a contingency table
contingency <- xtabs(count ~ location + result, data = rep1_data)

# Perform Fisher's exact test
fisher.test(contingency)
```

```
## 
##  Fisher's Exact Test for Count Data
## 
## data:  contingency
## p-value = 0.1486
## alternative hypothesis: true odds ratio is not equal to 1
## 95 percent confidence interval:
##    0.4581258 359.4564908
## sample estimates:
## odds ratio 
##   6.080683
```

Our test shows there is no statistically significant difference
between the locations (p = 0.1486).

# 3 PHASE 2: Food Preference Test

## 3.1 Analysis of First Choices

### 3.1.1 Explore data

```
# Build contingency table: rows = locations, columns = food items
choice_table <- data_Test %>%
  dplyr::select(location, choice_1) %>%
  table()
choice_table
```

```
##         choice_1
## location Fish Mussel Pellet Shrimp Squid
##   LIRS     59     47      0     32     9
##   OXFORD   38     63     12     32    20
```

Plot the number of choices by food item.

```
# Create the data frame
choice_table_plot <- data.frame(
  location = c("LIRS", "OXFORD"),
  Fish = c(59, 38),
  Mussel = c(47, 63),
  Pellet = c(0, 12),
  Shrimp = c(32, 32),
  Squid = c(9, 20))

# Convert to long format
choice_long <- choice_table_plot %>%
  pivot_longer(cols = -location, names_to = "food", values_to = "count") %>%
  mutate(food = factor(food, levels = c("Fish", "Mussel", "Shrimp", "Squid", "Pellet")))  # Reorder

# Plot with custom colors and legend title
ggplot(choice_long, aes(x = food, y = count, fill = location)) +
  geom_bar(stat = "identity", position = position_dodge()) +
  scale_fill_manual(values = c("LIRS" = "#C44E52", "OXFORD" = "#002147")) +
  labs(
    x = "Food Type",
    y = "Number of first choices (count)",
    fill = "Location",  # Capital L here
    title = "Food Preferences by Location") +
  theme_minimal()
```

Note that pellet was never chosen at LIRS. For the statistical models
I will be using, this item will have to be dropped as you can’t have
zeros.

```
choice_summary <- data_Test %>%
  filter(!is.na(choice_1)) %>%
  group_by(fish_id, location, food_item = choice_1) %>%
  summarise(count = n(), .groups = "drop") %>%
  arrange(fish_id)

#choice_summary

# Remove instances of pellet
choice_summary_no_pellet <- choice_summary %>% filter(food_item != "Pellet")
```

### 3.1.2 Model Fitting: Choice Count by Location and Food Item

In our model, the Response variable is choice frequencies, so we will
try to make a model that uses a distribution appropriate for count
data.

```
# Note that the pellet food_item has been dropped

# Model using a poisson distribution
model_poisson <- glmmTMB(count ~ location * food_item + (1 | fish_id), family = poisson, data = choice_summary_no_pellet)

# Model using a negative binomial distrubution
model_nb <- glmmTMB(count ~ location * food_item + (1 | fish_id), family = nbinom2(), data = choice_summary_no_pellet)

# Testing for normality of model residuals using the DHARMa package
simulation_output <- simulateResiduals(fittedModel = model_poisson, n = 1000, plot=TRUE)
```

```
simulation_output <- simulateResiduals(fittedModel = model_nb, n = 1000, plot=TRUE)
```

```
AIC(model_poisson, model_nb)
```

```
##               df      AIC
## model_poisson  9 333.1438
## model_nb      10 331.9133
```

Only the model with the negative binomial fits the data well, and it
has a slightly lower AIC value.

### 3.1.3 Testing the Effect of Individual Fish

To assess whether individual fish differ in their first food choice
beyond location and food item effects, we compare models with and
without the (1 | fish\_id) term using a likelihood ratio test and AIC. We
also test if the model without fish meets DHARMa criterion.

```
# Model using a negative binomial distrubution
#model_nb <- glmmTMB(count ~ location * food_item + (1 | fish_id), family = nbinom2(), data = choice_summary_no_pellet)

# model no fish
model_no_fish <- glmmTMB(count ~ location * food_item, family = nbinom2(), data = choice_summary_no_pellet)

# Likelihood ratio test
anova(model_nb, model_no_fish)
```

```
## Data: choice_summary_no_pellet
## Models:
## model_no_fish: count ~ location * food_item, zi=~0, disp=~1
## model_nb: count ~ location * food_item + (1 | fish_id), zi=~0, disp=~1
##               Df    AIC    BIC  logLik deviance Chisq Chi Df Pr(>Chisq)
## model_no_fish  9 329.91 350.53 -155.96   311.91                        
## model_nb      10 331.91 354.82 -155.96   311.91     0      1          1
```

```
drop1(model_no_fish, test="Chisq")
```

```
## Single term deletions
## 
## Model:
## count ~ location * food_item
##                    Df    AIC    LRT Pr(>Chi)
## <none>                329.91                
## location:food_item  3 327.55 3.6378   0.3033
```

```
simulation_output <- simulateResiduals(fittedModel = model_no_fish, n = 1000, plot=TRUE)
```

Individual fish Id does not explain additional variance in food choice
counts after accounting for location and food item. Since the repeated
measures do not different enough to justify inclusion in the model, we
will stick with the simpler model. Residuals have also been checked
using DHARMa.

### 3.1.4 Model Interpretation

```
summary(model_no_fish)
```

```
##  Family: nbinom2  ( log )
## Formula:          count ~ location * food_item
## Data: choice_summary_no_pellet
## 
##      AIC      BIC   logLik deviance df.resid 
##    329.9    350.5   -156.0    311.9       64 
## 
## 
## Dispersion parameter for nbinom2 family (): 13.3 
## 
## Conditional model:
##                                Estimate Std. Error z value Pr(>|z|)    
## (Intercept)                      1.8803     0.1590  11.827  < 2e-16 ***
## locationOXFORD                  -0.6406     0.2417  -2.651  0.00803 ** 
## food_itemMussel                 -0.1096     0.2365  -0.463  0.64302    
## food_itemShrimp                 -0.6118     0.2547  -2.402  0.01629 *  
## food_itemSquid                  -0.7817     0.4017  -1.946  0.05166 .  
## locationOXFORD:food_itemMussel   0.6152     0.3343   1.840  0.06573 .  
## locationOXFORD:food_itemShrimp   0.4399     0.3688   1.193  0.23296    
## locationOXFORD:food_itemSquid    0.1398     0.5013   0.279  0.78027    
## ---
## Signif. codes:  0 '***' 0.001 '**' 0.01 '*' 0.05 '.' 0.1 ' ' 1
```

The intercept for our model is LIRS-Fish. Thus all p-values are in
relation to that intercept.

If we want to compare Oxford-Fish to the other levels, we need to
relevel the terms and run the model again. This model is mathemetically
identical, it just changes the reference value.

```
choice_summary_no_pellet <- choice_summary_no_pellet %>%
  mutate(location = factor(location),
    food_item = factor(food_item)) %>%
  mutate(location = relevel(location, ref = "OXFORD"),
    food_item = relevel(food_item, ref = "Fish"))

model_oxford_fish <- glmmTMB(
  count ~ location * food_item,
  family = nbinom2(),
  data = choice_summary_no_pellet)

summary(model_oxford_fish)
```

```
##  Family: nbinom2  ( log )
## Formula:          count ~ location * food_item
## Data: choice_summary_no_pellet
## 
##      AIC      BIC   logLik deviance df.resid 
##    329.9    350.5   -156.0    311.9       64 
## 
## 
## Dispersion parameter for nbinom2 family (): 13.3 
## 
## Conditional model:
##                              Estimate Std. Error z value Pr(>|z|)    
## (Intercept)                    1.2397     0.1820   6.811  9.7e-12 ***
## locationLIRS                   0.6406     0.2417   2.651  0.00803 ** 
## food_itemMussel                0.5055     0.2363   2.140  0.03237 *  
## food_itemShrimp               -0.1718     0.2668  -0.644  0.51953    
## food_itemSquid                -0.6419     0.2999  -2.140  0.03234 *  
## locationLIRS:food_itemMussel  -0.6152     0.3343  -1.840  0.06573 .  
## locationLIRS:food_itemShrimp  -0.4400     0.3688  -1.193  0.23296    
## locationLIRS:food_itemSquid   -0.1398     0.5013  -0.279  0.78027    
## ---
## Signif. codes:  0 '***' 0.001 '**' 0.01 '*' 0.05 '.' 0.1 ' ' 1
```

### 3.1.5 Calculate Estimated Marginal Means

This is mainly used for plotting

```
# Note that the results get back-transformed
emm <- emmeans(model_no_fish, ~ food_item | location, type = "response")
```

### 3.1.6 Pairwise comparison

Pairwise comparisons of the estimated marginal means were conducted
to evaluate differences between groups (food items by location). This
allows us to identify which group levels differ significantly from one
another.

A Tukey adjustment was applied to control for the increased risk of
Type I error due to multiple comparisons.

```
pairs(emm,adjust="tukey")
```

```
## location = OXFORD:
##  contrast        ratio    SE  df null z.ratio p.value
##  Fish / Mussel   1.116 0.264 Inf    1   0.463  0.9670
##  Fish / Shrimp   1.844 0.470 Inf    1   2.402  0.0766
##  Fish / Squid    2.185 0.878 Inf    1   1.946  0.2089
##  Mussel / Shrimp 1.652 0.438 Inf    1   1.895  0.2301
##  Mussel / Squid  1.958 0.800 Inf    1   1.646  0.3528
##  Shrimp / Squid  1.185 0.497 Inf    1   0.405  0.9775
## 
## location = LIRS:
##  contrast        ratio    SE  df null z.ratio p.value
##  Fish / Mussel   0.603 0.143 Inf    1  -2.140  0.1406
##  Fish / Shrimp   1.188 0.317 Inf    1   0.644  0.9176
##  Fish / Squid    1.900 0.570 Inf    1   2.140  0.1405
##  Mussel / Shrimp 1.969 0.485 Inf    1   2.748  0.0305
##  Mussel / Squid  3.150 0.888 Inf    1   4.069  0.0003
##  Shrimp / Squid  1.600 0.493 Inf    1   1.526  0.4219
## 
## P value adjustment: tukey method for comparing a family of 4 estimates 
## Tests are performed on the log scale
```

### 3.1.7 Plot results

```
emm_df <- as.data.frame(emm)

# Make sure location is a factor with correct order
choice_summary$location <- factor(choice_summary$location, levels = c("OXFORD", "LIRS"))
emm_df$location <- factor(emm_df$location, levels = c("OXFORD", "LIRS"))

# Define positions
pj <- position_jitterdodge(dodge.width = 0.6, jitter.width = 0.2)
pd <- position_dodge(width = 0.6)

# Plot
food_choice <- ggplot(choice_summary, aes(x = food_item, y = count, color = location)) +
  
  # Raw data points with jitter and dodge
  geom_point(aes(group = location), position = pj, size = 1.5, alpha = 0.3) +

  # Model-based means with CI
  geom_errorbar(data = emm_df,
  mapping = aes(x = food_item, y = response, ymin = asymp.LCL, ymax = asymp.UCL, group = location, color = location),
  position = pd, width = 0.15, linewidth = 0.7, inherit.aes = FALSE) +
  
  geom_point(data = emm_df, mapping = aes(x = food_item, y = response, group = location, fill = location),
    position = pd, size = 3, shape = 21, color = "black", inherit.aes = FALSE) +

  # Custom color palette
  scale_color_manual(
    values = c("OXFORD" = "#002147", "LIRS" = "#C44E52"),
    breaks = c("OXFORD", "LIRS"),
    labels = c("OXFORD", "LIRS")) +
  scale_fill_manual(
    values = c("OXFORD" = "#002147", "LIRS" = "#C44E52"),
    breaks = c("OXFORD", "LIRS"),
    labels = c("OXFORD", "LIRS")) +

  # Labels and theme
  labs(x = "Food Type",
    y = "Number of choices",
    color = "Location",
    fill = "Location") +
  
  theme_minimal(base_size = 14) +
  theme(plot.title = element_text(hjust = 0.5, face = "bold"),
    axis.line = element_line(color = "black"),
    axis.ticks = element_line(color = "black"),
    legend.position = "right")

food_choice
```

Save plot

```
ggsave("/Users/user/projects/CaptiveCognition/food_choice_figure.png", plot = food_choice, width = 12, height = 6, dpi = 300)
```

### 3.1.8 Effect of Replicates

This section explores whether replicate number influences first food
choice. Although models were tested, the analysis was not included in
the final paper due to limitations in the dataset.

The data are sparse when grouped by location \* food\_item \* replicate,
with most groups having only 1–3 observations. Additionally, Pellet had
to be removed due to zero counts, and both fixed and random effects
models failed to converge reliably.

```
# Count first-choice selections by replicate, location, and food item
choice_data_by_replicate <- data_Test %>%
  count(replicate, location, food_item = choice_1)

# Remove "Pellet" due to structural zeros
choice_data_by_replicate_no_pellet <- choice_data_by_replicate %>%
  filter(food_item != "Pellet")

# Attempt models with replicate as fixed and random effects
model_fixed <- glmmTMB(
  n ~ location * food_item + replicate,
  family = nbinom2(),
  data = choice_data_by_replicate_no_pellet)
```

```
## Warning in finalizeTMB(TMBStruc, obj, fit, h, data.tmb.old): Model convergence
## problem; false convergence (8). See vignette('troubleshooting'),
## help('diagnose')
```

```
model_random <- glmmTMB(
  n ~ location * food_item + (1 | replicate),
  family = nbinom2(),
  data = choice_data_by_replicate_no_pellet)
```

```
## Warning in finalizeTMB(TMBStruc, obj, fit, h, data.tmb.old): Model convergence
## problem; false convergence (8). See vignette('troubleshooting'),
## help('diagnose')
```

```
# Compare models using AIC
AIC(model_fixed, model_random)
```

```
##              df      AIC
## model_fixed  10 393.7981
## model_random 10 393.7988
```

Since the models failed to converge and thus yielded unreliable
estimates, the data are instead explored visually.

Fish and Mussel were examined more closely because they were chosen
most frequently and therefore had the most data, but no clear patterns
across replicates were observed.

```
ggplot(choice_data_by_replicate, aes(x = factor(replicate), y = n, fill = location)) +
  geom_col(position = "dodge") +
  facet_wrap(~ food_item, scales = "free_y") +
  labs(
    title = "First Choice Counts by Replicate for Each Food Item",
    x = "Replicate",
    y = "Count",
    fill = "Location") +
  theme_minimal() +
  theme(
    strip.text = element_text(face = "bold"),
    axis.text.x = element_text(angle = 45, hjust = 1))
```
