# Supplementary material for: Differential captivity and experiential conditions and its impact on the behaviour and cognition of Picasso triggerfish (Rhinecanthus aculeatus)
Source: Anim Cogn. 2026 Mar 14;29(1):33. doi: 10.1007/s10071-026-02057-1 (PMC13002752; doi:10.1007/s10071-026-02057-1)
Supplement: Supplementary file 7 — Supplementary file7 (HTML 2929 KB) [file 10071_2026_2057_MOESM7_ESM.html]

Emergence Test Analysis


# Emergence Test Analysis

#### Cait Newport

#### 2025-06-24

# 1 Project Description

This analysis is part of the data processing pipeline for the
**Emergence Test**, a behavioral assessment used in the
associated manuscript: *Behavioural Differences Across Captivity
Conditions in Triggerfish (Rhinecanthus aculeatus): Implications for
Cognitive Testing*.

The current notebook focuses on the following components of the
behavioral dataset:

- **Emergence time**: The time in seconds that it took a
  fish to emerge from a box

---

# 2 Environment Initialisation

## 2.1 Load required libraries

```
# Load necessary libraries
library(readxl)     # For reading Excel files
library(dplyr)      # For data manipulation
```

```
## 
## Attaching package: 'dplyr'
```

```
## The following objects are masked from 'package:stats':
## 
##     filter, lag
```

```
## The following objects are masked from 'package:base':
## 
##     intersect, setdiff, setequal, union
```

```
library(ggplot2)    # For plotting
library(lme4)
```

```
## Loading required package: Matrix
```

```
library(DHARMa)
```

```
## This is DHARMa 0.4.6. For overview type '?DHARMa'. For recent changes, type news(package = 'DHARMa')
```

```
library(multcomp)
```

```
## Loading required package: mvtnorm
```

```
## Loading required package: survival
```

```
## Loading required package: TH.data
```

```
## Loading required package: MASS
```

```
## 
## Attaching package: 'MASS'
```

```
## The following object is masked from 'package:dplyr':
## 
##     select
```

```
## 
## Attaching package: 'TH.data'
```

```
## The following object is masked from 'package:MASS':
## 
##     geyser
```

```
library(glmmTMB)
```

```
## Warning in checkDepPackageVersion(dep_pkg = "TMB"): Package version inconsistency detected.
## glmmTMB was built with TMB version 1.9.11
## Current TMB version is 1.9.14
## Please re-install glmmTMB from source or restore original 'TMB' package (see '?reinstalling' for more information)
```

```
library(patchwork)
```

```
## 
## Attaching package: 'patchwork'
```

```
## The following object is masked from 'package:MASS':
## 
##     area
```

```
library(emmeans)
```

```
## Welcome to emmeans.
## Caution: You lose important information if you filter this package's results.
## See '? untidy'
```

```
library(scales)      # for pseudo_log_trans()
```

## 2.2 Load data

The data has been annotated by CN in Boris and extracted to an excel
file using the process\_emergenceTest\_data.R script.

```
# Read the Excel file
data <- read_excel("/Users/user/projects/CaptiveCognition/EmergenceTest/emergence_times.xlsx")
```

## 2.3 Exploring the raw data

### 2.3.1 Mean Emergence Time and standard error for each fish

```
# Calculate summary statistics: mean and standard error (SE)
summary_data <- data %>%
  group_by(fish_id, location) %>%
  summarise(
    mean_emergence = mean(emergence_time, na.rm = TRUE),
    se_emergence = sd(emergence_time, na.rm = TRUE) / sqrt(n()),
    sd_emergence = sd(emergence_time),
  )
```

```
## `summarise()` has grouped output by 'fish_id'. You can override using the
## `.groups` argument.
```

```
fish_order <- c("F1", "F2", "F3", "F4", "F5", "F6", "F7", "F8", "F9", "F10",
                "F46", "F49", "F53", "F54", "F56", "F60", "F62", "F63", "F65", "F66")

summary_data$fish_id <- factor(summary_data$fish_id, levels = fish_order)


# Create the plot
ggplot(summary_data, aes(x = fish_id, y = mean_emergence, color = location)) +
   geom_jitter(data = data,
              aes(x = fish_id, y = emergence_time),
              color = "grey",
              size = 2,
              width = 0.2,
              alpha = 0.9) +
  geom_point(position = position_dodge(width = 0.5), size = 3) +
  geom_errorbar(aes(ymin = mean_emergence - se_emergence,
                    ymax = mean_emergence + se_emergence),
                width = 0.2,
                position = position_dodge(width = 0.5)) +
  scale_color_manual(values = c("OXFORD" = "#4C72B0", "LIRS" = "#C44E52"))+
  labs(title = "Mean Emergence Time by Fish ID and Location",
       x = "Fish ID",
       y = "Mean Emergence Time (s)") +
   theme_minimal(base_size = 14) +
   theme(
    plot.title = element_text(hjust = 0.5, face = "bold"),
    legend.position = "top",
    axis.text = element_text(size = 12))
```

Fish 56 has only two observations and one of those times is an outlier.
There are no obvious biological or sampling method reasons to exclude
this point so we will first try to fit a model with the complete
dataset.

Although we are going to try to fit a model with the full dataset, it
is useful to look at the data structure without F56 and the extreme
outlier point.

```
# Create a new dataset without the max value
data_no_F56 <- data %>%
  filter(fish_id != "F56")

# Summary without outlier
summary_data_without <- data_no_F56 %>%
  group_by(fish_id, location) %>%
  summarise(
    mean_emergence = mean(emergence_time, na.rm = TRUE),
    se_emergence = sd(emergence_time, na.rm = TRUE) / sqrt(n()),
    .groups = "drop"
  )
summary_data_without$fish_id <- factor(summary_data_without$fish_id, levels = c("F1", "F2", "F3", "F4", "F5", "F6", "F7", "F8", "F9", "F10", "F46", "F49", "F53", "F54", "F60", "F62", "F63", "F65", "F66"))

# Plot without outlier
plot_without <- ggplot(summary_data_without, aes(x = fish_id, y = mean_emergence, fill = location)) +
  geom_point(position = position_dodge(width = 0.5), size = 4, shape = 21, color = "black") +
  geom_errorbar(aes(ymin = mean_emergence - se_emergence,
                    ymax = mean_emergence + se_emergence),
                width = 0.2,
                position = position_dodge(width = 0.5)) +
  scale_fill_manual(values = c("OXFORD" = "#4C72B0", "LIRS" = "#C44E52")) +
  labs(title = "Without F56",
       x = "Fish ID",
       y = "Mean Emergence Time (s)") +
  theme_minimal()

plot_without
```

### 2.3.2 Violin plot of emergence time by fish and location

```
data_no_F56$fish_id <- factor(data_no_F56$fish_id, levels = c("F1", "F2", "F3", "F4", "F5", "F6", "F7", "F8", "F9", "F10", "F46", "F49", "F53", "F54", "F60", "F62", "F63", "F65", "F66"))


p_fish <- ggplot(data_no_F56, aes(x = fish_id, y = emergence_time, fill = location)) +
  geom_violin(alpha = 0.7, scale = "width", trim = FALSE) +
  geom_jitter(width = 0.1, alpha = 0.5, color = "black", size = 1) +
  stat_summary(fun = mean, geom = "point", shape = 21, size = 2.5, color = "black", fill = "white") +
  coord_flip() +
  scale_fill_manual(values = c("OXFORD" = "#4C72B0", "LIRS" = "#C44E52")) +
  labs(
    title = "Variance in Emergence Time by Fish",
    x = "Fish ID",
    y = "Trial Duration (s)"
  ) +
  theme_minimal() +
  theme(legend.position = "none")

# Violin plot by Location (flipped)
p_location <- ggplot(data_no_F56, aes(x = location, y = emergence_time, fill = location)) +
  geom_violin(alpha = 0.7, scale = "width", trim = FALSE) +  # No manual fill here
  geom_jitter(width = 0.1, alpha = 0.5, color = "black", size = 1) +
  stat_summary(fun = mean, geom = "point", shape = 21, size = 2.5, color = "black", fill = "white") +
  coord_flip() +
  scale_fill_manual(values = c("OXFORD" = "#4C72B0", "LIRS" = "#C44E52")) +
  labs(
    title = "Variance in Emergence Time by Location",
    x = "Location",
    y = "Trial Duration (s)"
  ) +
  theme_minimal()

# Combine plots in a vertical panel
p_fish | p_location
```

### Data distribution

```
ggplot(data_no_F56, aes(x = emergence_time, fill = location)) +
  geom_histogram(position = "stack", bins = 15, color = "black", alpha = 0.8) +
  scale_fill_manual(values = c("OXFORD" = "#4C72B0", "LIRS" = "#C44E52")) +
  labs(
    title = "Stacked Histogram of Emergence Time by Fish Group",
    x = "Emergence Time (seconds)",
    y = "Frequency",
    fill = "Fish Group"
  ) +
  theme_minimal(base_size = 14) +
  theme(
    plot.title = element_text(hjust = 0.5, face = "bold"),
    legend.position = "top",
    axis.text = element_text(size = 12)
  )
```

### 2.3.3 Raw data description summary

In the dataset, there are 10 fish per group, including F56. All fish
completed three trials each, with the exception of F56, which has only
two data points.

The distribution of emergence time is clearly non-gaussian, as
indicated by the asymmetry and spread observed in the histogram.

Both the point plots and violin plots suggest differences in mean
emergence time across individual fish, particularly among the Oxford
fish. Additionally, some individuals appear to display greater
within-fish variability, as shown by the differing widths of the violin
plots and the size of the error bars.

At the location level, fish from Oxford tend to emerge slightly later
than those from LIRS. The broader spread in the Oxford group also
suggests greater behavioural variability in that population.

As we move toward modelling these data, we will take these
characteristics into account. All models will include Fish ID to account
for repeated measures, and we will use distributions appropriate for
non-normal data—fitting models in glmmTMB. We will also explore the
effects of location as a potential fixed or random effect and consider
whether allowing group-specific variance structures improves model fit.
Given the complexity of the data, we will test several model structures
to find the most suitable approach.

# 3 Model selection

## 3.1 Testing Distributional Assumptions for GLMMs of Emergence Time

We will first try to find a model that accounts for this non-gaussian
distribution.

The Poisson and Negative Binomial distributions are intended for
count data, rather than continuous. There are some instances where they
can still fit continuous data so we are trying despite the warnings
messages when you run the model.

```
# Standard Poisson GLMM (assumes mean = variance)
glmm_pois_L<- glmmTMB(emergence_time ~ location + (1 | fish_id), family = poisson, data = data)
```

```
## Warning in glmmTMB(emergence_time ~ location + (1 | fish_id), family = poisson,
## : non-integer counts in a poisson model
```

```
# Negative Binomial GLMM (accounts for overdispersion)
glmm_nb_L <- glmmTMB(emergence_time ~ location + (1 | fish_id), data = data, family = nbinom2)
```

```
## Warning in glmmTMB(emergence_time ~ location + (1 | fish_id), data = data, :
## non-integer counts in a nbinom2 model
```

```
# Gamma GLMM with log link (good for continuous, positive, and skewed data)
glmm_gamma_L <- glmmTMB(emergence_time ~ location + (1 | fish_id), data = data, family = Gamma(link = "log"))

# Compare models using AIC (lower is better)
AIC(glmm_pois_L, glmm_nb_L, glmm_gamma_L)
```

```
##              df      AIC
## glmm_pois_L   3 496.8461
## glmm_nb_L     4 294.7146
## glmm_gamma_L  4 273.1102
```

According to AIC comparison,the model with the gamma distribution is
the best fit.

### 3.1.1 Assessing Gamma model fit using simulated residuals

We will evaluate the model fit using the DHARMa package. Simulated
residuals are used to test for key assumption violations—including
overdispersion, zero-inflation, and non-random residual
structure—through diagnostic plots and statistical tests.

```
# Simulate residuals for diagnostic tests
simulation_output_L1 <- simulateResiduals(fittedModel = glmm_gamma_L, n = 1000)

# Plot residuals: expected vs observed, QQ plot, and residual vs predictor diagnostics
# Look for patterns, skew, or deviations from uniformity
plot(simulation_output_L1)
```

The Gamma model fails two of the DHARMa diagnostics, so it seems like
just changing the data distribution is not enough to produce a model
that fits our data.

## 3.2 Testing if data transformations improves model fit

Another option for handling skewed data is to transform the data
using Log, Square Root, or Cube Root transformations.

```
# Apply transformations
transformed_data <- data %>%
  mutate(
    emergence_time_log = log(emergence_time + 1),        # log(x + 1) to avoid log(0)
    emergence_time_sqrt = sqrt(emergence_time),
    emergence_time_cube = emergence_time^(1/3)
  )

# Plot original
p1 <- ggplot(transformed_data, aes(x = emergence_time)) +
  geom_histogram(bins = 30, fill = "#69b3a2") +
  labs(title = "Original", x = "Trial Duration", y = "Count") +
  theme_minimal()

# Plot log-transformed
p2 <- ggplot(transformed_data, aes(x = emergence_time_log)) +
  geom_histogram(bins = 30, fill = "#404080") +
  labs(title = "Log(x + 1) Transformed", x = "Log Trial Duration", y = "Count") +
  theme_minimal()

# Plot sqrt-transformed
p3 <- ggplot(transformed_data, aes(x = emergence_time_sqrt)) +
  geom_histogram(bins = 30, fill = "#ffa07a") +
  labs(title = "Sqrt(x) Transformed", x = "Sqrt Trial Duration", y = "Count") +
  theme_minimal()

# Plot cube root-transformed
p4 <- ggplot(transformed_data, aes(x = emergence_time_cube)) +
  geom_histogram(bins = 30, fill = "#8b0000") +
  labs(title = "Cube Root Transformed", x = "Cube Root Trial Duration", y = "Count") +
  theme_minimal()

# Combine plots
(p1 | p2) / (p3 | p4)
```

Of all the transformations, the log transform appears to be the
best.

### 3.2.1 Create a model with log transformed data

```
# Models with transformed data
# Model with log transformed trial_duration 
model_log <- glmmTMB(emergence_time_log ~ location + (1 | fish_id), data = transformed_data)

# Simulate residuals for diagnostic tests
simulation_output_Log <- simulateResiduals(fittedModel = model_log, n = 1000)

# Plot residuals: expected vs observed, QQ plot, and residual vs predictor diagnostics
# Look for patterns, skew, or deviations from uniformity
plot(simulation_output_Log)
```

The log model does not pass the DHARMa tests.

## 3.3 Fitting models that account for differences in fish and location variance

Here we are trying to find a model that will explain how Emergence
Time is influenced by location, while accounting for variability between
individual fish.

Below are the best theoretical models, but we also tried other
distributions just in case (e.g. Inverse Gaussian, Poisson, Negative
Binomial). None of those fit the data at all.

```
# Models with Gamma distribution
# Note that these models use non-transformed data because the Gamma distriution transforms it.

# Model 1: Gamma GLMM with Fish-specific Intercepts and Location Slopes (Uncorrelated)
# - This model allows for fish-specific intercepts and location slopes
# - Assumes no correlation between the intercept and slope.
# - Uses the raw (untransformed) emergence_time with Gamma distribution and log link
gamma_random_slopes <- glmmTMB(emergence_time ~ location + (1 + location || fish_id),
  data = data, family = Gamma(link = "log"))

# Model 2: Gamma GLMM with Fish-specific Residual Variance (Dispersion Model)
# - Uses dispformula = ~ fish_id to allow each fish to have a different dispersion (variance), not just mean
# - Fish can differ in how variable their emergence times are, even if their mean response is similar
gamma_variable_dispersion <- glmmTMB(emergence_time ~ location + (1 | fish_id),
  dispformula = ~ fish_id,
  family = Gamma(link = "log"), data = data)
```

```
# Models with log-transformed data

# Model 3: Model with uncorrelated random effects
# - The model allows fish to vary in their mean log-emergence time (intercept).
# - It also allows fish to vary in how they respond to location (slope).
log_random_slopes <- glmmTMB(emergence_time_log ~ location + (1 + location || fish_id),
  data = transformed_data)
 
# Model 4: GLMM with Fish-specific Residual Variance (Dispersion Model)
# - Allow each fish to have a different dispersion (variance), not just mean
# - Fish can differ in how variable their emergence times are, even if their mean response is similar
log_variable_dispersion_fish <- glmmTMB(emergence_time_log ~ location + (1 | fish_id),
  dispformula = ~ fish_id,
  data = transformed_data)

# Model 5: GLMM with Homogeneous Residual Variance
# - Models log-emergence time as a function of location with a random intercept for fish_id
# - Assumes all fish share the same residual variance (dispersion)
# - Uses dispformula = ~ 1 to model constant dispersion across individuals
# - The purpose of this model is to compare to Model 4.
log_homogenous_dispersion <- glmmTMB(emergence_time_log ~ location + (1 | fish_id),
  dispformula = ~ 1,
  data = transformed_data)

# Model 6: GLMM with Location-specific Residual Variance
log_variable_dispersion_location <- glmmTMB(emergence_time_log ~ location + (1 | fish_id),
  dispformula = ~ location,
  data = transformed_data)
```

Compare model fits via AIC

```
# Lower AIC indicates better model fit while accounting for complexity
AIC(gamma_random_slopes, gamma_variable_dispersion, log_random_slopes, log_variable_dispersion_fish,log_homogenous_dispersion,log_variable_dispersion_location)
```

```
##                                  df       AIC
## gamma_random_slopes               5 257.80452
## gamma_variable_dispersion        23 268.35266
## log_random_slopes                 5 111.92604
## log_variable_dispersion_fish     23  95.81971
## log_homogenous_dispersion         4 124.62016
## log_variable_dispersion_location  5 104.21530
```

Results of the AIC comparison suggests that the
**log\_variable\_dispersion\_fish** is the best fitting model. A close
second is log\_variable\_dispersion\_location.

If we compare each model to Model 5 with Homogenous dispersion, both
are significantly different.

```
anova(log_variable_dispersion_fish,log_homogenous_dispersion)
```

```
## Data: transformed_data
## Models:
## log_homogenous_dispersion: emergence_time_log ~ location + (1 | fish_id), zi=~0, disp=~1
## log_variable_dispersion_fish: emergence_time_log ~ location + (1 | fish_id), zi=~0, disp=~fish_id
##                              Df    AIC    BIC logLik deviance Chisq Chi Df
## log_homogenous_dispersion     4 124.62 132.93 -58.31   116.62             
## log_variable_dispersion_fish 23  95.82 143.60 -24.91    49.82  66.8     19
##                              Pr(>Chisq)    
## log_homogenous_dispersion                  
## log_variable_dispersion_fish  3.104e-07 ***
## ---
## Signif. codes:  0 '***' 0.001 '**' 0.01 '*' 0.05 '.' 0.1 ' ' 1
```

```
anova(log_variable_dispersion_location,log_homogenous_dispersion)
```

```
## Data: transformed_data
## Models:
## log_homogenous_dispersion: emergence_time_log ~ location + (1 | fish_id), zi=~0, disp=~1
## log_variable_dispersion_location: emergence_time_log ~ location + (1 | fish_id), zi=~0, disp=~location
##                                  Df    AIC    BIC  logLik deviance  Chisq
## log_homogenous_dispersion         4 124.62 132.93 -58.310  116.620       
## log_variable_dispersion_location  5 104.22 114.60 -47.108   94.215 22.405
##                                  Chi Df Pr(>Chisq)    
## log_homogenous_dispersion                             
## log_variable_dispersion_location      1  2.208e-06 ***
## ---
## Signif. codes:  0 '***' 0.001 '**' 0.01 '*' 0.05 '.' 0.1 ' ' 1
```

### 3.3.1 Test the model fit using DHARMa package

We want to test the two models that allows for fish and location to
have variable dispersion.

```
# Simulate residuals
simulation_output_log_variable_dispersion_fish <- simulateResiduals(fittedModel = log_variable_dispersion_fish, n = 1000)

# Plot diagnostics
plot(simulation_output_log_variable_dispersion_fish)
```

```
# Simulate residuals
simulation_output_log_variable_dispersion_location <- simulateResiduals(fittedModel = log_variable_dispersion_location, n = 1000)

# Plot diagnostics
plot(simulation_output_log_variable_dispersion_location)
```

Both our models pass the DHARMa test.

We will compare the models using likelihood-ratio tests

```
anova(log_variable_dispersion_fish,log_variable_dispersion_location)
```

```
## Data: transformed_data
## Models:
## log_variable_dispersion_location: emergence_time_log ~ location + (1 | fish_id), zi=~0, disp=~location
## log_variable_dispersion_fish: emergence_time_log ~ location + (1 | fish_id), zi=~0, disp=~fish_id
##                                  Df    AIC   BIC  logLik deviance  Chisq Chi Df
## log_variable_dispersion_location  5 104.22 114.6 -47.108   94.215              
## log_variable_dispersion_fish     23  95.82 143.6 -24.910   49.820 44.396     18
##                                  Pr(>Chisq)    
## log_variable_dispersion_location               
## log_variable_dispersion_fish      0.0005063 ***
## ---
## Signif. codes:  0 '***' 0.001 '**' 0.01 '*' 0.05 '.' 0.1 ' ' 1
```

The models are significantly different. log\_variable\_dispersion\_fish
has a slightly lower AIC (delta=8.4), so we will use that model.

## 3.4 Model interpretation

### 3.4.1 Per-individual residual variances

```
summary(log_variable_dispersion_fish)
```

```
##  Family: gaussian  ( identity )
## Formula:          emergence_time_log ~ location + (1 | fish_id)
## Dispersion:                          ~fish_id
## Data: transformed_data
## 
##      AIC      BIC   logLik deviance df.resid 
##     95.8    143.6    -24.9     49.8       36 
## 
## Random effects:
## 
## Conditional model:
##  Groups   Name        Variance Std.Dev.
##  fish_id  (Intercept) 0.04047  0.2012  
##  Residual                  NA      NA  
## Number of obs: 59, groups:  fish_id, 20
## 
## Conditional model:
##                Estimate Std. Error z value Pr(>|z|)    
## (Intercept)     1.39582    0.08006  17.434  < 2e-16 ***
## locationOXFORD -0.42966    0.14104  -3.046  0.00232 ** 
## ---
## Signif. codes:  0 '***' 0.001 '**' 0.01 '*' 0.05 '.' 0.1 ' ' 1
## 
## Dispersion model:
##             Estimate Std. Error z value Pr(>|z|)    
## (Intercept) -1.03884    0.43411  -2.393 0.016710 *  
## fish_idF10  -0.50255    0.71559  -0.702 0.482502    
## fish_idF2   -1.08554    0.67668  -1.604 0.108663    
## fish_idF3   -0.34900    0.63635  -0.548 0.583394    
## fish_idF4   -0.50610    0.62875  -0.805 0.420857    
## fish_idF46  -0.44439    0.70213  -0.633 0.526793    
## fish_idF49  -0.51460    0.66741  -0.771 0.440680    
## fish_idF5   -0.15874    0.61283  -0.259 0.795611    
## fish_idF53   0.20931    0.60340   0.347 0.728677    
## fish_idF54   0.18555    0.60646   0.306 0.759634    
## fish_idF56   2.22323    0.66530   3.342 0.000833 ***
## fish_idF6   -0.84467    0.65857  -1.283 0.199640    
## fish_idF60  -0.21672    0.61472  -0.353 0.724425    
## fish_idF62   0.19574    0.60752   0.322 0.747309    
## fish_idF63   0.01143    0.64520   0.018 0.985870    
## fish_idF65   1.44705    0.61648   2.347 0.018912 *  
## fish_idF66   0.74315    0.62795   1.183 0.236628    
## fish_idF7   -0.41846    0.63980  -0.654 0.513080    
## fish_idF8    0.13858    0.63081   0.220 0.826110    
## fish_idF9   -2.03359    0.66811  -3.044 0.002336 ** 
## ---
## Signif. codes:  0 '***' 0.001 '**' 0.01 '*' 0.05 '.' 0.1 ' ' 1
```

```
# Estimated marginal means. Using type = response back transforms the results
emm_fish <- emmeans(log_variable_dispersion_fish, ~ location, level = 0.95)

# Perform pairwise comparisons of location
pairwise_contrast_fish <- contrast(emm_fish, method = "pairwise")

# Summarize contrasts and include confidence intervals (set infer = c(TRUE, TRUE))
contrast_df_fish <- summary(pairwise_contrast_fish, infer = c(TRUE, TRUE)) |> as.data.frame()

# View the resulting contrast estimates with associated uncertainty
print("Model-based Means")
```

```
## [1] "Model-based Means"
```

```
contrast_df_fish
```

```
##  contrast       estimate        SE df  lower.CL  upper.CL t.ratio p.value
##  LIRS - OXFORD 0.4296611 0.1410401 36 0.1436185 0.7157036   3.046  0.0043
## 
## Confidence level used: 0.95
```

```
# Back-transform for plotting: exp(predicted) - 0.01
emm_df_fish <- summary(emm_fish) %>%
  as_tibble() %>%
  mutate(
    response_mean = exp(emmean) - 0.01, # Back-transformed
    lower_CL = exp(lower.CL) - 0.01, # Back-transformed
    upper_CL = exp(upper.CL) - 0.01) # Back-transformed
```

This model shows that there is a significant effect of Location (p =
0.00232) on emergence times, where Oxford fish have shorter emergence
times.

Three fish have significantly more variable residual variance (F56 p
> 0.01, F65 p > 0.05, F9 p > 0.05).

### 3.4.2 Per-location residual variances

```
summary(log_variable_dispersion_location)
```

```
##  Family: gaussian  ( identity )
## Formula:          emergence_time_log ~ location + (1 | fish_id)
## Dispersion:                          ~location
## Data: transformed_data
## 
##      AIC      BIC   logLik deviance df.resid 
##    104.2    114.6    -47.1     94.2       54 
## 
## Random effects:
## 
## Conditional model:
##  Groups   Name        Variance Std.Dev.
##  fish_id  (Intercept) 0.03597  0.1897  
##  Residual                  NA      NA  
## Number of obs: 59, groups:  fish_id, 20
## 
## Conditional model:
##                Estimate Std. Error z value Pr(>|z|)    
## (Intercept)     1.35593    0.07638  17.753   <2e-16 ***
## locationOXFORD  0.02382    0.20190   0.118    0.906    
## ---
## Signif. codes:  0 '***' 0.001 '**' 0.01 '*' 0.05 '.' 0.1 ' ' 1
## 
## Dispersion model:
##                Estimate Std. Error z value Pr(>|z|)    
## (Intercept)     -1.3509     0.1543  -8.754  < 2e-16 ***
## locationOXFORD   1.3018     0.2056   6.333 2.41e-10 ***
## ---
## Signif. codes:  0 '***' 0.001 '**' 0.01 '*' 0.05 '.' 0.1 ' ' 1
```

```
# Estimated marginal means. Using type = response back transforms the results
emm_location <- emmeans(log_variable_dispersion_location, ~ location, level = 0.95)

# Perform pairwise comparisons of location
pairwise_contrast_location <- contrast(emm_location, method = "pairwise")

# Summarize contrasts and include confidence intervals (set infer = c(TRUE, TRUE))
contrast_df_location <- summary(pairwise_contrast_location, infer = c(TRUE, TRUE)) |> as.data.frame()

# View the resulting contrast estimates with associated uncertainty
print("Model-based Means")
```

```
## [1] "Model-based Means"
```

```
contrast_df_location
```

```
##  contrast         estimate        SE df   lower.CL  upper.CL t.ratio p.value
##  LIRS - OXFORD -0.02382287 0.2018975 54 -0.4286029 0.3809572  -0.118  0.9065
## 
## Confidence level used: 0.95
```

```
# Back-transform for plotting: exp(predicted) - 0.01
emm_df_location <- summary(emm_location) %>%
  as_tibble() %>%
  mutate(
    response_mean = exp(emmean) - 0.01, # Back-transformed
    lower_CL = exp(lower.CL) - 0.01, # Back-transformed
    upper_CL = exp(upper.CL) - 0.01) # Back-transformed
```

This model shows that there is no significant effect of Location (p =
0.91) on emergence times.

However, Oxford fish have more variable residual variance (p <
0.001).

## 3.5 Plots

### 3.5.1 Emergence time by location

This plot uses the model Based Means from the
log\_variable\_dispersion\_fish as it had the lowest AIC value.

```
# Reorder factor levels so Oxford comes first
data_no_F56$location <- factor(data_no_F56$location, levels = c("OXFORD", "LIRS"))
emm_df_fish$location <- factor(emm_df_fish$location, levels = c("OXFORD", "LIRS"))

emergence_plot <- ggplot() +
  # Jittered individual fish-level means
  geom_jitter(data = data_no_F56, 
              aes(x = location, y = emergence_time, colour = location), 
              width = 0.15, alpha = 0.3, size = 2) +
  
  # Model-based means with CI
  geom_errorbar(data = emm_df_fish, 
                aes(x = location, ymin = lower_CL, ymax = upper_CL, colour= location), 
                width = 0.08, linewidth = 0.5) +
  
  geom_point(data = emm_df_fish, 
             aes(x = location, y = response_mean, fill=location), 
             shape = 21, size = 3, stroke = 0) +
  
  # Titles and labels
  labs(x = "Location", y = "Emergence time (seconds)") +
  
  # Aesthetic theme adjustments
  theme_minimal(base_size = 14) +
  theme(
    plot.title = element_text(hjust = 0.5, face = "bold"),
    axis.title = element_text(size = 12),
    axis.text = element_text(size = 11),
    panel.grid.major.x = element_blank(),
    panel.grid.minor = element_blank(),
    panel.grid.major.y = element_line(color = "grey90"),
    axis.line = element_line(color = "black", linewidth = 0.5),
    axis.ticks = element_line(color = "black"),
    legend.position = "none") +
  
  # Manual color and fill scales
  scale_colour_manual(values = c(OXFORD = "#002147", LIRS = "#C44E52")) +
  scale_fill_manual(values = c(OXFORD = "#002147", LIRS = "#C44E52"))
   
  
emergence_plot
```

### 3.5.2 Emergence time by individual (raw data)

```
# Ensure location is a factor with Oxford first
summary_data <- summary_data %>%
  mutate(location = factor(location, levels = c("OXFORD", "LIRS")))

# Reorder fish_id by location (Oxford first) and alphabetically within location
fish_order <- summary_data %>%
  distinct(fish_id, location) %>%
  arrange(location, fish_id) %>%
  pull(fish_id)

# Apply the ordering to the fish_id factor
summary_data <- summary_data %>%
  mutate(fish_id = factor(fish_id, levels = fish_order))

# Plot
emergence_by_ind <- ggplot(summary_data, aes(x = fish_id, y = mean_emergence, fill = location)) +
  # Mean points
  geom_point(position = position_dodge(width = 0.5),size = 4, shape = 21, colour = "black") +
  # Error bars
  geom_errorbar(aes(ymin = mean_emergence - se_emergence ,ymax = mean_emergence + se_emergence),
    width = 0.2,position = position_dodge(width = 0.5)) +

  # Pseudo-log y-axis for better visibility of small & large values
  scale_y_continuous(trans  = pseudo_log_trans(base = 10, sigma = 0.5),
    breaks = c(1, 2, 5, 10, 20, 50, 100, 200),labels = comma_format()) +
  
  # Manual fill colors by location
  scale_fill_manual(values = c("OXFORD" = "#002147", "LIRS" = "#C44E52")) +

  # Labels and titles
  labs(x = "Fish ID",y = "Mean emergence time (s, pseudo-log)", fill="Location") +

  # Clean minimal theme with custom tweaks
  theme_minimal() +
  theme(plot.title      = element_text(hjust = 0.5, face = "bold"),
    axis.title      = element_text(size = 12),
    axis.text       = element_text(size = 11),
    panel.grid.major.x = element_blank(),
    panel.grid.minor   = element_blank(),
    panel.grid.major.y = element_line(color = "grey90"),
    axis.line          = element_line(color = "black", linewidth = 0.5),
    axis.ticks         = element_line(color = "black"),
    legend.position    = "right")

emergence_by_ind
```

### 3.5.3 Save plot

Combine plots

```
combined_plot <- (emergence_plot | emergence_by_ind) +
  plot_layout(guides = "collect", widths = c(1, 2))

combined_plot <- combined_plot + plot_annotation(tag_levels = 'A')

combined_plot
```

```
ggsave(
  filename = "emergence_plot.png",
  plot = combined_plot,
  path = "/Users/user/projects/CaptiveCognition/EmergenceTest",
  width = 12, height = 4, dpi = 600, units = "in")
```
