# Supplementary material for: Differential captivity and experiential conditions and its impact on the behaviour and cognition of Picasso triggerfish (Rhinecanthus aculeatus)
Source: Anim Cogn. 2026 Mar 14;29(1):33. doi: 10.1007/s10071-026-02057-1 (PMC13002752; doi:10.1007/s10071-026-02057-1)
Supplement: Supplementary file 8 — Supplementary file8 (HTML 3188 KB) [file 10071_2026_2057_MOESM8_ESM.html]

Cylinder Test Analysis


# Cylinder Test Analysis

#### Cait Newport

#### 2025-06-12

# 1 Project Description

This analysis is part of the data processing pipeline for the
**Cylinder Test**, a behavioral assessment used in the
associated manuscript: *Behavioural Differences Across Captivity
Conditions in Triggerfish (Rhinecanthus aculeatus): Implications for
Cognitive Testing*.

The current notebook focuses on the following components of the
behavioral dataset:

- **Pass Rate**: Number of fish to pass each Task
  Stage.
- **Trial duration**: Total duration during which the
  fish remained in a hiding state.

## 1.1 Set up the Environment

### 1.1.1 Load packages

```
## ── Attaching core tidyverse packages ──────────────────────── tidyverse 2.0.0 ──
## ✔ dplyr     1.1.4     ✔ readr     2.1.5
## ✔ forcats   1.0.0     ✔ stringr   1.5.1
## ✔ ggplot2   3.5.1     ✔ tibble    3.2.1
## ✔ lubridate 1.9.3     ✔ tidyr     1.3.1
## ✔ purrr     1.0.2     
## ── Conflicts ────────────────────────────────────────── tidyverse_conflicts() ──
## ✖ dplyr::filter() masks stats::filter()
## ✖ dplyr::lag()    masks stats::lag()
## ℹ Use the conflicted package (<http://conflicted.r-lib.org/>) to force all conflicts to become errors
```

```
## Warning in checkDepPackageVersion(dep_pkg = "TMB"): Package version inconsistency detected.
## glmmTMB was built with TMB version 1.9.11
## Current TMB version is 1.9.14
## Please re-install glmmTMB from source or restore original 'TMB' package (see '?reinstalling' for more information)
```

```
## Loading required package: Matrix
## 
## Attaching package: 'Matrix'
## 
## The following objects are masked from 'package:tidyr':
## 
##     expand, pack, unpack
## 
## 
## Attaching package: 'lmerTest'
## 
## The following object is masked from 'package:lme4':
## 
##     lmer
## 
## The following object is masked from 'package:stats':
## 
##     step
## 
## This is DHARMa 0.4.6. For overview type '?DHARMa'. For recent changes, type news(package = 'DHARMa')
## 
## Attaching package: 'nlme'
## 
## The following object is masked from 'package:lme4':
## 
##     lmList
## 
## The following object is masked from 'package:dplyr':
## 
##     collapse
## 
## Welcome to emmeans.
## Caution: You lose important information if you filter this package's results.
## See '? untidy'
```

### 1.1.2 Load data

```
# Load the Excel file

setwd("/Users/user/projects/CaptiveCognition/DetourTest")
data_P1 <- read_excel("processed_detourtest_data_annotated.xlsx", sheet = "phase_1")
data_T <- read_excel("processed_detourtest_data_annotated.xlsx", sheet = "test")
```

# 2 PART 1: Proportion of fish to pass Phase 1

First we tested how many fish passed the first Phase, and ate within
the 5 minute trial time. We found no statistical significant difference
in the number of fish that finished.

## 2.1 Summarise the data

Fish used in the experiment

```
fish_by_group <- data_P1 %>%
  dplyr::select(fish_id, location) %>%
  distinct() %>%
  arrange(location, fish_id) %>%
  group_by(location) %>%
  summarise(Fish_IDs = paste(sort(unique(fish_id)), collapse = ", ")) %>%
  pivot_wider(names_from = location, values_from = Fish_IDs)

print(fish_by_group)
```

```
## # A tibble: 1 × 2
##   LIRS                          OXFORD                                          
##   <chr>                         <chr>                                           
## 1 1, 2, 3, 4, 5, 6, 7, 8, 9, 10 46, 49, 53, 54, 56, 59, 60, 61, 62, 63, 64, 65,…
```

Extract pass rate per individual.

```
# Count replicates per fish
replicate_counts <- data_P1 %>%
  group_by(fish_id) %>%
  summarise(n_replicates = n())

print(replicate_counts)
```

```
## # A tibble: 23 × 2
##    fish_id n_replicates
##      <dbl>        <int>
##  1       1            6
##  2       2            6
##  3       3            6
##  4       4            6
##  5       5            6
##  6       6            5
##  7       7            6
##  8       8            6
##  9       9            6
## 10      10            6
## # ℹ 13 more rows
```

```
# Summarize pass/fail per fish across all trials
fish_pass_status <- data_P1 %>%
  group_by(location, fish_id) %>%
  summarise(passed = any(test_result == "pass"), .groups = "drop") %>%
  mutate(pass_status = ifelse(passed, "Pass", "Fail"))

# Count number of pass/fail fish per location
fish_pass_summary <- fish_pass_status %>%
  count(location, pass_status) %>%
  pivot_wider(names_from = pass_status, values_from = n, values_fill = 0)

# Get fish IDs that failed
fish_pass_status %>%
  filter(pass_status == "Fail")
```

```
## # A tibble: 4 × 4
##   location fish_id passed pass_status
##   <chr>      <dbl> <lgl>  <chr>      
## 1 OXFORD        46 FALSE  Fail       
## 2 OXFORD        56 FALSE  Fail       
## 3 OXFORD        59 FALSE  Fail       
## 4 OXFORD        61 FALSE  Fail
```

```
# View the result
fish_pass_summary
```

```
## # A tibble: 2 × 3
##   location  Pass  Fail
##   <chr>    <int> <int>
## 1 LIRS        10     0
## 2 OXFORD       9     4
```

## 2.2 Test for significance in pass rates

A Fisher’s Exact Test is used to test if the proportion of pass vs
fail differs by location.

```
# Build 2x2 matrix for test
fisher_matrix <- fish_pass_summary %>%
  column_to_rownames("location") %>%
  as.matrix()

fisher.test(fisher_matrix)
```

```
## 
##  Fisher's Exact Test for Count Data
## 
## data:  fisher_matrix
## p-value = 0.1045
## alternative hypothesis: true odds ratio is not equal to 1
## 95 percent confidence interval:
##  0.5550236       Inf
## sample estimates:
## odds ratio 
##        Inf
```

We found no statistical difference between the pass and failure rates
by location (p = 0.10).

# 3 PART 2: Difference in trial time between locations

We first remove trials where fish did not eat the food within the
trial time limit.

```
# Filter out rows where test_result == 'fail'
filtered_data <- data_P1 %>%
  filter(test_result != 'fail')

filtered_data %>%
  dplyr::group_by(location) %>%
  dplyr::summarise(fish_ids = paste(sort(unique(fish_id)), collapse = ", "),
                   n_fish = n_distinct(fish_id))
```

```
## # A tibble: 2 × 3
##   location fish_ids                           n_fish
##   <chr>    <chr>                               <int>
## 1 LIRS     1, 2, 3, 4, 5, 6, 7, 8, 9, 10          10
## 2 OXFORD   49, 53, 54, 60, 62, 63, 64, 65, 66      9
```

## 3.1 Explore data

### 3.1.1 Visualize trial time Distribution with Histogram

This plot shows that, like a lot of time data, our results are skewed
to the right.

```
ggplot(filtered_data, aes(x = trial_duration, fill = location)) +
  geom_histogram(position = "stack", bins = 15, color = "black", alpha = 0.8) +
  scale_fill_manual(values = c("OXFORD" = "#002147", "LIRS" = "#C44E52")) +
  labs(
    title = "Stacked Histogram of Trial Time by Fish Group",
    x = "Trial time",
    y = "Frequency",
    fill = "Fish Group") +
  theme_minimal(base_size = 14) +
  theme(plot.title = element_text(hjust = 0.5, face = "bold"),
    legend.position = "top",
    axis.text = element_text(size = 12))
```

### 3.1.2 Explore variance amongst samples

```
# Violin plot by Fish ID (flipped)
p_fish <- ggplot(filtered_data, aes(x = factor(fish_id), y = trial_duration)) +
  geom_violin(fill = "#69b3a2", alpha = 0.7, scale = "width", trim = FALSE) +
  geom_jitter(width = 0.1, alpha = 0.5, color = "black", size = 1) +
  coord_flip() +
  labs(title = "Variance in Trial Duration by Fish",
    x = "Fish ID",
    y = "Trial Duration (s)") +
  theme_minimal()

# Violin plot by Location (flipped)
p_location <- ggplot(filtered_data, aes(x = location, y = trial_duration)) +
  geom_violin(fill = "#ffa07a", alpha = 0.7, scale = "width", trim = FALSE) +
  geom_jitter(width = 0.1, alpha = 0.5, color = "black", size = 1) +
  coord_flip() +
  labs(title = "Variance in Trial Duration by Location",
    x = "Location",
    y = "Trial Duration (s)") +
  theme_minimal()

# Combine plots in a vertical panel
p_fish | p_location
```

### 3.1.3 Explore variance by replicate number

```
ggplot(filtered_data, aes(x = replicate_num, y = trial_duration, group = as.factor(fish_id), color = as.factor(fish_id))) +
  geom_line(alpha = 0.6) +
  geom_point() +
  facet_wrap(~location) +
  labs(x = "Replicate Number", y = "Trial Duration (s)",
       color = "Fish ID",
       title = "Trial Duration Across Replicates by Fish and Location") +
  theme_minimal()
```

### 3.1.4 Data transformations

Skewed data can be difficult to fit with linear models. We will first
try to transform the data using Log, Square Root, and Cube Root
transformations.

```
# Apply transformations
log_offset<-0.0001
transformed_data <- filtered_data %>%
  mutate(
    trial_duration_log = log(trial_duration + log_offset),        # log(x + 0.01) to avoid log(0)
    trial_duration_sqrt = sqrt(trial_duration),
    trial_duration_cube = trial_duration^(1/3))

# Plot original
p1 <- ggplot(transformed_data, aes(x = trial_duration)) +
  geom_histogram(bins = 30, fill = "#69b3a2") +
  labs(title = "Original", x = "Trial Duration", y = "Count") +
  theme_minimal()

# Plot log-transformed
p2 <- ggplot(transformed_data, aes(x = trial_duration_log)) +
  geom_histogram(bins = 30, fill = "#404080") +
  labs(title = "Log(x + 0.001) Transformed", x = "Log Trial Duration", y = "Count") +
  theme_minimal()

# Plot sqrt-transformed
p3 <- ggplot(transformed_data, aes(x = trial_duration_sqrt)) +
  geom_histogram(bins = 30, fill = "#ffa07a") +
  labs(title = "Sqrt(x) Transformed", x = "Sqrt Trial Duration", y = "Count") +
  theme_minimal()

# Plot cube root-transformed
p4 <- ggplot(transformed_data, aes(x = trial_duration_cube)) +
  geom_histogram(bins = 30, fill = "#8b0000") +
  labs(title = "Cube Root Transformed", x = "Cube Root Trial Duration", y = "Count") +
  theme_minimal()

# Combine plots
(p1 | p2) / (p3 | p4)
```

Although not perfect, the log transformation does the best job. We will
try to use this transformation when trying to fit a model to the
data.

## 3.2 Model Fitting: Trial Duration by Location

We aim to determine whether trial duration differs between locations,
while accounting for individual variation among fish. Since trial
duration is continuous, positive, and slightly skewed, we tested
multiple model types.

Although we explored alternative distributions (e.g., Inverse
Gaussian, Poisson, Negative Binomial), these did not fit the data well.
Below are the top-performing models based on theoretical justification
and fit.

```
# Model with raw trial duration
# - Assumes normally distributed residuals
model_basic <- glmmTMB(trial_duration ~ location + (1 | fish_id), data = filtered_data)

# -- Gamma Distribution Models --
# Gamma is suited for positive, skewed data; log link ensures positive predictions

# Basic model with Gamma
model_gamma <- glmmTMB(trial_duration ~ location + (1 | fish_id),
                     family = Gamma(link = "log"), data = filtered_data)

# Gamma model with fish-specific dispersion (variance allowed to vary by individual)
model_var_disp_fish_gam <- glmmTMB(trial_duration ~ location + (1 | fish_id), 
                         dispformula = ~ fish_id, # models heteroscedasticity
                         family = Gamma(link = "log"), data = filtered_data)

# Gamma model with location-specific dispersion (variance allowed to vary by location)
model_var_disp_location_gam <- glmmTMB(trial_duration ~ location + (1 | fish_id), 
                         dispformula = ~ location, # models heteroscedasticity
                         family = Gamma(link = "log"), data = filtered_data)
```

```
# -- Log Transformed Models (log(x+0.01)) -- 

# Basic model with log-transformed data
model_log <- glmmTMB(trial_duration_log ~ location + (1 | fish_id), data = transformed_data)

# Fish-specific Intercepts and Location Slopes (Uncorrelated)
model_random_slopes_log <- glmmTMB(trial_duration_log ~ location + (1 + location || fish_id), data = transformed_data)

# Basic model with log-transformed data
model_replicates_log <- glmmTMB(trial_duration_log ~ location + (1 | fish_id) + (1|replicate_num), 
                                data = transformed_data)

model_var_disp_replicates_log <- glmmTMB(trial_duration_log ~ location + (1 + location || fish_id), 
                                dispformula = ~ replicate_num,
                                family = gaussian(),
                                data = transformed_data)

model_replicate_slopes_log <- glmmTMB(trial_duration_log ~ location * replicate_num +  
                              (replicate_num | fish_id),  # random intercepts + slopes per fish
                              dispformula = ~ replicate_num,
                              data = transformed_data, family = gaussian())


# Model with fish-specific dispersion
model_var_disp_fish_log <- glmmTMB(trial_duration_log ~ location + (1 | fish_id), 
                         dispformula = ~ fish_id, # models heteroscedasticity
                         family = gaussian(), data = transformed_data)

# Model with location-specific dispersion
model_var_disp_location_log <- glmmTMB(trial_duration_log ~ location + (1 | fish_id), 
                         dispformula = ~ location, # models heteroscedasticity
                         family= gaussian(), data = transformed_data)

# Note that we did  try more complicated log models with dispformula and replicate_num as a random effect BUT they had model Warnings and still did not pass the later DHARMa test.
```

```
# Compare model fit using AIC
AIC(model_basic, model_gamma, model_var_disp_fish_gam, model_var_disp_location_gam, 
    model_log, model_random_slopes_log, model_replicates_log, model_var_disp_replicates_log, model_replicate_slopes_log, model_var_disp_fish_log, model_var_disp_location_log)
```

```
##                               df      AIC
## model_basic                    4 827.3790
## model_gamma                    4 450.2298
## model_var_disp_fish_gam        5 452.1830
## model_var_disp_location_gam    5 452.2169
## model_log                      4 232.9429
## model_random_slopes_log        5 234.5998
## model_replicates_log           5 228.9943
## model_var_disp_replicates_log  6 229.1171
## model_replicate_slopes_log     9 222.6642
## model_var_disp_fish_log        5 234.9110
## model_var_disp_location_log    5 234.9428
```

We have four models with very similar AIC values, and all use the
log-transformed data.

### 3.2.1 Test the model fit using DHARMa package

We will test all competing models.

```
# model_log
simulation_output <- simulateResiduals(fittedModel = model_replicates_log, n = 1000, plot=TRUE) # Simulate residuals
```

```
# model_var_disp_fish_log
simulation_output2 <- simulateResiduals(fittedModel = model_var_disp_replicates_log, n = 1000, plot=TRUE)# Simulate residuals
```

```
# model_var_disp_location_log
simulation_output3 <- simulateResiduals(fittedModel = model_replicate_slopes_log, n = 1000, plot=TRUE)# Simulate residuals
```

None of the models pass the DHARMa tests.

We know from early data explorations that some fish appear to have
very different mean trial times. We will try identifying fish with
significantly different trial times and grouping the dispersion by fish
with lower and higher mean times.

```
fish_variance <- transformed_data %>%
  group_by(fish_id) %>%
  summarise(
    n_trials = n(),
    mean_duration = mean(trial_duration_log, na.rm = TRUE),
    sd_duration = sd(trial_duration_log, na.rm = TRUE),
    var_duration = var(trial_duration_log, na.rm = TRUE)
  ) %>%
  arrange(desc(var_duration))

# Use same table, define a threshold (e.g., > Q3 + 1.5*IQR)
iqr_mean <- IQR(fish_variance$mean_duration)
q3_mean <- quantile(fish_variance$mean_duration, 0.75)
mean_cutoff <- q3_mean + 1.5 * iqr_mean

fish_variance <- fish_variance %>%
  mutate(high_mean = mean_duration > mean_cutoff)

fish_variance %>% filter(high_mean == TRUE)
```

```
## # A tibble: 2 × 6
##   fish_id n_trials mean_duration sd_duration var_duration high_mean
##     <dbl>    <int>         <dbl>       <dbl>        <dbl> <lgl>    
## 1       7        6          2.48       0.889        0.790 TRUE     
## 2      64        6          3.73       0.628        0.395 TRUE
```

```
# Join high_mean grouping info to the main data
# This labels each fish as either "high_mean" or "normal" based on mean trial duration
transformed_data <- transformed_data %>%
  left_join(
    fish_variance %>% dplyr::select(fish_id, high_mean),
    by = "fish_id"
  ) %>%
  mutate(mean_group = ifelse(high_mean == TRUE, "high_mean", "normal"))
```

It looks like two fish have a significantly higher mean. Instead of
treating all fish equally in the random effects structure, we explore
whether grouping fish by mean behavior improves model performance.

```
# Model 1: Include individual fish and replicate number as random intercepts
# - Allow the residual variance to vary depending on whether the fish is in the "high_mean" or "normal" group
model_disp_mean_group <- glmmTMB(trial_duration_log ~ location + (1 | fish_id) + (1 | replicate_num),
                                 dispformula = ~ mean_group,
                                 family = gaussian(),
                                 data = transformed_data)

# Model 2: Use mean_group (not individual fish) as the random intercept
# - Residual variance is allowed to differ by location instead of mean_group
model_disp_mean_group2 <- glmmTMB(trial_duration_log ~ location + (1 | mean_group) + (1 | replicate_num),
                                 dispformula = ~ mean_group,
                                 family = gaussian(),
                                 data = transformed_data)

AIC(model_replicate_slopes_log, model_disp_mean_group,model_disp_mean_group2)
```

```
##                            df      AIC
## model_replicate_slopes_log  9 222.6642
## model_disp_mean_group       6 220.7649
## model_disp_mean_group2      6 227.7122
```

```
simulateResiduals(model_disp_mean_group, plot = TRUE)
```

```
## Object of Class DHARMa with simulated residuals based on 250 simulations with refit = FALSE . See ?DHARMa::simulateResiduals for help. 
##  
## Scaled residual values: 0.416 0.296 0.58 0.284 0.352 0.464 0.668 0.54 0.556 0.476 0.62 0.608 0.248 0.312 0.216 0.164 0.184 0.22 0.184 0.132 ...
```

```
simulateResiduals(model_disp_mean_group2, plot = TRUE)
```

```
## Object of Class DHARMa with simulated residuals based on 250 simulations with refit = FALSE . See ?DHARMa::simulateResiduals for help. 
##  
## Scaled residual values: 0.184 0.148 0.288 0.128 0.172 0.232 0.312 0.264 0.24 0.244 0.312 0.3 0.124 0.148 0.12 0.096 0.104 0.148 0.092 0.08 ...
```

Of all the models we tested, model\_disp\_mean\_group and
model\_replicate\_slopes\_log have the lowest AIC values. We will go with
model\_replicate\_slopes\_log model.

### 3.2.2 Test the significance of the interaction between location and replicate

```
# Model used
model_replicate_slopes_log <- glmmTMB(trial_duration_log ~ location * replicate_num +  
                              (replicate_num | fish_id),  # random intercepts + slopes per fish
                              dispformula = ~ replicate_num,
                              data = transformed_data, family = gaussian())

drop1(model_replicate_slopes_log, test='Chisq')
```

```
## Single term deletions
## 
## Model:
## trial_duration_log ~ location * replicate_num + (replicate_num | 
##     fish_id)
##                        Df    AIC    LRT Pr(>Chi)   
## <none>                    222.66                   
## location:replicate_num  1 228.82 8.1521 0.004301 **
## ---
## Signif. codes:  0 '***' 0.001 '**' 0.01 '*' 0.05 '.' 0.1 ' ' 1
```

### 3.2.3 Test the significance of fish identity to the model

```
# Model used
#model_replicate_slopes_log <- glmmTMB(trial_duration_log ~ location * replicate_num +  
#                              dispformula = ~ replicate_num,
#                              (replicate_num | fish_id),  # random intercepts + slopes per fish
#                              data = transformed_data, family = gaussian())

model_replicate_slopes_log_no_fish <- glmmTMB(trial_duration_log ~ location * replicate_num,
                              dispformula = ~ replicate_num,
                              data = transformed_data, family = gaussian())

anova(model_replicate_slopes_log,model_replicate_slopes_log_no_fish)
```

```
## Data: transformed_data
## Models:
## model_replicate_slopes_log_no_fish: trial_duration_log ~ location * replicate_num, zi=~0, disp=~replicate_num
## model_replicate_slopes_log: trial_duration_log ~ location * replicate_num + (replicate_num | , zi=~0, disp=~replicate_num
## model_replicate_slopes_log:     fish_id), zi=~0, disp=~replicate_num
##                                    Df    AIC    BIC  logLik deviance  Chisq
## model_replicate_slopes_log_no_fish  6 319.82 336.19 -153.91   307.82       
## model_replicate_slopes_log          9 222.66 247.21 -102.33   204.66 103.16
##                                    Chi Df Pr(>Chisq)    
## model_replicate_slopes_log_no_fish                      
## model_replicate_slopes_log              3  < 2.2e-16 ***
## ---
## Signif. codes:  0 '***' 0.001 '**' 0.01 '*' 0.05 '.' 0.1 ' ' 1
```

## 3.3 Model interpretation

```
summary(model_replicate_slopes_log)
```

```
##  Family: gaussian  ( identity )
## Formula:          
## trial_duration_log ~ location * replicate_num + (replicate_num |      fish_id)
## Dispersion:                          ~replicate_num
## Data: transformed_data
## 
##      AIC      BIC   logLik deviance df.resid 
##    222.7    247.2   -102.3    204.7      104 
## 
## Random effects:
## 
## Conditional model:
##  Groups   Name          Variance Std.Dev. Corr  
##  fish_id  (Intercept)   0.73260  0.8559         
##           replicate_num 0.00244  0.0494   -0.33 
##  Residual                    NA      NA         
## Number of obs: 113, groups:  fish_id, 19
## 
## Conditional model:
##                              Estimate Std. Error z value Pr(>|z|)   
## (Intercept)                   0.46082    0.31656   1.456  0.14547   
## locationOXFORD                1.22183    0.45296   2.697  0.00699 **
## replicate_num                 0.04954    0.04036   1.227  0.21967   
## locationOXFORD:replicate_num -0.17742    0.05551  -3.196  0.00139 **
## ---
## Signif. codes:  0 '***' 0.001 '**' 0.01 '*' 0.05 '.' 0.1 ' ' 1
## 
## Dispersion model:
##               Estimate Std. Error z value Pr(>|z|)  
## (Intercept)   -0.40577    0.20487  -1.981   0.0476 *
## replicate_num -0.10820    0.05388  -2.008   0.0446 *
## ---
## Signif. codes:  0 '***' 0.001 '**' 0.01 '*' 0.05 '.' 0.1 ' ' 1
```

### 3.3.1 Back-transform the means (log to seconds)

## 3.4 Plot results

Calculate model-predicted means

```
# Estimated marginal means on the log scale
emm <- emmeans(model_replicate_slopes_log, ~ location | replicate_num,
               at = list(replicate_num = 1:6))
emm_df <- as.data.frame(emm)
emm_df <- emm_df %>%
  mutate(
    response_mean = exp(emmean) - log_offset,
    lower_CL = exp(lower.CL) - log_offset,
    upper_CL = exp(upper.CL) - log_offset)
```

Plot using model-predicted means. Note the log-scale so all the
points fit nicely on the plot.

```
plot_data <- transformed_data %>%
  mutate(trial_duration_raw = exp(trial_duration_log) - log_offset)

# Ensure Oxford comes first
plot_data$location <- factor(plot_data$location, levels = c("OXFORD", "LIRS"))
emm_df$location <- factor(emm_df$location, levels = c("OXFORD", "LIRS"))

# Set dodge width (adjust to control spacing)
dodge_width <- 0.4
dodge <- position_dodge(width = 0.4)

PhaseOne_trial_time <- ggplot() +
  # Raw jittered data
  geom_point(data = plot_data,
           aes(x = replicate_num, y = trial_duration_raw, color = location),
           position = dodge,
           alpha = 0.3, size = 1.5, shape = 16) +

  # Model-based error bars
  geom_errorbar(data = emm_df,
                aes(x = replicate_num, ymin = lower_CL, ymax = upper_CL, color = location),
                width = 0.15, linewidth = 0.7,
                position = position_dodge(width = 0.4)) +

  # Model-based means
  geom_point(data = emm_df,
             aes(x = replicate_num, y = response_mean, fill = location),
             shape = 21, size = 3, stroke = 0.2, color = "black",
             position = position_dodge(width = 0.4)) +
  
  # Unify legends
  guides(
  fill = guide_legend(title = "Location"),
  color = guide_legend(title = "Location")) +

  # Axes and theme
  scale_y_continuous(
    trans = "log1p",  # log(x + 1), handles 0 nicely
    breaks = c(0.5, 1, 2, 5, 10, 20),  # adjust based on your data range
    labels = scales::label_number()) +
  scale_x_continuous(breaks = 1:6) +  # Replicate ticks
  scale_color_manual(values = c(OXFORD = "#002147", LIRS = "#C44E52")) +
  scale_fill_manual(values = c(OXFORD = "#002147", LIRS = "#C44E52")) +

  labs(x = "Replicate Number",
    y = "Trial duration (seconds, log scale)",
    title = "Model Estimates and Raw Data by Location") +
  guides(fill = guide_legend(title = "Location"),
    color = guide_legend(title = "Location")) +

  theme_minimal(base_size = 14) +
  theme(
    plot.title = element_text(hjust = 0.5, face = "bold"),
    axis.title = element_text(size = 12),
    axis.text = element_text(size = 11),
    panel.grid.major.x = element_line(color = "grey90"),
    panel.grid.minor = element_blank(),
    panel.grid.major.y = element_line(color = "grey90"),
    axis.line = element_line(color = "black"),
    axis.ticks = element_line(color = "black"),
    legend.position = "right")

PhaseOne_trial_time
```

# 4 PART 3: Passing the detour test

## 4.1 Summarise Number of Fish and Replicates per Location

```
# Ensure proper data types for consistency and plotting
data_T <- data_T %>%
  mutate(
    fish_id = factor(fish_id),  # Convert fish_id to factor
    test_result = factor(test_result, levels = c("fail", "pass")),  # Set test_result as ordered factor
    replicate_num = as.integer(replicate_num))  # Ensure replicate_num is integer

# Summarise total number of unique fish and total replicates per location
summary_table <- data_T %>%
  group_by(location) %>%
  summarise(n_fish = n_distinct(fish_id), # Count of unique fish per location
    total_replicates = n()) # Total number of replicate entries

# Display the summary table
print(summary_table)
```

```
## # A tibble: 2 × 3
##   location n_fish total_replicates
##   <chr>     <int>            <int>
## 1 LIRS          9               90
## 2 OXFORD        9               90
```

```
## ---- Count Replicates per Fish at Each Location ----

# Count number of replicates for each fish within each location
replicates_per_fish <- data_T %>%
  group_by(location, fish_id) %>%
  summarise(
    n_replicates = n(),                # Number of replicates for each fish
    .groups = "drop")

# Display the per-fish replicate counts
print(replicates_per_fish)
```

```
## # A tibble: 18 × 3
##    location fish_id n_replicates
##    <chr>    <fct>          <int>
##  1 LIRS     1                 10
##  2 LIRS     2                 10
##  3 LIRS     3                 10
##  4 LIRS     4                 10
##  5 LIRS     5                 10
##  6 LIRS     6                 10
##  7 LIRS     7                 10
##  8 LIRS     8                 10
##  9 LIRS     10                10
## 10 OXFORD   49                10
## 11 OXFORD   53                10
## 12 OXFORD   54                10
## 13 OXFORD   60                10
## 14 OXFORD   62                10
## 15 OXFORD   63                10
## 16 OXFORD   64                10
## 17 OXFORD   65                10
## 18 OXFORD   66                10
```

## 4.2 Plot Detour Test Results (pass/fail)

```
# Create a combined variable for custom coloring
data_T <- data_T %>%
  mutate(
    fish_id = factor(fish_id, levels = sort(unique(as.numeric(as.character(fish_id))))),
    replicate_num = as.integer(replicate_num),
    location = factor(location),
    test_result = factor(test_result, levels = c("fail", "pass")),
    location_result = paste(location, test_result, sep = "_"))

data_T$fish_id <- factor(data_T$fish_id, levels = sort(unique(data_T$fish_id)))

# Define custom colors for each location-result combination
custom_colors <- c(
  "OXFORD_fail" = "#C44E52",    # red
  "OXFORD_pass" = "snow3",    # green
  "LIRS_fail"   = "#002147",    # blue
  "LIRS_pass"   = "#2ECC71")     # yellow

# Plot
detour_test <- ggplot(data_T, aes(x = replicate_num, y = fish_id, fill = location_result)) +
  geom_tile(color = "white") +
  scale_fill_manual(
  values = custom_colors,
  labels = c(
    "OXFORD_fail" = "Oxford – Fail",
    "OXFORD_pass" = "Oxford – Pass",
    "LIRS_fail"   = "LIRS – Fail",
    "LIRS_pass"   = "LIRS – Pass"
  ),
  name = "Location & Outcome"  # ← legend title
) +
  scale_x_continuous(breaks = unique(data_T$replicate_num)) +  # Show all replicate numbers
  scale_y_discrete(limits = rev(levels(data_T$fish_id))) +
  labs(
    title = "Test Results per Fish by Replicate and Location",
    x = "Replicate Number",
    y = "Fish ID",
    fill = "Location & Result"
  ) +
  theme_minimal() +
  theme(
    axis.text.x = element_text(angle = 0, hjust = 0.5),  # Horizontal x-axis labels
    panel.grid = element_blank()
  )

detour_test
```

# 5 Combine Plots

```
# Remove titles
detour_test_clean <- detour_test + labs(title = NULL)
PhaseOne_trial_time_clean <- PhaseOne_trial_time + labs(title = NULL)

# Combine with annotation
detour_plot <- (PhaseOne_trial_time_clean + detour_test_clean) +
  plot_annotation(tag_levels = 'A')  # Adds A, B, etc.

detour_plot
```

```
ggsave("detour_figure.png", plot = detour_plot, width = 12, height = 6, dpi = 300)
```
